# What you don't know can hurt others. A systematic review on calibration of stimulus intensity in pain research

**DOI:** 10.1097/j.pain.0000000000003588

**Published:** 2025-03-27

**Authors:** Julia Badzińska, Magdalena Żegleń, Łukasz Kryst, Przemysław Bąbel

**Affiliations:** a Doctoral School in the Social Sciences, Jagiellonian University, Kraków, Poland; b Pain Research Group, Institute of Psychology, Jagiellonian University, Kraków, Poland; c Institute of Psychology, University of the National Education Commission, Kraków, Poland; d Department of Anthropology, Faculty of Physical Education and Sport, University of Physical Culture in Kraków, Poland

**Keywords:** Calibration, Pain, Pain intensity, Pain measurement, Replication crisis, Experimental pain research

## Abstract

Calibration of pain stimuli is critical in experimental pain research because it makes it possible to adjust stimulus intensity to match individual pain sensitivity. Despite its importance, precise descriptions of calibration procedures are lacking in the literature, thus hindering the ability to replicate studies. The aim of this systematic review is to fill this gap by evaluating and categorizing calibration methods used in pain studies involving electrodermal stimuli in healthy adult volunteers. A search of 9 databases identified 51 relevant articles published between 2018 and 2024, which were analyzed regarding calibration methods and their effectiveness. The review identified 2 main calibration methods, namely the method of limits and the staircase method, along with 3 techniques, namely ascending, descending, and random calibration. The findings reveal that 69% of studies did not verify the effectiveness of their calibration processes. Among those that did, varying degrees of success were observed. The ascending calibration technique was less precise, while combined ascending and pseudorandom calibration offered better matching of stimulus intensity but still required optimization. However, the data were insufficient to definitively determine which method was the most accurate. There is still a lack of consistent approaches to reporting calibration in the literature, which can lead to difficulties in interpreting results and comparing different studies. Future research should focus on comparing these methods to identify the most effective approaches and explore factors influencing calibration success.

## Abstract 2

Supplemental Digital Content is Available in the Text.

## 1. Introduction

According to the International Association for the Study of Pain (IASP), pain should be understood as an unpleasant emotional and sensory experience. It is associated with actual or potential tissue damage or is described in the context of such damage.^63^ Pain can negatively affect patients' quality of life,^30,36^ so understanding its mechanisms and devising effective therapies is extremely valuable. It is estimated that about 21% of adults experience chronic pain, and about 7% to 8% experience high-impact chronic pain.^31,56,64^ A lack of data makes it difficult to determine the total cost of treating chronic pain, but estimates of the cost of treating chronic pain in the U.S. range from $560 billion to $635 billion per year, while across Europe the cost is about €300 billion annually.^13^

Pain research is important in expanding scientific knowledge and improving patients' quality of life because it leads to the development of more effective therapies. An interdisciplinary approach and a full understanding of pain mechanisms could significantly impact clinical practice and the development of holistic therapeutic approaches. In the context of experimental pain research, attention is often focused primarily on manipulation, which is a key component of this type of study because it allows research questions to be answered and verifies the causal relationships between variables. However, equal attention should also be given to other elements of experiments.

The calibration procedure is 1 of these elements. It consists of individually adjusting the intensity of pain stimuli to a particular participant.^2,70^ Calibration plays a key role in experimental pain research because it allows the stimulus to be tailored to the individual sensitivity of the participant, consequently affecting the reliability and accuracy of the obtained results. Pain threshold and pain tolerance appear to be stable, but the relationships between pain and temperature show variability over time,^4^ which only emphasizes the important role of calibration in pain research. However, despite its important role, there seems to be a lack of precise descriptions of calibration procedures in published articles.^70^ This represents an obstacle to the complete understanding and evaluation of calibration. Moreover, without accurate knowledge of the procedure used in a given study to individually adjust the intensity of the pain stimulus, it is challenging or even impossible to faithfully replicate anything beyond the general paradigm or the concept of manipulation.

It is worth emphasizing that the calibration process also has a crucial ethical aspect. Improper calibration, or lack thereof, can lead to the application of a pain stimulus that exceeds an individual's tolerance threshold. Such a situation would be incompatible with the International Association for the Study of Pain guidelines^42^ and the general principles of conducting research with human participants.^81^

There are various methods for individually matching the intensity of pain stimuli, each of which can lead to different effects. However, there is a lack of research that focuses on comparing these different methods in experimental studies. Various types of stimuli are used in research to induce pain, including mechanical,^26^ thermal,^51^ chemical,^25^ and electrical stimuli.^65^ Different experimental pain models represent various pain modalities and are characterized by the activation of distinct neural pathways.^58,59^ These differences in the characteristics of experimental pain models may also lead to variations in the effectiveness of individual calibration methods. For this reason, it was essential to include studies utilizing a single type of pain stimulus in this review. This systematic review focuses on studies using electrical stimuli which are distinguished by their precision in controlling the intensity, duration, and frequency of the applied stimulus.^68^

This review aims to fill the research gap by listing and evaluating the calibration methods used in experimental pain research. As a result, it will provide a better understanding of the role of calibration in experimental pain research and identify potential directions for future research. To the best of the authors' knowledge, this article is the first such systematic review.

The literature was summarized to answer the following questions: (1) What methods of calibration exist? (2) What methods of calibration are used most often? (3) Do the data presented in a given article make it possible to evaluate the effectiveness of the calibration procedure used? For example, was pain assessed after the calibration but before the experimental manipulation (ie, was a pretest performed)? (4) Are the calibration methods used in the included studies effective (ie, how accurately is the calibrated stimulus intensity able to elicit the desired pain response, measured on the pain scale)? (5) Are the calibration methods used in the included studies described in enough detail to allow for potential replication? (6) If an experiment was conducted on separate occasions (ie, after a few hours, days, weeks, or months), was a new calibration performed every time and were the thresholds rechecked?

## 2. Methods

### 2.1. Protocol and registration

This systematic review was performed according to an a priori registered protocol [PROSPERO ID: CRD42022372764]. The methods of data search and synthesis are consistent with the recommendations described in the Cochrane Handbook for Systematic Reviews.^39^

### 2.2. Literature search/databases

Nine databases were searched for relevant articles: (1) PubMed; (2) Cochrane; (3) Embase; (4) PsycINFO; (5) Web of Science; (6) ScienceDirect; (7) PsycARTICLES; (8) Scopus; (9) Academic Search Ultimate.

Initially, no restriction on the year of publication was applied, and the cut-off date for the search was 2024 (the last search was performed in February 2024). To expand the scope of the cited review studies, the eligible articles were manually checked for relevance. Only studies published in English were considered and evaluated.

### 2.3. Search strategy

Inclusion/exclusion criteria were developed using the PICOS scheme, a tool used to identify research elements for systematic reviews. PICOS is an abbreviation formed from the first letters of a group of words: Population (P), Intervention (I), Comparison (C), Outcome (O), and Study type (S).^52^ The search terms used referred to a study population of healthy adult humans (P), electrical stimuli applied to an upper extremity (I), description of calibration (C), pain measured on any scale (O), and experimental or clinical study, which was randomized (if it included a group composed of healthy volunteers) or not randomized (S).

The strategies were tailored for each database and its particular search engine (see Table 1, Supplemental Digital Content, http://links.lww.com/PAIN/C250).

### 2.4. Selection criteria

Included in the review were studies that present experimental or clinical (primary) research (specifically pain studies) published in English and that describe calibration methods for electrodermal stimuli. Only studies on healthy adult volunteers (age 18 and older) were taken into consideration.

Owing to the very large number of articles meeting the initial inclusion criteria (see 3.1 Study selection), they were later narrowed to provide a more precise and clear analysis. Therefore, the review was limited to articles published within the last 7 years (2018-2024) in which electrical stimuli were applied only to the forearm. This allowed for the collection of the most recent, relevant, and significant study results that possibly represent the current state of science.^38^ Different calibration methods may be more suitable depending on the body segment, as different areas may show varying responses to the same stimulus.^48,75^ For this reason, the decision to include procedures that apply pain stimuli exclusively to the forearm was based on accepted laboratory practice, as is followed by the research group to which the authors belong (JB, MZ, PB) and the worldwide practice in many other instances^21,73,76^ where this location has been considered 1 of the most standard ones, in the context of pain research, especially when using electrical stimuli.

Review articles, meta-analyses, other secondary studies, letters to the editor, and conference communications were not included.

### 2.5. Data extraction

Four independent reviewers (J.B., M.Z., L.K., M.W.) assessed the eligibility of the studies. In the first stage of the assessment, titles and abstracts of publications identified as potentially relevant were screened for compliance with the inclusion/exclusion criteria. In the second stage, the full texts of potentially eligible studies were reviewed using the same criteria to make a final decision regarding inclusion/exclusion. Any disagreements between the researchers were resolved by a fifth reviewer (P.B.).

Two independent reviewers (J.B., M.Z.) used a standardized, pilot-tested form to extract data from the included studies. The data extracted by them were compared to ensure accuracy. Any disagreements between the reviewers were resolved by a third researcher (P.B.). If data were insufficient for analysis or unavailable, the missing data were requested from study authors through email.

The following data were extracted from studies: (1) sample size; (2) methods used for calibration; (3) instructions given to the participants; (4) detailed descriptions of the applied stimuli (eg, single stimulus, series of stimuli); (5) duration of the stimulus/series of stimuli; (6) duration of intervals between stimuli; (7) total number of stimuli per calibration; (8) number of repetitions of the calibration procedures and at which phases of the experiment; (9) duration of intervals between successive parts of the calibration; (10) mean pain ratings during calibration; (11) type of pain assessment scale (eg, VAS, NRS); (12) comparisons between calibration stimuli (eg, duration) and the stimuli applied during the experiment/manipulation.

### 2.6. Risk of bias

Two researchers assessed the risk of bias independently (J.B., M.Z.). Disagreements between assessors were resolved by a third expert (P.B.). Depending on the design of the included studies, an appropriate tool was used to assess the risk of bias. For randomized trials, RoB was used (Cochrane risk-of-bias tool)^40^; for nonrandomized trials with a control group, NOS was used (Newcastle-Ottawa Scale)^78^; for single-arm trials, NICE was used (the scale prepared by the National Institute for Health and Care Excellence).^57^

### 2.7. Synthesis of results

The included studies were subjected to qualitative synthesis; the data are presented in tables to facilitate comparisons of the various calibration methods.

## 3. Results

### 3.1. Study selection

**Figure 1.**
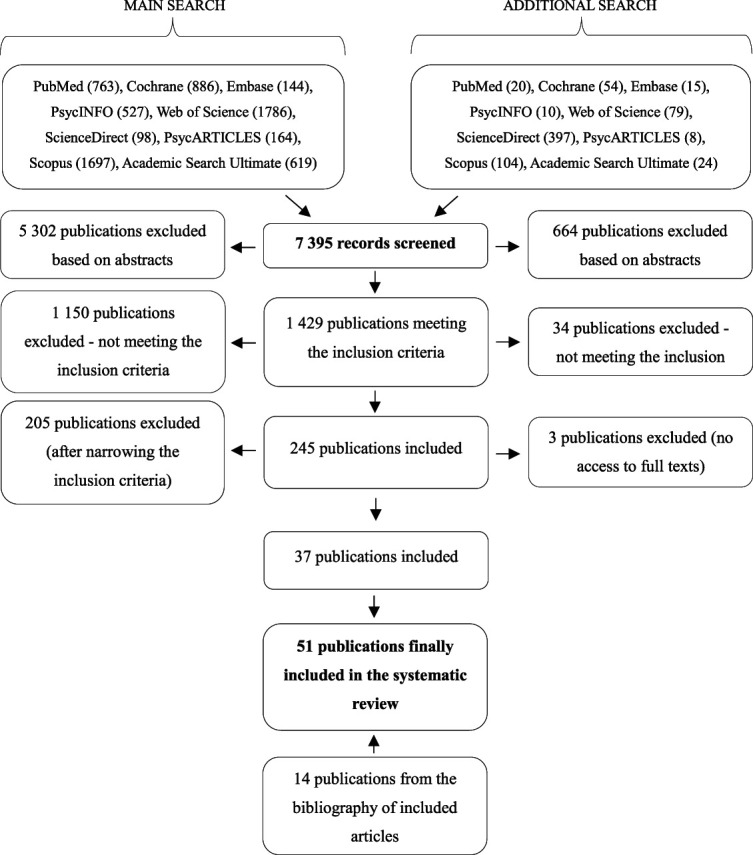
Study flow diagram.

After searching the 9 databases, 7395 articles were identified (Fig. 1). Next, they were checked for compliance with the inclusion criteria based on titles and abstracts. The 7150 studies that did not meet the criteria were rejected; 205 publications were excluded due to narrowing of the inclusion criteria, and 3 publications were excluded due to unavailability of the full texts despite attempts to contact the authors (see Table 2, Supplemental Digital Content, http://links.lww.com/PAIN/C250). An additional 14 studies were identified by checking the reference lists of the included studies, resulting in a total number of 51 articles that were included in this review.

### 3.2. Risk of bias

**Figure 2.**
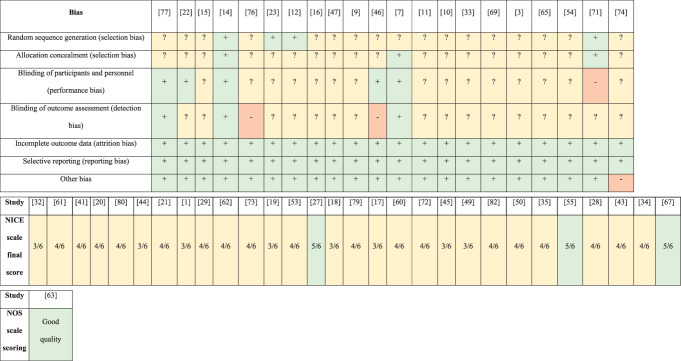
Risk-of-bias summary.

The quality of the included studies varied. An assessment of the risk of bias in each study is shown in Figure 2. Most of the studies had an unclear risk of bias due to a lack of description of the randomization method used and no information regarding allocation concealment in the study. In some studies, there was also an unclear risk of bias due to a lack of information regarding blinding of participants, personnel, and/or outcome assessment. Two studies had a high risk of bias due to a lack of blinding,^46,76^ and 1 study had a high risk of bias due to a lack of information on the blinding of volunteers.^71^ Five studies had a high risk of bias because of the inclusion of women only.^3,9,10,69,74^ In the context of this review, the unclear risk of bias in most studies does not have a significant impact on the overall evaluation of the results, their reliability, or the conclusions of this review. The criteria for assessing the risk of bias are generally unrelated to the calibration issue.

A significant number of publications also did not provide information on whether they were single- or multicenter studies. Additional sources of bias were associated with the vaguely defined inclusion and exclusion criteria or a complete lack of data in this regard. In addition, no article explicitly stated that patients were recruited consecutively.

### 3.3. Characteristics of the included studies

The included publications were based on randomized (21/51), nonrandomized with a control group (29/51), or single-arm studies (1/51). They included participants of both sexes. In total, the analyzed data included 2922 individuals (at least 994 men). As 3 articles^46,47,62^ only reported the total number of participants, it is unclear exactly how many women and men participated.

The primary outcomes of interest in the included studies were calibration methods, instructions that were given to participants, characteristics and duration of the stimuli used (eg, single stimulus/stimulus series), length of interstimulus intervals, total number of stimuli applied in the calibration, number of calibration series, duration of the intervals between each part, mean pain ratings during the calibration, type of pain rating scale (eg, VAS, NRS), and the similarity of pain stimuli applied in the calibration vs in the experiment/manipulation.

A detailed description of the studies included in the review is provided in Supplemental Digital Content (see Table 3, http://links.lww.com/PAIN/C250).

### 3.4. Main results

**Figure 3.**
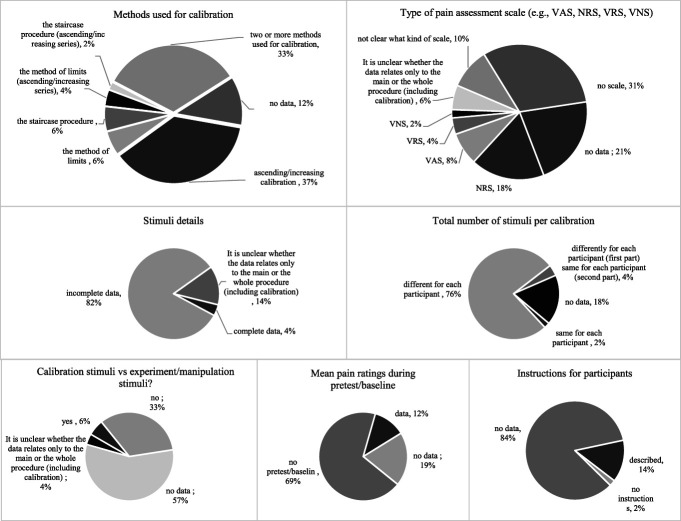
Summary of main results.

Summaries of the main results are shown in Figure 3 and Table 1.

**Table 1:** Summary of main results.

Articles	Methods used for calibration	Instructions for participants	Stimuli details	Total number of stimuli per calibration	Mean pain ratings during pretest/baseline	Type of pain assessment scale	Calibration stimuli vs experiment stimuli?
32	Increasing calibration	No data	Incomplete data	Different for each participant	No pretest/baseline	VRS	No data
41	The method of limits (increasing and decreasing)	Described	Incomplete data	Different for each participant	No pretest/baseline	No scale	No
77	Increasing calibration	No data	Incomplete data	Different for each participant	No data	VRS	No data
15	Increasing calibration	Described	Incomplete data	Different for each participant	No data	NRS	No data
14	An adaptive staircase approach (increasing calibration)	Described	Incomplete data	Different for each participant	No data	No scale	No
61	The method of limits (increasing and decreasing)	No data	*Complete data	Different for each participant	No pretest/baseline	NRS	No
22	Increasing calibration	No data	Incomplete data	Different for each participant	Data	No data	No
44	No data	No data	Incomplete data	No data	No data	VAS	No data
80	Increasing calibration	No data	Incomplete data	Different for each participant	No pretest/baseline	Not clear what kind of scale	No data
23	No data	No data	Incomplete data	Different for each participant	Data	NRS	No data
20	A staircase procedure (ascending and descending)	No data	Incomplete data	Different for each participant	No pretest/baseline	No data	Yes
76	The method of limits (increasing and decreasing)	No data	Incomplete data	Different for each participant	No pretest/baseline	No scale	No
47	Ascending calibration (1 ascending current voltage, 2 ascending time)	No data	Incomplete data	Different for each participant	No data	NRS	No
1	Increasing calibration	No data	Complete data	Different for each participant	No pretest/baseline	Not clear what kind of scale	Yes
62	No data	No data	Incomplete data	No data	No pretest/baseline	VAS	No data
46	Increasing calibration Increasing calibration by random increments	No instructions	Incomplete data	45 stimuli	No pretest/baseline	Not clear what kind of scale	No data
29	The method of limits	No data	Complete data	Different for each participant	No pretest/baseline	No data	Yes
7	Increasing calibration	No data	Incomplete data	Different for each participant	Data	VNS	No data
9	Ascending calibration	No data	Incomplete data	Different for each participant	No pretest/baseline	No data	No data
27	Increasing calibration	No data	Incomplete data	Different for each participant	No data Data	VAS	No data
53	Increasing calibration	Described	*Complete data	Different for each participant	No pretest/baseline	Not clear what kind of scale	No data
72	No data	No data	*Complete data	No data	No pretest/baseline	No data	No data
66	Increasing calibration	Described	Incomplete data	No data	No pretest/baseline	Not clear what kind of scale	No data
49	Increasing calibration	No data	Incomplete data	Different for each participant	No pretest/baseline	No data	No
82	Ascending method of limits Stimulus with a series of stimuli (ascending or descending)	No data	Incomplete data	Different for each participant Same for each participant	No pretest/baseline	VAS	No data
60	Increasing calibration	No data	Incomplete data	Different for each participant	No pretest/baseline	No scale	No
45	No data	No data	*Complete data	No data	No pretest/baseline	No data	*
12	The method of limits (ascending series)	No data	Incomplete data	Different for each participant	No data	No scale	No data
21	A staircase procedure (ascending and descending staircases)	No data	Incomplete data	Different for each participant	No pretest/baseline	No scale	No
16	Ascending calibration	No data	Incomplete data	Different for each participant	No pretest/baseline	No scale	No data
73	A staircase procedure (increasing and decreasing)	No data	Incomplete data	Different for each participant	No pretest/baseline	No scale	No data
19	The method of limits	No data	*Complete data	No data	No data	No data	No
11	Ascending calibration	No data	Incomplete data	Different for each participant	No pretest/baseline	No scale	No data
10	The method of limits (ascending)	No data	Incomplete data	Different for each participant	No pretest/baseline	No scale	No data
33	The staircase procedure	No data	Incomplete data	No data	No pretest/baseline	No data	No data
18	The staircase procedure	No data	*Complete data	Different for each participant	No pretest/baseline	No scale	No data
79	Ascending calibration	No data	Incomplete data	Different for each participant	No pretest/baseline	No scale	No data
17	The staircase procedure	No data	Incomplete data	Different for each participant	No pretest/baseline	No scale	No
69	Increasing and decreasing calibration	Described	Incomplete data	Different for each participant	No pretest/baseline	NRS	No
3	Increasing calibration	No data	Incomplete data	Different for each participant	Data	No scale	No data
50	Method of limits (ascending and descending series)	No data	Incomplete data	Different for each participant	No data	No scale	No data
65	Ascending Pseudorandom	No data	*Complete data	Different for each participant 16	Data	NRS	*
54	Staircase procedure (increasing and decreasing)	No data	Incomplete data	Different for each participant	No pretest/baseline	No scale	No
71	Familiarization phase Increasing calibration	Described	Incomplete data	Different for each participant	No pretest/baselin	*NRS	No data
74	Increasing and decreasing calibration	No data	Incomplete data	Different for each participant	No pretest/baseline	*VAS	No
35	No data	No data	Incomplete data	No data	No data	*NRS	No data
55	Familiarization phase (ascending series) Ascending series Random series	No data	Incomplete data	Different for each participant	No data	NRS	No data
28	Increasing calibration	No data	Incomplete data	Different for each participant	No pretest/baseline	NRS	No
43	Staircase procedure (increasing and decreasing)	No data	Incomplete data	No data	No pretest/baseline	No data	No data
34	Method of limits	No data	Incomplete data	Different for each participant	No pretest/baseline	No data	No
67	Increasing calibration	No data	Incomplete data	Different for each participant	No pretest/baseline	NRS	No

Methods used for calibration: the method of limits is based on applying the pain stimulus according to a predetermined research plan until the participant feels it; the staircase method is based on applying stimuli separated by a period of nonpainful stimulation, during which the research participant can assess the pain experienced after each stimulus has finished; increasing/ascending calibration consists of applying increasingly intense stimuli; decreasing/descending calibration is based on applying stimuli of decreasing intensity; random or pseudorandom calibration is based on applying stimuli with increasing and decreasing intensity in random or pseudorandom order.

Type of scale: NRS, Numeric Rating Scale; VAS, Visual Analogue Scale; VRS, Verbal Rating Scale; and VNS, Visual Numeric Scale.

* It is unclear whether the data relates only to the main or the whole procedure (including calibration).

**Figure 4.**
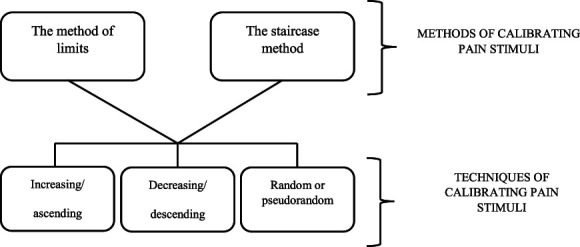
Diagram of calibration methods and techniques.

Based on the results of this systematic review, 2 methods and 3 techniques of pain stimulus calibration were identified (Fig. 4). Methods represent a general strategy for individually matching the intensity of pain stimuli, while techniques are specific ways of implementing calibration within a specific method.

First, calibrations of pain stimuli can be divided into 2 methods: (1) The method of limits is based on applying pain stimulus according to a predetermined research plan until the participant feels it (threshold of sensation, pain, pain tolerance, pain defined as a specific value, eg, 5/10 on a scale)^37^; this method was used, in some of the studies included in this review.^19,24,50,61,76^ (2) The staircase method is based on applying stimuli separated by a period of nonpainful stimulation, during which the research participant can assess the pain experienced after each stimulus has finished. The final stimulus intensity can be determined by a mathematical function or by applying a value that induced pain consistent with a predetermined research plan.^8^ This method has been used in some of the studies included in this review.^4,14,17,18,20,73^ In addition, pain stimulus calibrations can be divided into 3 techniques: (1) Increasing/ascending calibration, which consists of applying increasingly intense stimuli. This technique has been used in some of the studies included in this review^9,16,27,53,66^; (2) Decreasing/descending calibration is based on applying stimuli of decreasing intensity. It has been used in some of the studies included in this review^20,21,50,69,76^; (3) Random or pseudorandom calibration is based on applying stimuli with increasing and decreasing intensity in a random or pseudorandom order and has been used.^44,46^

To individually match the intensity of pain stimuli, 5 studies (10%) used the method of limits, 4 studies (8%) used the staircase method, and 17 studies (33%) used 2 or more methods. Calibration with increasing/ascending stimuli was used most often (19 studies, 37%). Calibrations with decreasing/descending and random/pseudorandom stimuli were not independent calibration methods: they were used as a technique for matching stimulus intensity but not as a stand-alone method. The most common aims of calibration were to determine the sensory and pain threshold and the stimulus that evokes moderate pain. Only 7 articles (14%) described the instructions that were given to participants. The most commonly used scale was the Numeric Rating Scale (NRS; 9 studies—18%), but the Visual Analogue Scale (VAS; 4 studies—8%), the Verbal Rating Scale (VRS; 2 studies—4%) and the Visual Numeric Scale (VNS; 1 study—2%) were also used. A total of 16 studies (31%) did not use a scale, while 11 (21%) did not provide information on potentially used scales. Forty-two of the analyzed publications (82%) contained incomplete data on the pain stimuli applied during calibration (information such as whether a single stimulus or a series of stimuli was applied, the duration of a single stimulus, the number of stimuli in the series, interval duration between stimuli). Only 2 of the articles fully described these parameters (4%). Nine of the articles (18%) did not specify the number of stimuli used during calibration, and the number of stimuli differed for each participant in 39 of the studies (76%). One of the studies (2%) gave each subject the same number of stimuli during calibration.

Only 6 studies (12%) provided information on the mean level of pain experienced during pretest/baseline or other procedures that were used to verify the effectiveness of the calibration before the manipulation. A total of 10 articles (19%) did not present this data, and as many as 35 of the studies (69%) did not conduct this test at all. Existing data made it possible to evaluate the effectiveness of the calibration (see 3.5 Calibration Effectiveness). In 17 of the studies (33%), the stimuli used in the calibration were different from those used in subsequent parts of the study, that is, the stimulus duration, number of stimuli, or interstimulus interval was changed without verifying whether changing these parameters would affect the accuracy of the calibrated stimuli. In 29 studies (57%), there was a lack of data on this.

### 3.5. Calibration effectiveness

It was possible to verify the effectiveness of the calibration procedures used in 5 of the studies. To do this, a comparative analysis was conducted between the planned intensity of the pain stimuli and the actual pain ratings reported by the participants in the pretest/baseline or other procedures verifying the effectiveness of the calibration. Based on the mean pain ratings (if data were collected for several groups in the articles, a common mean for the entire study was calculated), the percentage difference between planned and actual pain intensity was calculated.

Percent difference=[(|planned intensity−actual intensity|)/planned intensity]×100%

Analysis of study results indicated varying levels of effectiveness of pain stimulus calibration (Table 2). In a study by,^23^ the pain was experienced much more strongly than planned (almost double). By contrast, methods such as ascending calibration^7,27,71^ and a combination of ascending and pseudorandom calibration^65^ showed better results, although they still need to be optimized. The results indicate that ascending calibration effectively matches stimulus intensity at moderate pain levels but is less effective at higher intensities. The best results were achieved by combining different calibration methods.

**Table 2:** Evaluation of calibration effectiveness.

	23	7	27	65	71
Calibration method	No data	Increasing calibration	Increasing calibration	Ascending and pseudorandom	Increasing calibration
Planned pain intensity	1	≥5	10	5.5	10
Mean actual pain rating	2.7	4.19	5.3	4.43	6.8
Difference	1.70	0.81	4.7	1.07	3.2
Percent difference	170%	16.2%	47%	19.45%	32%

## 4. Discussion

This review summarizes the most current knowledge on the calibration methods that are used in experimental pain studies involving healthy volunteers. The authors analyzed not only the available calibration methods but also their commonness. A qualitative synthesis was performed using data from 51 studies involving 2922 participants.

Based on the results of the systematic review, 2 methods and 3 techniques of pain stimulus calibration were identified (Fig. 4). Calibration of pain stimuli can be divided into 2 methods that represent general strategies for individually matching the intensity of pain stimuli: the method of limits and the staircase method.

The method of limits is based on using a single pain stimulus whose intensity is changed until the participant feels it according to a predetermined research plan, such as a sensory/pain/tolerance threshold or at a specific point on a scale, such as 5/10 on the NRS. This method allows the target pain level to be reached quickly. A disadvantage of the method of limits may be that it is less accurate compared with the stepped method because the participant signals a certain pain level when they perceive the stimulus as sufficiently intense. In addition, pain response and the timing of reporting this sensation may not be immediate. Even a slight delay in reporting pain may lead to the administration of a higher stimulus intensity than that which actually corresponds to the pain sensation.

The staircase method is based on the use of a series of stimuli separated by a period of pain-free stimulation during which the participant assesses the pain at the end of each stimulus, which is a great advantage of this calibration method. Its disadvantages are that it can be time-consuming, especially when the participant's pain threshold is high, and it can cause participant fatigue, which can affect subsequent results.

Calibration of pain stimuli can also be divided into 3 techniques, which are specific ways of implementing calibration within a particular method: ascending/increasing calibration, descending/decreasing calibration, and random or pseudorandom calibration.

Ascending/increasing calibration involves using stimuli of increasing intensity to monitor the participant's response to increasingly intense stimuli. It may be easier to apply in practice, but its limitation is the risk of stimulus predictability, which may affect subjective pain sensations and lead to the participant reaching the point of maximum pain tolerance more quickly. It can be stressful for participants as they know that the stimuli will become increasingly painful.

Descending/decreasing calibration consists of applying stimuli at decreasing intensities to observe responses to decreasing pain. It can be less stressful for participants, but its disadvantage is the risk of not achieving or overestimating the appropriate pain level at the start of calibration.

Random or pseudorandom calibration is based on the use of stimuli of increasing and decreasing intensity in an order that is unknown to the participants. It is characterized by minimizing the predictability of the stimuli, which can lead to more objective results and allows responses to stimuli of different intensities to be tested. Its disadvantage is the risk of not achieving or overestimating the appropriate level of pain during calibration.

It is important to note that deficiencies in the description of calibration methods significantly impede the replication of experiments. Details of pain stimulus design have a crucial role in pain perception research. Information on stimulus design, duration, number of pulses, or pauses between pulses are important because they influence the pain sensations experienced by participants. However, as many as 82% of the analyzed articles were missing at least 1 of these details. In addition, studies^5,6,83^ have shown that the results of studies on pain can be affected by whether the pulse waveforms are monophasic or biphasic. Furthermore, only 14% of the analyzed studies described the instructions for the calibration procedure given to the participants. Such information may include details about the calibration process, including what participants will need to do or what equipment or tools will be used. Participants may also be given guidance on their behavior during calibration, such as proper body position, as well as instructions on how to report their sensations, such as using a specific pain scale. The description of information given to participants should be a significant part of published scientific articles. Without clearly specified instructions, each researcher may interpret and convey them differently, leading to a lack of standardization of research procedures. The difficulty in replicating conditions accurately affects researchers' ability to verify and confirm the obtained results. Missing elements make it impossible to replicate the method for individual matching of pain stimulus intensity. Consequently, if calibration methods cannot be fully replicated, the entire study cannot be replicated either; nevertheless, this conclusion is preliminary, and further studies are needed to confirm it. Future research should focus on eliminating data gaps regarding pain stimuli to not only facilitate accurate mapping of calibration procedures but also enable comparison of results from different studies.

**Figure 5.**
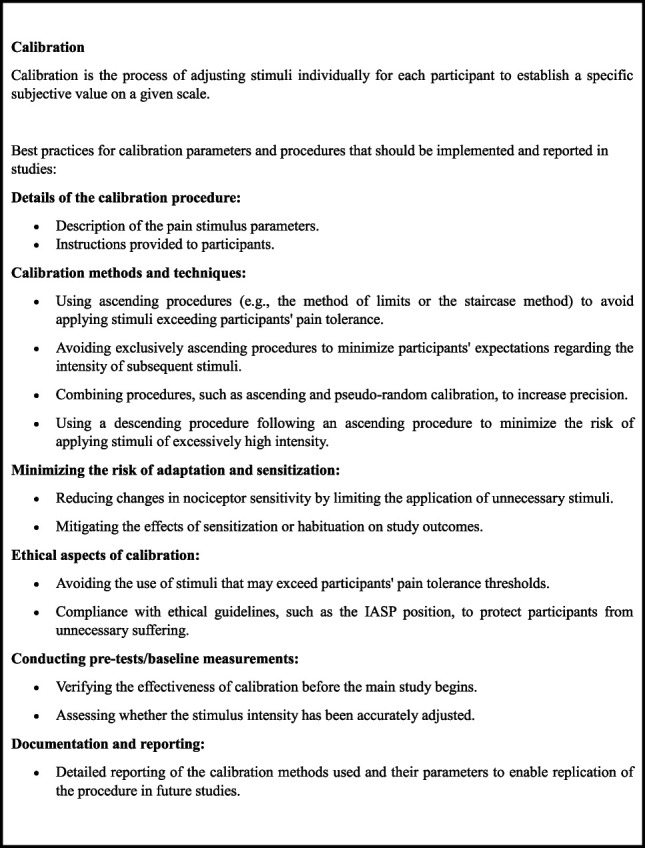
Good practices for calibration parameters and procedures.

Many of the analyzed studies lacked sufficient data to reliably assess the effectiveness of calibration. As many as 69% of the studies did not conduct a pretest/baseline or other procedures verifying the effectiveness of the calibration.^1,32,41,49,80^ This means that the researchers did not check that they were able to accurately match stimulus intensity. On the other hand, studies for which it was possible to evaluate the effectiveness of the calibration showed varying potential to produce adequate results. In the study by,^23^ the pain was experienced almost twice as strongly as planned. In contrast, methods such as ascending calibration^7,27,71^ and a combination of ascending and pseudorandom calibration^65^ have shown better matching of pain stimulus intensity, although they still need to be optimized. Although ascending calibration is a popular method for matching pain stimulus intensities, it has been shown to be insufficiently precise in matching stimulus intensities, thus leading to applying stimuli that are lower than planned. On the other hand, these results also suggest that this method is effective in reducing the risk of excessive pain stimulation. The use of more-complex calibration methods that account for individual differences in pain perception or combining ascending calibration with other techniques (eg, pseudorandom) may be necessary to increase the accuracy and effectiveness of this procedure in experimental pain studies. It is crucial to use an ascending procedure before randomly applying stimuli to avoid the risk of introducing intolerable stimuli into the experimental phase. Furthermore, using a random intensity for stimuli allows for a more precise assessment of the pain currently experienced by participants by eliminating expectations regarding the intensity of the next stimulus, something that cannot be achieved with an ascending procedure alone. Moreover, skipping pretests/baseline or other forms of verifying the effectiveness of calibration not only poses the risk of applying stimuli of unknown or misaligned intensity but may also contribute to violations of ethical standards by exposing participants to the risk of exceeding their pain tolerance threshold. There is also a real risk of applying intolerable stimuli if an ascending procedure is not implemented before the descending stimulus procedure. Such practice is against the IASP's ethical position.^42^ Conducting an ascending procedure, using methods such as ascending limits or staircase procedures, ensures that all stimuli in the subsequent phase will be tolerated by the participants. This approach allows for a more accurate determination of the pain level that should not be exceeded during the study. It should also be noted that during the application of painful stimuli, there is a risk of adaptation or sensitization of nociceptors with each successive stimulus. Therefore, it is important to minimize unnecessary changes in nociceptive sensitivity during the calibration phase to avoid disrupting the study results and to ensure greater precision in assessing pain responses. This emphasizes the importance of precise calibration in experimental pain studies. Because of the aforementioned issues, it is important to emphasize the role of pretests/baseline or other procedures verifying the effectiveness of calibration as a fundamental component of the calibration process, which is crucial for the reliability of results and for adherence to ethical principles in pain perception research. Figure 5 illustrates good practices for calibration parameters and procedures that authors should implement and report.

Although this systematic review was conducted with the highest degree of care, existing limitations that affect the interpretation of the collected data cannot be ignored. In this systematic review, all included scientific articles were published between 2018 and 2024. This restriction was intended to include the most recent studies and findings in the field of research using electrical stimulation. Furthermore, the findings of the review relate to the calibration of electrical stimuli, which are characterized by precise control intensity and simplicity of application to any area of the body. It is important to consider whether the conclusions of this systematic review can be generalized to other types of stimuli. The calibration methods and techniques extracted appear to be universal and applicable to different types of stimuli, but each may require specific adaptations of the calibration methods. Therefore, it would be worth comparing these results to assess whether the methods and techniques indicated are equally effective with other types of stimuli. In addition, the results of the calibration effectiveness assessment are based on the analysis of 5 studies, which does not yet allow for definitive conclusions regarding the most effective pain stimulus calibration method. At this point, only a suggested method can be indicated.

Calibration is a very important first step in an experimental pain study to match individual stimulus intensity. The effectiveness of calibration is a crucial component in the design of experimental pain research, where the precision of measurements directly impacts the internal validity of the results. It can be said that it is the foundation of the study, therefore the calibrated stimuli must be accurately matched. Unfortunately, there is still a lack of consistent approaches to reporting calibration in the literature, which can lead to difficulties in interpreting results and comparing different studies. Calibration should not be considered an optional phase of the experiment, but rather an essential element, the evaluation and documentation of which are necessary to ensure the credibility and reliability of scientific research. This systematic review has listed the calibration techniques used in experimental pain research. In addition, a division into different methods and techniques of calibration is proposed. The analysis has revealed that ascending calibration is effective at moderate pain levels but less so at higher pain intensities. It appears that the most effective approach is a combination of different calibration methods. This systematization allows for a more effective comparison between them and reveals gaps in the available data. It is unclear whether and how different calibration methods may affect further parts of a study. In the future, it would be worth comparing methods for individually matching the intensity of pain stimuli and determining which are the most effective and what factors may affect the effectiveness of calibration.

Further research on pain stimulus calibration is needed not only to expand knowledge but also to lay the groundwork for further development in this field. This review contributes to the improvement of research methodology. This is particularly important because the results of experimental studies provide the foundation for clinical research, which in turn influences clinical practice. Further calibration research seems necessary to develop an accurate and reliable calibration method. This will not only improve the quality of studies (it will be possible to use a stimulus of planned intensity) but will also be important from the point of view of research ethics.

## Conflict of interest statement

The authors have no conflicts of interest to declare.

## Supplemental digital content

Supplemental digital content associated with this article can be found online at http://links.lww.com/PAIN/C250.

## Supplementary Material
