# Supplementary material for: What you don't know can hurt others. A systematic review on calibration of stimulus intensity in pain research
Source: Pain. 2025 Mar 27;166(9):e137–49. doi: 10.1097/j.pain.0000000000003588 (PMC12363490; doi:10.1097/j.pain.0000000000003588)
Supplement: Supplementary file 1 [file jop-166-e137-s001.pdf]

Supplementary Table 1A. The search strategy used in the review - main search

Query	Keywords/ descriptors	PubMed	Cochrane	Embase	PsycINFO	Web of Science	Science Direct	PsycARTICLES	Scopus	Academic Search Ultimate
#1.	pain[1, 2, 4, 5, 6, 7, 8, 9]<br>'pain'/exp OR pain[3]	975451	226688	1978696	122946	879180	1,000,000+	2614	249362	462873
#2.	electric stimulation OR noxious electric stimulation OR electrocutaneous stimulation OR electric stimuli OR noxious electric stimuli OR electrocutaneous stimuli OR electrical stimulation OR noxious electrical stimulation OR electrical stimuli OR noxious electrical stimuli [1, 2, 4, 5, 7, 8, 9]<br>'electric stimulation'/exp OR 'electric stimulation' OR (electric AND ('stimulation'/exp OR stimulation)) OR 'noxious electric stimulation' OR (noxious AND electric AND ('stimulation'/exp OR stimulation)) OR 'electrocutaneous stimulation' OR (electrocutaneous AND ('stimulation'/exp OR stimulation)) OR 'electric stimuli' OR (electric AND stimuli) OR 'noxious electric stimuli' OR (noxious AND electric AND stimuli) OR 'electrocutaneous stimuli' OR (electrocutaneous AND stimuli) OR 'electrical stimulation'/exp OR 'electrical stimulation' OR (electrical AND ('stimulation'/exp OR stimulation)) OR 'noxious electrical stimulation' OR (noxious AND electrical AND ('stimulation'/exp OR stimulation)) OR 'electrical stimuli' OR (electrical AND stimuli) OR 'noxious electrical stimuli' OR (noxious AND electrical AND stimuli) [3]<br>electric stimulation OR noxious electric stimulation OR electrocutaneous stimulation OR electric stimuli [6]	201819	15632	175389	31009	126518	1,000,000+	812	40300	29584
#3.	healthy participants OR healthy subjects OR volunteers[1, 2, 4, 5, 6, 7, 8, 9]<br>(healthy AND participants OR healthy) AND subjects OR volunteers[3]	530719	158486	561487	73484	594062	242,326	1912	1417340	214784
#4.	#1 AND #2 AND #3	1865	1030	1962	542	1884	13,035	8	2801	624
#5.	#4**	785	886	159	527	1812	98	164	1722	-
#6.	#5^	763	886	144	527	1786	98	164	1697	619

Articles published on or before 2022

Supplementary Table 1B. The search strategy used in the review - additional search

Query	Keywords/ descriptors	PubMed	Cochrane	Embase	PsycINFO	Web of Science	ScienceDirect	PsycARTICLES	Scopus	Academic Search Ultimate
#1.	pain[1, 2, 4, 5, 6, 7, 8, 9] 'pain'/exp OR pain[3]	1 051 335	253 076	2 167 758	129 762	967 592	1 000 000+	17 303	2 788 376	502 184
#2.	electric stimulation OR noxious electric stimulation OR electrocutaneous stimulation OR electric stimuli OR noxious electric stimuli OR electrocutaneous stimuli OR electrical stimulation OR noxious electrical stimulation OR electrical stimuli OR noxious electrical stimuli [1, 2, 4, 5, 7, 8, 9] 'electric stimulation'/exp OR 'electric stimulation' OR (electric AND ('stimulation'/exp OR stimulation)) OR 'noxious electric stimulation' OR (noxious AND electric AND ('stimulation'/exp OR stimulation)) OR 'electrocutaneous stimulation' OR (electrocutaneous AND ('stimulation'/exp OR stimulation)) OR 'electric stimuli' OR (electric AND stimuli) OR 'noxious electric stimuli' OR (noxious AND electric AND stimuli) OR 'electrocutaneous stimuli' OR (electrocutaneous AND stimuli) OR 'electrical stimulation'/exp OR 'electrical stimulation' OR (electrical AND ('stimulation'/exp OR stimulation)) OR 'noxious electrical stimulation' OR (noxious AND electrical AND ('stimulation'/exp OR stimulation)) OR 'electrical stimuli' OR (electrical AND	207 301	17 471	185 068	31 599	135 839	262 254	1 582	43 008	32 531

	stimuli) OR 'noxious electrical stimuli' OR (noxious AND electrical AND stimuli) [3] electric stimulation OR noxious electric stimulation OR electrocutaneous stimulation OR electric stimuli [6]									
#3.	healthy participants OR healthy subjects OR volunteers[1, 2, 4, 5, 6, 7, 8, 9] (healthy AND participants OR healthy) AND subjects OR volunteers[3]	563 857	155 941	589 810	77 332	640 462	1 000 000+	13 715	1 549 314	227 880
#4.	#1 AND #2 AND #3	1 941	1139	2 043	562	1 985	13 854	138	3 092	640
#5.	#4**	20	54	15	10	79	397	8	107	24
#6.	#5^	20	54	15	10	79	397	8	104	24

Article published between 2022 and 2024

[1]PubMed; [2]Cochrane; [3]Embase; [4]PsycINFO; [5]Web of Science; [6]ScienceDirect; [7]PsycARTICLES; [8]Scopus; [9]Academic Search Ultimate;

<u>**Filters:</u>

PubMed = Clinical Study, Clinical Trial, Comparative Study, Controlled Clinical Trial, Evaluation Study, Multicenter Study, Overall, Randomized Controlled Trial, Humans

Cochrane Library =Word variations have been searched; Trials

Embase = ('clinical trial'/de OR 'comparative study'/de OR 'controlled clinical trial'/de OR 'controlled study'/de OR 'human'/de OR 'randomized controlled trial'/de) AND 'article'/it AND [embase]/lim NOT [medline]/lim

PsycINFO = Zastosuj powiązane słowa, Przeszukuj również pełny tekst artykułów, Stosowanie równoważnych tematów, Human

Web of Science = proceeding paper, early access, article

ScienceDirect = Research articles

PsycARTICLES = Zastosuj powiązane słowa, Przeszukuj również pełny tekst artykułów, Stosowanie równoważnych tematów, Czasopisma naukowe (recenzowane naukowo), Human

Scopus = Article, Human

Academic Search Ultimate none

^English;

Stimulation applied in a different method/to a different location than specified in the inclusion criteria

Jafari, H., Van de Broek, K., Plaghki, L., Vlaeyen, J. W. S., Van den Bergh, O., & Van Diest, I. (2016). Respiratory hypoalgesia? Breath-holding, but not respiratory phase modulates nociceptive flexion reflex and pain intensity. International Journal of Psychophysiology, 101, 50–58. doi:10.1016/j.ijpsycho.2016.01.005

Breimhorst, M., Sandrock, S., Fechir, M., Hausenblas, N., Geber, C., & Birklein, F. (2011). Do Intensity Ratings and Skin Conductance Responses Reliably Discriminate Between Different Stimulus Intensities in Experimentally Induced Pain? The Journal of Pain, 12(1), 61–70. doi:10.1016/j.jpain.2010.04.012

Theysohn N, Choi KE, Gizewski ER, Wen M, Rampp T, Gasser T, Dobos GJ, Forsting M, Musial F. Acupuncture-related modulation of pain-associated brain networks during electrical pain stimulation: a functional magnetic resonance imaging study. J Altern Complement Med. 2014 Dec;20(12):893-900. doi: 10.1089/acm.2014.0105.

Meyer-Frießem, C. H., Haag, L. M., Schmidt-Wilcke, T., Magerl, W., Pogatzki-Zahn, E. M., Tegenthoff, M., & Zahn, P. K. (2015). Transcutaneous spinal DC stimulation reduces pain sensitivity in humans. Neuroscience Letters, 589, 153–158. doi:10.1016/j.neulet.2015.01.029

Rhudy, J. L., Martin, S. L., Terry, E. L., DelVentura, J. L., Kerr, K. L., & Palit, S. (2012). Using multilevel growth curve modeling to examine emotional modulation of temporal summation of pain (TS-pain) and the nociceptive flexion reflex (TS-NFR). Pain, 153(11), 2274–2282. doi:10.1016/j.pain.2012.07.030

Gatzounis, R., Schrooten, M. G., Crombez, G., & Vlaeyen, J. W. (2018). Activity interruptions by pain impair activity resumption, but not more than activity interruptions by other stimuli: an experimental investigation. Pain, 159(2), 351-358.

Kim, J. H., Lee, J. U., Kim, I. H., Kim, M. Y., Kim, B., & Kim, J. (2012). Noxiousness of hypertension-related norepinephrine and upregulation of norepinephrine induced by high intensity electrical stimulation in healthy volunteers. Journal of Physical Therapy Science, 24(9), 795-800.

Geber, C., Fondel, R., Krämer, H. H., Rolke, R., Treede, R.-D., Sommer, C., & Birklein, F. (2007). Psychophysics, Flare, and Neurosecretory Function in Human Pain Models: Capsaicin Versus Electrically Evoked Pain. The Journal of Pain, 8(6), 503–514. doi:10.1016/j.jpain.2007.01.008

Rhudy, J. L., Martin, S. L., Terry, E. L., France, C. R., Bartley, E. J., DelVentura, J. L., & Kerr, K. L. (2011). Pain catastrophizing is related to temporal summation of pain but not temporal summation of the nociceptive flexion reflex. Pain, 152(4), 794–801. doi:10.1016/j.pain.2010.12.041

Pereira, G. D., Cassolato, K. M., Lazarin, P. H., Canto, T. O., Portolez, J. L. M., & Bertolini, G. R. F. (2011). Inteferential current effect, 2000Hz, on the pain induced threshold. Revista Brasileira de Medicina do Esporte, 17, 257-260.

Campbell, C. M., France, C. R., Robinson, M. E., Logan, H. L., Geffken, G. R., & Fillingim, R. B. (2008). Ethnic differences in diffuse noxious inhibitory controls. The journal of pain, 9(8), 759-766.

Dufort Rouleau, R., Lagrandeur, L., Daigle, K., Lorrain, D., Léonard, G., Whittingstall, K., & Goffaux, P. (2015). Significance of Non-phase Locked Oscillatory Brain Activity in Response to Noxious Stimuli. Canadian Journal of Neurological Sciences / Journal Canadien Des Sciences Neurologiques, 42(06), 436–443. doi:10.1017/cjn.2015.294

Enggaard, T. P., Klitgaard, N. A., & Sindrup, S. H. (2006). Specific effect of levetiracetam in experimental human pain models*. European Journal of Pain, 10(3), 193–193. doi:10.1016/j.ejpain.2005.03.011

Brodhun, C., Borelli, E., & Weiss, T. (2021). Influence of acute pain on valence rating of words. Plos one, 16(3), e0248744.

Tinnermann, A., Sprenger, C., & Büchel, C. (2022). Opioid analgesia alters corticospinal coupling along the descending pain system in healthy participants. Elife, 11, e74293.

Dounavi, M. D., Chesterton, L. S., & Sim, J. (2012). Effects of Interferential Therapy Parameter Combinations Upon Experimentally Induced Pain in Pain-Free Participants: A Randomized Controlled Trial. Physical Therapy, 92(7), 911–923. doi:10.2522/ptj.20110104

Micalos, P. S., Drinkwater, E. J., Cannon, J., Arendt-Nielsen, L., & Marino, F. E. (2008). Reliability of the nociceptive flexor reflex (RIII) threshold and association with Pain threshold. European Journal of Applied Physiology, 105(1), 55–62. doi:10.1007/s00421-008-0872-x

Bingel, U., Schoell, E., Herken, W., Büchel, C., & May, A. (2007). Habituation to painful stimulation involves the antinociceptive system. Pain, 131(1), 21–30. doi:10.1016/j.pain.2006.12.005

Karos, K., Meulders, A., Goubert, L., & Vlaeyen, J. W. S. (2018). The Influence of Social Threat on Pain, Aggression, and Empathy in Women. The Journal of Pain, 19(3), 291–300. doi:10.1016/j.jpain.2017.11.003

Stacheneder, R., Alt, L., Straube, A., & Ruscheweyh, R. (2022). Effects of Transcranial Direct Current Stimulation (t-DCS) of the Cerebellum on Pain Perception and Endogenous Pain Modulation: a Randomized, Monocentric, Double-Blind, Sham-Controlled Crossover Study. The Cerebellum, 1-9.

Yoshino, A., Okamoto, Y., Onoda, K., Shishida, K., Yoshimura, S., Kunisato, Y., … Yamawaki, S. (2012). Sadness Enhances the Experience of Pain and Affects Pain-Evoked Cortical Activities: An MEG Study. The Journal of Pain, 13(7), 628–635. doi:10.1016/j.jpain.2011.12.005

Hauck, M., Lorenz, J., & Engel, A. K. (2007). Attention to painful stimulation enhances γ-band activity and synchronization in human sensorimotor cortex. Journal of Neuroscience, 27(35), 9270-9277.

Thomaidou, M. A., Veldhuijzen, D. S., Meulders, A., & Evers, A. W. M. (2021). An experimental investigation into the mediating role of pain-related fear in boosting nocebo hyperalgesia. Pain, 162(1), 287.

Palit, S., Kerr, K. L., Kuhn, B. L., Terry, E. L., DelVentura, J. L., Bartley, E. J., … Rhudy, J. L. (2013). Exploring pain processing differences in Native Americans. Health Psychology, 32(11), 1127–1136. doi:10.1037/a0031057

Duranti, R., Pantaleo, T., Bellini, F., Bongianni, F., & Scano, G. (1991). Respiratory responses induced by the activation of somatic nociceptive afferents in humans. Journal of Applied Physiology, 71(6), 2440–2448. doi:10.1152/jappl.1991.71.6.2440

Jure, F. A., Arguissain, F. G., Biurrun Manresa, J. A., Graven-Nielsen, T., & Andersen, O. K. (2020). Stimulus predictability moderates the withdrawal strategy in response to repetitive noxious stimulation in humans. Journal of Neurophysiology, 123(6), 2201-2208.

Meissner, W., Weiss, T., Trippe, R. H., Hecht, H., Krapp, C., & Miltner, W. H. (2004). Acupuncture Decreases Somatosensory Evoked Potential Amplitudes to Noxious Stimuli in Anesthetized Volunteers. Anesthesia & Analgesia, 141–147. doi:10.1213/01.ane.0000096191.07

Fechir, M., Breimhorst, M., Kritzmann, S., Geber, C., Schlereth, T., Baier, B., & Birklein, F. (2012). Naloxone inhibits not only stress-induced analgesia but also sympathetic activation and baroreceptor-reflex sensitivity. European Journal of Pain, 16(1), 82–92.

ARENDTNIELSEN, L., FROKJAR, J., STAAHL, C., GRAVENNIELSEN, T., HUGGINS, J., SMART, T., & DREWES, A. (2007). Effects of Gabapentin on Experimental Somatic Pain and Temporal Summation. Regional Anesthesia and Pain Medicine, 32(5), 382–388. doi:10.1016/j.rapm.2007.05.002

Taniguchi, T., Kinukawa, T. A., Takeuchi, N., Sugiyama, S., Nishihara, M., Nishiwaki, K., & Inui, K. (2022). A Minimally Invasive Method for Observing Wind-Up of Flexion Reflex in Humans: Comparison of Electrical and Magnetic Stimulation. Frontiers in neuroscience, 16.

Traxler, J., Madden, V. J., Moseley, G. L., & Vlaeyen, J. W. (2019). Modulating pain thresholds through classical conditioning. PeerJ, 7, e6486.

Haller, Y., Gantenbein, A. R., Willimann, P., Spahn, D. R., & Maurer, K. (2014). Systemic ropivacaine diminishes pain sensitization processes: a randomized, double-blinded, placebo-controlled, crossover study in healthy volunteers. Pain and therapy, 3(1), 45-58.

Miller Jr, C. R., & Webers, R. L. (1990). A pilot study: The effects of ice massage on an individual's pain tolerance level to electrical stimulation. Journal of Orthopaedic & Sports Physical Therapy, 12(3), 105-110.

Krishnan, S., Salter, A., Sullivan, T., Gentgall, M., White, J., & Rolan, P. (2012). Comparison of pain models to detect opioid-induced hyperalgesia.

Drewes, A. M., Nielsen, K. D., Rasmussen, C., Arima, T., Svensson, P., Rössel, P., & Arendt-Nielsen, L. (2000). The Effects of Controlled Delta Sleep Deprivation on Experimental Pain in Healthy Subjects. Journal of Musculoskeletal Pain, 8(3), 49–67.

Vecchiet, L., Galletti, R., Giamberardino, M. A., Dragani, L., & Marini, F. (1988). Modifications of Cutaneous, Subcutaneous, and Muscular Sensory and Pain Thresholds After the Induction of an Experimental Algogenic Focus in the Skeletal Muscle. The Clinical Journal of Pain, 4(1), 55–60.

Zachariae, R., Andersen, O. K., Bjerring, P., Jørgensen, M. M., & Arendt-Nielsen, L. (1998). Effects of an opioid antagonist on pain intensity and withdrawal reflexes during induction of hypnotic analgesia in high- and low-hypnotizable volunteers. European Journal of Pain, 2(1), 25–34. doi:10.1016/s109

Vossen, H. G., van Breukelen, G. J., van Os, J., Hermens, H. J., & Lousberg, R. (2011). Association between event-related potentials and pain ratings: Not as straightforward as often thought. Journal of Psychophysiology, 25(1), 18.

Skouteri, I., Staikou, C., Sarantopoulos, C., Siafaka, I., & Fassoulaki, A. (2007). Local application of halothane, isoflurane or sevoflurane increases the response to an electrical stimulus in humans. Acta Anæsthesiologica Belgica, 58(3), 169.

Rooney, J. G., Currier, D. P., & Nitz, A. J. (1992). Effect of Variation in the Burst and Carrier Frequency Modes of Neuromuscular Electrical Stimulation on Pain Perception of Healthy Subjects. Physical Therapy, 72(11), 800–806. doi:10.1093/ptj/72.11.800

LUNDBERG, L. E. R., JØRUM, E., HOLM, E., & TOREBJÖRK, H. E. (1992). Intra-neural electrical stimulation of cutaneous nociceptive fibres in humans: effects of different pulse patterns on magnitude of pain. Acta Physiologica Scandinavica, 146(1), 41–48. doi:10.1111/j.1748-1716.1992.tb09391.x

Olofsen, E., Romberg, R., Bijl, H., Mooren, R., Engbers, F., Kest, B., & Dahan, A. (2005). Alfentanil and placebo analgesia: no sex differences detected in models of experimental pain. The Journal of the American Society of Anesthesiologists, 103(1), 130-139.

Stacher, G., Abatzi, T.-A., Schulte, F., Schneider, C., Stacher-Janotta, G., Gaupmann, G., … Steinringer, H. (1988). Naloxone does not alter the perception of pain induced by electrical and thermal stimulation of the skin Uin healthy humans. Pain, 34(3), 271–276. doi:10.1016/0304-3959(88)90122-4

Svensson, Bakke, Michler, Arendt-nielsen, & Møller. (1998). Trigeminal responses evoked by painful electrical stimulation. Journal of Oral Rehabilitation, 25(2), 128–134. doi:10.1046/j.1365-2842.1998.00218.x

Olesen, A. E., Staahl, C., Ali, Z., Drewes, A. M., & Arendt-Nielsen, L. (2007). Effects of paracetamol combined with dextromethorphan in human experimental muscle and skin pain. Basic & clinical pharmacology & toxicology, 101(3), 172-176.

Petersen-Felix, S., Luginbühl, M., Schnider, T. W., Curatolo, M., Arendt-Nielsen, L., & Zbinden, A. M. (1998). Comparison of the analgesic potency of xenon and nitrous oxide in humans evaluated by experimental pain. British journal of anaesthesia, 81(5), 742-747.

Yuan, W., Dan, L., Netra, R., Shaohui, M., Chenwang, J., & Ming, Z. (2013). A pharmaco-fMRI study on pain networks induced by electrical stimulation after sumatriptan injection. Experimental brain research, 226(1), 15-24.

Martin, F., Fletcher, D., Chauvin, M., & Bouhassira, D. (2007). Constitutive cyclooxygenase-2 is involved in central nociceptive processes in humans. The Journal of the American Society of Anesthesiologists, 106(5), 1013-1018.

France, C. R., al' Absi, M., Ring, C., France, J. L., Harju, A., & Wittmers, L. E. (2007). Nociceptive flexion reflex and pain rating responses during endogenous opiate blockade with naltrexone in healthy young adults. Biological Psychology, 75(1), 95–100.

Boyle, Y., Fernando, D., Kurz, H., Miller, S. R., Zucchetto, M., & Storey, J. (2014). The effect of a combination of gabapentin and donepezil in an experimental pain model in healthy volunteers: Results of a randomized controlled trial. Pain, 155(12), 2510–2516. doi:10.1016/j.pain.2014.09.003

Rohdewald, P., Granitzki, H. W., & Neddermann, E. (1988). Comparison of the Analgesic Efficacy of Metamizole and Tramadol in Experimental Pain. Pharmacology, 37(4), 209–217. doi:10.1159/000138468

Enggaard, T. P., Klitgaard, N. A., & Sindrup, S. H. (2006). Specific effect of levetiracetam in experimental human pain models*. European Journal of Pain, 10(3), 193–193. doi:10.1016/j.ejpain.2005.03.011

Mauermann, E., Blum, C. A., Buse, G. L., Bandschapp, O., & Ruppen, W. (2017). Time course of copeptin during a model of experimental pain and hyperalgesia: A randomised volunteer crossover trial. European Journal of Anaesthesiology | EJA, 34(5), 306-314.

Micalos, P. S., & Pak, S. C. (2011). Noneffect of Manual Needle Acupuncture on Experimental Pain Parameters in Healthy Young Men. The Journal of Alternative and Complementary Medicine, 17(1), 57–61. doi:10.1089/acm.2010.0040

Vossen, H. G. M., van Os, J., Hermens, H., & Lousberg, R. (2006). Evidence That Trait-Anxiety and Trait-Depression Differentially Moderate Cortical Processing of Pain. The Clinical Journal of Pain, 22(8), 725–729. doi:10.1097/01.ajp.0000210913.956

Chizh, B. A., Göhring, M., Tröster, A., Quartey, G. K., Schmelz, M., & Koppert, W. (2007). Effects of oral pregabalin and aprepitant on pain and central sensitization in the electrical hyperalgesia model in human volunteers. British journal of anaesthesia, 98(2), 246-254.

Huber, A., Lui, F., & Porro, C. A. (2013). Hypnotic susceptibility modulates brain activity related to experimental placebo analgesia. PAIN®, 154(9), 1509-1518.

Venancio, R. C., Pelegrini, S., Gomes, D. Q., Nakano, E. Y., & Liebano, R. E. (2013). Effects of carrier frequency of interferential current on pressure pain threshold and sensory comfort in humans. Archives of Physical Medicine and Rehabilitation, 94(1), 95-102.

Curatolo, M., Petersen-Felix, S., Gerber, A., & Arendt-Nielsen, L. (2000). Remifentanil inhibits muscular more than cutaneous pain in humans. British Journal of Anaesthesia, 85(4), 529-532.

Piletta, P., Porchet, H. C., & Dayer, P. (1991). Central analgesic effect of acetaminophen but not of aspirin. Clinical Pharmacology and Therapeutics, 49(4), 350–354. doi:10.1038/clpt.1991.40

Mauermann, E., Filitz, J., Dolder, P., Rentsch, K. M., Bandschapp, O., & Ruppen, W. (2016). Does fentanyl lead to opioid-induced hyperalgesia in healthy volunteers? A double-blind, randomized, crossover trial. Anesthesiology, 124(2), 453-463.

Chapman, C. R., Bradshaw, D. H., Donaldson, G. W., Jacobson, R. C., & Nakamura, Y. (2014). Central noradrenergic mechanisms and the acute stress response during painful stimulation. Journal of psychopharmacology, 28(12), 1135-1142.

Kinukawa, T. A., Inui, K., Taniguchi, T., Takeuchi, N., Sugiyama, S., Nishihara, M., … & Kakigi, R. (2021). Conditioned Pain Modulation: Comparison of the Effects on Nociceptive and Non-nociceptive Blink Reflex. Neuroscience, 468, 168-175.

Schneider, T., Luethi, J., Mauermann, E., Bandschapp, O., & Ruppen, W. (2020). Pain response to open label placebo in induced acute pain in healthy adult males. Anesthesiology, 132(3), 571-580.

Koppert, W., Filitz, J., Tröster, A., Ihmsen, H., Angst, M., Flor, H., … Schmelz, M. (2005). Activation of Naloxone-Sensitive and -Insensitive Inhibitory Systems in a Human Pain Model. The Journal of Pain, 6(11), 757–764. doi:10.1016/j.jpain.2005.07.002

Cheing, G., & Chan, W. (2009). Influence of choice of electrical stimulation site on peripheral neurophysiological and hypoalgesic effects. Journal of Rehabilitation Medicine, 41(6), 412–417. doi:10.2340/16501977-0350

Biurrun Manresa, J. A., Schliessbach, J., Vuilleumier, P. H., Müller, M., Musshoff, F., Stamer, U., … & Curatolo, M. (2021). Anti-nociceptive effects of oxytocin receptor modulation in healthy volunteers–A randomized, double-blinded, placebo-controlled study. European journal of pain, 25(8), 1723-1738.

Schneider, T., Zurbriggen, L., Dieterle, M., Mauermann, E., Frei, P., Mercer-Chalmers-Bender, K., & Ruppen, W. (2022). Pain response to cannabidiol in induced acute nociceptive pain, allodynia, and hyperalgesia by using a model mimicking acute pain in healthy adults in a randomized trial (CANAB I). Pain, 163(1), e62-e71.

Okkerse, P., van Amerongen, G., de Kam, M. L., Stevens, J., Butt, R. P., Gurrell, R., … & Groeneveld, G. J. (2017). The use of a battery of pain models to detect analgesic properties of compounds: a two-part four-way crossover study. British journal of clinical pharmacology, 83(5), 976-990.

Webster, D. P., Pellegrini, L., & Duffy, K. (1992). Use of transcutaneous electrical nerve stimulation for fingertip analgesia: A pilot study. Annals of Emergency Medicine, 21(12), 1472–1475. doi:10.1016/s0196-0644(05)80064-2

Lenz, H., Raeder, J., Draegni, T., Heyerdahl, F., Schmelz, M., & Stubhaug, A. (2011). Effects of COX inhibition on experimental pain and hyperalgesia during and after remifentanil infusion in humans. Pain, 152(6), 1289-1297.

Pahl, I. R., Koppert, W., Enk, C., Sittl, R., Mühldorfer, S., Hahn, E. G., … & Schwab, D. (2003). Different lipid profiles as constituencies of liquid formula diets do not influence pain perception and the efficacy of opioids in a human model of acute pain and hyperalgesia. Pain, 104(3), 519-527.

Arnold, P., Vuadens, P., Kuntzer, T., Gobelet, C., & Deriaz, O. (2008). Mirtazapine decreases the pain feeling in healthy participants. The Clinical journal of pain, 24(2), 116-119.

Takiguchi, N., & Shomoto, K. (2019). Contralateral Segmental Transcutaneous Electrical Nerve Stimulation Inhibits Nociceptive Flexion Reflex in Healthy Participants. European Journal of Pain. doi:10.1002/ejp.1373

Çıtak Karakaya, İ., Karakaya, M. G., Erğun, E., Elmalı, S., & Fırat, T. (2014). Effects of different frequencies of conventional transcutaneous electrical nerve stimulation on pressure pain threshold and tolerance. Journal of Back and Musculoskeletal Rehabilitation, 27(2), 197–201. doi:10.3233/bmr-130436

Petersen-Felix, S., Arendt-Nielsen, L., Bak, P., Fischer, M., & Zbinden, A. M. (1996). Psychophysical and electrophysiological responses to experimental pain may be influenced by sedation: comparison of the effects of a hypnotic (propofol) and an analgesic (alfentanil). British Journal of Anaesthesia, 77(2), 165–171. doi:10.1093/bja/77.2.165

Stacher, G., Steinringer, H., Schneider, S., Mittelbach, G., Winklehner, S., & Gaupmann, G. (1986). Experimental pain induced by electrical and thermal stimulation of the skin in healthy man: sensitivity to 75 and 150 mg diclofenac sodium in comparison with 60 mg codeine and placebo. British journal of clinical pharmacology, 21(1), 35-43.

Staahl, C., Upton, R., Foster, D. J. R., Christrup, L. L., Kristensen, K., Hansen, S. H., … Drewes, A. M. (2008). Pharmacokinetic-Pharmacodynamic Modeling of Morphine and Oxycodone Concentrations and Analgesic Effect in a Multimodal Experimental Pain Model. The Journal of Clinical Pharmacology, 48(5), 619–631. doi:10.1177/0091270008314465

Koppert, W., Ihmsen, H., Körber, N., Wehrfritz, A., Sittl, R., Schmelz, M., & Schüttler, J. (2005). Different profiles of buprenorphine-induced analgesia and antihyperalgesia in a human pain model. Pain, 118(1), 15–22. doi:10.1016/j.pain.2005.06.030

Tröster, A., Sittl, R., Singler, B., Schmelz, M., Schüttler, J., & Koppert, W. (2006). Modulation of remifentanil-induced analgesia and postinfusion hyperalgesia by parecoxib in humans. The Journal of the American Society of Anesthesiologists, 105(5), 1016-1023.

Zwisler, S. T., Enggaard, T. P., Noehr-Jensen, L., Mikkelsen, S., Verstuyft, C., Becquemont, L., … & Brosen, K. (2010). The antinociceptive effect and adverse drug reactions of oxycodone in human experimental pain in relation to genetic variations in the OPRM1 and ABCB1 genes. Fundamental & clinical pharmacology, 24(4), 517-524.

Stacher, G., Steinringer, H., Schmierer, G., Winklehner, S., & Schneider, C. (1982). Ceruletide increases threshold and tolerance to experimentally induced pain in healthy man. Peptides, 3(6), 955–962. doi:10.1016/0196-9781(82)90064-x

Butcher, B. E., & Carmody, J. J. (2012). Sex differences in analgesic response to ibuprofen are influenced by expectancy: A randomized, crossover, balanced placebo-designed study. European journal of pain, 16(7), 1005-1013.

Stacher, G., Steinringer, H., Winklehner, S., Mittelbach, G., & Schneider, C. (1983). Effects of graded oral doses of meptazinol and pentazocine in comparison with placebo on experimentally induced pain in healthy humans. British Journal of Clinical Pharmacology, 16(2), 149–156. doi:10.1111/j.1365-2125.1983.tb04979.x

Álvarez, D. M. C., Serrano-Muñoz, D., Fernández-Pérez, J. J., Gómez-Soriano, J., & Avendaño-Coy, J. (2022). Effect of Percutaneous Electric Stimulation with High-Frequency Alternating Currents on the Sensory-Motor System of Healthy Volunteers: A Double-Blind Randomized Controlled Study. Journal of clinical medicine, 11(7), 1832.

Engskov, A. S., Lejbman, I., & Åkeson, J. (2021). Randomized cross-over evaluation of investigator gender on pain thresholds in healthy volunteers. GMS German Medical Science, 19.

Sean, M., Coulombe-Lévêque, A., Vincenot, M., Martel, M., Gendron, L., Marchand, S., & Léonard, G. (2021). Transcutaneous electrical nerve stimulation (TENS): towards the development of a clinic-friendly method for the evaluation of excitatory and inhibitory pain mechanisms. Canadian Journal of Pain, 5(1), 56-65.

Jegindø, E. M. E., Vase, L., Skewes, J. C., Terkelsen, A. J., Hansen, J., Geertz, A. W., … & Jensen, T. S. (2013). Expectations contribute to reduced pain levels during prayer in highly religious participants. Journal of behavioral medicine, 36(4), 413-426.

Schliessbach, J., Lütolf, C., Streitberger, K., Scaramozzino, P., Arendt-Nielsen, L., & Curatolo, M. (2019). Reference values of conditioned pain modulation. Scandinavian Journal of Pain, 0(0). doi:10.1515/sjpain-2018-0356

Schrooten, M. G. S., Van Damme, S., Crombez, G., Kindermans, H., & Vlaeyen, J. W. S. (2018). Winning or not losing? The impact of non-pain goal focus on attentional bias to learned pain signals. Scandinavian Journal of Pain, 0(0). doi:10.1515/sjpain-2018-0055

Van Damme, S., Lorenz, J., Eccleston, C., Koster, E. H., De Clercq, A., & Crombez, G. (2004). Fear-conditioned cues of impending pain facilitate attentional engagement. Neurophysiologie clinique/Clinical neurophysiology, 34(1), 33-39.

Neziri, A. Y., Curatolo, M., Nüesch, E., Scaramozzino, P., Andersen, O. K., Arendt-Nielsen, L., & Jüni, P. (2011). Factor analysis of responses to thermal, electrical, and mechanical painful stimuli supports the importance of multi-modal pain assessment. Pain, 152(5), 1146–1155. doi:10.1016/j.pain.2011.01.047

Richter, M., Schroeter, C., Puensch, T., Straube, T., Hecht, H., Ritter, A., … & Weiss, T. (2014). Pain-related and negative semantic priming enhances perceived pain intensity. Pain Research and Management, 19(2), 69-74.

Adamczyk, W. M., Szikszay, T. M., Kung, T., Carvalho, G. F., & Luedtke, K. (2021). Not as "blurred" as expected? Acuity and spatial summation in the pain system. Pain, 162(3), 794.

Rosén, A., Feldreich, A., Dabirian, N., & Ernberg, M. (2008). Effect of heterotopic noxious conditioning stimulation on electrical and pressure pain thresholds in two different anatomical regions. Acta Odontologica Scandinavica, 66(3), 181–188.

Bajaj, P., Arendt-Nielsen, L., & Andersen, O. K. (2005). Facilitation and inhibition of withdrawal reflexes following repetitive stimulation: electro- and psychophysiological evidence for activation of noxious inhibitory controls in humans. European Journal of Pain, 9(1), 25–31.

Yuan, W., Ming, Z., Rana, N., Hai, L., Chen-Wang, J., & Shao-Hui, M. (2010). A functional magnetic resonance imaging study of human brain in pain-related areas induced by electrical stimulation with different intensities. Neurology India, 58(6), 922.

Khoshnejad, M., Piché, M., Saleh, S., Duncan, G., & Rainville, P. (2014). Serial processing in primary and secondary somatosensory cortex: A DCM analysis of human fMRI data in response to innocuous and noxious electrical stimulation. Neuroscience Letters, 577, 83–88.

Filitz, J., Ihmsen, H., Günther, W., Tröster, A., Schwilden, H., Schüttler, J., & Koppert, W. (2008). Supra-additive effects of tramadol and acetaminophen in a human pain model☆. Pain, 136(3), 262–270. doi:10.1016/j.pain.2007.06.036

Ma, Y., Wang, C., Luo, S., Li, B., Wager, T. D., Zhang, W., … Han, S. (2016). Serotonin transporter polymorphism alters citalopram effects on human pain responses to physical pain. NeuroImage, 135, 186–196. doi:10.1016/j.neuroimage.2016.04

Tröster, A., Ihmsen, H., Singler, B., Filitz, J., & Koppert, W. (2012). Interaction of Fentanyl and Buprenorphine in an Experimental Model of Pain and Central Sensitization in Human Volunteers. The Clinical Journal oF Pain, 28(8), 705–711. doi:10.1097/ajp.0b013e318241d948In

Sarton, E., Olofsen, E., Romberg, R., den Hartigh, J., Kest, B., Nieuwenhuijs, D., … & Dahan, A. (2000). Sex differences in morphine analgesia: an experimental study in healthy volunteers. The Journal of the American Society of Anesthesiologists, 93(5), 1245-1254.

Chizh, B. A., Dusch, M., Puthawala, M., Schmelz, M., Cookson, L. M., Martina, R., … Koppert, W. (2004). The Effect of Intravenous Infusion of Adenosine on Electrically Evoked Hyperalgesia in a Healthy Volunteer Model of Central Sensitization. Anesthesia & Analgesia, 99(3), 816–822. doi:10.1213/01.ane.0000132995.35

Kim, T. K., Niklewski, P. J., Martin, J. F., Obara, S., & Egan, T. D. (2015). Enhancing a sedation score to include truly noxious stimulation: the Extended Observer's Assessment of Alertness and Sedation (EOAA/S). BJA: British Journal of Anaesthesia, 115(4), 569-577.

Naef, M., Curatolo, M., Petersen-Felix, S., Arendt-Nielsen, L., Zbinden, A., & Brenneisen, R. (2003). The analgesic effect of oral delta-9-tetrahydrocannabinol (THC), morphine, and a THC-morphine combination in healthy subjects under experimental pain conditions. Pain, 105(1), 79–88. doi:10.1016/s0304-3959(03)00163-5

Liu, S. S., Gerancher, J. C., Bainton, B. G., Kopacz, D. J., & Carpenter, R. L. (1996). The Effects of Electrical Stimulation at Different Frequencies on Perception and Pain in Human Volunteers. Anesthesia & Analgesia, 82(1), 98–102. doi:10.1097/00000539-199601000-00017

Yuan, W., Dan, L., Netra, R., Shaohui, M., Chenwang, J., & Ming, Z. (2013). A pharmaco-fMRI study on pain networks induced by electrical stimulation after sumatriptan injection. Experimental Brain Research, 226(1), 15–24. doi:10.1007/s00221-013-3405-8

Imai, Y., Petersen, K. K., Mørch, C. D., & Arendt Nielsen, L. (2016). Comparing test–retest reliability and magnitude of conditioned pain modulation using different combinations of test and conditioning stimuli. Somatosensory & Motor Research, 33(3-4), 169–177. doi:10.1080/08990220.2016.1229178

Walker, J. S., Arroyo, J. F., Nguyen, T. U. A. N., & Day, R. O. (1993). Analgesic efficacy of non-steroidal anti-inflammatory drugs in experimental pain in humans. British journal of clinical pharmacology, 36(5), 417.

Ravn, P., Frederiksen, R., Skovsen, A. P., Christrup, L. L., & Werner, M. U. (2012). Prediction of pain sensitivity in healthy volunteers. Journal of pain research, 5, 313.

Engskov, A. S., Lejbman, I., &Åkeson, J. (2021). Randomized cross-over evaluation of investigator gender on pain thresholds in healthy volunteers. GMS German Medical Science, 19.

Tiippana, E., Hamunen, K., Kontinen, V., &Kalso, E. (2013). The effect of paracetamol and tropisetron on pain: experimental studies and a review of published data. Basic & clinical pharmacology & toxicology, 112(2), 124-131.

Devoize, L., Dualé, C., Dubray, C., &Dallel, R. (2017). Impact of sympathetic activation on pain threshold in human subjects. Physiology &Behavior, 177, 1–3. doi:10.1016/j.physbeh.2017.04.003

Aasted, C. M., Yücel, M. A., Steele, S. C., Peng, K., Boas, D. A., Becerra, L., &Borsook, D. (2016). Frontal lobe hemodynamic responses to painful stimulation: a potential brain marker of nociception. PLoS One, 11(11), e0165226.

Fossataro, C., Bucchioni, G., D'agata, F., Bruno, V., Morese, R., Krystkowiak, P., &Garbarini, F. (2018). Anxiety-dependent modulation of motor responses to pain expectancy. Social Cognitive and Affective Neuroscience, 13(3), 321-330.

D. Lelic, C. D. Mørch, K. Hennings, O. K. Andersen and A. M. Drewes. (2011). Differences in perception and brain activation following stimulation by large versus small area cutaneous surface electrodes. 10.1002/j.1532-2149.2011.00063.x

Swider, K., Bruña, R., & Moratti, S. . (2024). How to make calibration less painful–a proposition of an automatic, reliable and time-efficient procedure. . 10.1111/psyp.14505

Lea-Carnall, C. A., El-Deredy, W., Stagg, C. J., Williams, S. R., & Trujillo-Barreto, N. J. (2023). A mean-field model of glutamate and GABA synaptic dynamics for functional MRS. 10.1016/j.neuroimage.2022.119813

Stacheneder R, Alt L, Straube A, Ruscheweyh R. (2023). Effects of Transcranial Direct Current Stimulation (t-DCS) of the Cerebellum on Pain Perception and Endogenous Pain Modulation: a Randomized, Monocentric, Double-Blind, Sham-Controlled Crossover Study. 10.1007/s12311-022-01498-x

van Lennep JHPA, van Middendorp H, Veldhuijzen DS, Peerdeman KJ, Blythe JS, Thomaidou MA, Heyman T, Evers AWM. (2023). The optimal learning cocktail for placebo analgesia: a randomized controlled trial comparing individual and combined techniques. 10.1016/j.jpain.2023.07.009

Viudes-Sarrión, N., Aleixandre-Carrera, F., Beltrá, P., Ortega, F. J., Molina-Payá, F. J., Velasco, E., & Delicado-Miralles, M. (2024). Blood flow effects of percutaneous peripheral nerve stimulation. A blinded, randomized clinical trial. 10.1111/eci.14091

Y Bi, X Liu, X Zhao, S Wei, J Li, F Wang, W Luo, L Hu. (2023). Enhancing pain modulation: the efficacy of synchronous combination of virtual reality and transcutaneous electrical nerve stimulation. 10.1136/gpsych-2023-101164

Giuseppe Valerio Aurucci, Greta Preatoni, Arianna Damiani, Stanisa Raspopovic. (2023). Brain-Computer Interface to Deliver Individualized Multisensory Intervention for Neuropathic PAIN. 10.1007/s13311-023-01396-y

Anna M. Zamorano, Boris Kleber, Federico Arguissain, Shellie Boudreau, Peter Vuust, Herta Flor, Thomas Graven-Nielsen. (2023). Extensive Sensorimotor Training Predetermines Central PAIN Changes During the Development of Prolonged Muscle PAIN. 10.1016/j.jPAIN.2023.01.017

Bertram T., Hoffmann Ayala D., Huber M., Brandl F., Starke G., Sorg C., Mulej Bratec S. (2023). Human threat circuits: Threats of pain, aggressive conspecific, and predator elicit distinct BOLD activations in the amygdala and hypothalamus. 10.3389/fpsyt.2022.1063238

Danböck, S. K., Franke, L. K., Miedl, S. F., Liedlgruber, M., Bürkner, P. C., & Wilhelm, F. H. . (2023). Experimental induction of peritraumatic dissociation: The role of negative affect and pain and their psychophysiological and neural correlates. 10.1016/j.brat.2023.104289

de Almeida Paz I., Sonda F.C., Fröhlich M., Durigan J.L.Q., Vaz M.A. (2023). Kilohertz Frequency Alternating Current Induces Less Evoked Torque and Less Neuromuscular Efficiency Than Pulsed Current in Healthy People: A Randomized Crossover Trial. 10.1123/jsr.2022-0159

Delgado-Sanchez, A., Charalambous, C., Trujillo-Barreto, N. J., Safi, H., Jones, A., Sivan, M., .. & Brown, C. (2024). Test-retest reliability of Bayesian estimations of the effects of stimulation, prior information and individual traits on pain perception. 10.1002/ejp.2193

Dou H., Lei Y., Pan Y., Li H., Astikainen P. (2023). Impact of observational and direct learning on fear conditioning generalization in humans. 10.1016/j.pnpbp.2022.110650

Eberhardt F, Enax-Krumova E, Tegenthoff M, Höffken O, Özgül ÖS. (2023). Anodal transcutaneous spinal direct current stimulation influences the amplitude of pain-related evoked potentials in healthy subjects. 10.1038/s41598-023-47408-x

Eleonora Parrotta, Patric Bach, Mauro Gianni Perrucci, Marcello Costantini, Francesca Ferri. (2024). Heart is deceitful above all things: Threat expectancy induces the illusory perception of increased heartrate,Cognition. 10.1016/j.cognition.2024.105719

Jadidi A.F., Jensen W., Zarei A.A., Lontis E.R. (2023). Alteration in Cortical Activity and Perceived Sensation Following Modulated TENS. 10.1109/TNSRE.2023.3236038

Joseph S. Blythe, Alessandra C. Mansueto, Sascha B. Duken, Henk R. Cremers. (2023). The generalization of behavioral control over physical threats to social stressors in humans: A pilot fMRI study. 10.1016/j.pscychresns.2023.111598.

Kazuki Hayashida, Yuki Nishi, Taku Matsukawa, Yuya Nagase, Shu Morioka,. (2024). I am not the cause of this PAIN: An experimental study of the cognitive processes underlying causal attribution in the unpredictable situation whether negative outcomes,. 10.1016/j.concog.2023.103622

Li, X., Yao, J., Lin, X., Chen, S., Jin, R., & Peng, W. (2023). Transcranial random noise stimulation over the left dorsolateral prefrontal cortex attenuates pain expectation and perception. 10.1016/j.clinph.2022.12.009

Martin Löffler, Pia Schneider, Sigrid Schuh-Hofer, Sandra Kamping, Katrin Usai, Rolf-Detlef Treede, Frauke Nees, Herta Flor. (2023). Stress-induced hyperalgesia instead of analgesia in patients with chronic musculoskeletal PAIN. 10.1016/j.ynpai.2022.100110

Narrendar RaviChandran, James Hope, Kean Aw, Andrew McDaid. (2023). Modeling the excitation of nerve axons under transcutaneous STIMULation. 10.1016/j.compbiomed.2023.107463

Ondrej Zika, Judith Appel, Corinna Klinge, Lorika Shkreli, Michael Browning, Katja Wiech, Andrea Reinecke,. (2024). Reduction of aversive learning rates in Pavlovian conditioning by angiotensin II antagonist losartan: a randomised controlled trial,. 10.1016/j.biopsych.2024.01.020

Pinto, E. A., Van Damme, S., Torta, D. M., & Meulders, A. (2023). Modulation of attention to pain by goal-directed action: a somatosensory evoked potentials approach. 10.7717/peerj.16544

Rena Gatzounis, Anouk Gelissen, Daniëlla Theuerzeit, Ann Meulders. (2023). Rewarding Approach Behaviour Attenuates the Return of PAIN-Related Avoidance After Successful Extinction with Response Prevention,. 10.1016/j.jPAIN.2023.12.010

Valentina Jelinčić, Diana M. Torta, Lucas Vanden Bossche, Ilse Van Diest, Andreas von Leupoldt,. (2023). Repeated exposure to aversive sensations differentially affects neural gating and bodily perception. 10.1016/j.brat.2023.104422

Xiaomin Huang, Junxiao Yin, Xinli Liu, Wenwei Tan, Mengting Lao, Xianglong Wang, Sishi Liu, Qiling Ou, Danzhe Tang, Wen Wu. (2023). The overgeneralization of PAIN-related fear in individuals with higher PAIN sensitivity: A behavioral and event-related potential study. 10.1016/j.brainres.2023.148473

Yinhua Zhang, Qian Ye, Hao He, Richu Jin, Weiwei Peng. (2023). Neurocognitive Mechanisms Underlying Attention Bias Towards PAIN: Evidence From a Drift-Diffusion Model and Event-Related Potentials. 10.1016/j.jPAIN.2023.03.003

Zhou L., Xu Y., Song F., Li W., Gao F., Zhu Q., Qian Z. (2023). The effect of TENS on sleep: A pilot study. 10.1016/j.sleep.2023.04.029

Zhuo, S., Zhang, Y., Lin, C., & Peng, W. (2023). Testosterone administration enhances the expectation and perception of painful and non-painful somatosensory stimuli. 10.1016/j.psyneuen.2023.106081

G. Misra, W. E. Wang, D. B. Archer, A. Roy and S. A. Coombes. (2017). Automated classification of pain perception using high-density electroencephalography data. 10.1152/jn.00650.2016

C. J. Falconer and F. W. Mast . (2012). Balancing the mind: Vestibular induced facilitation of egocentric mental transformations . 10.1027/1618-3169/a000161

E. Testani, D. Le Pera, C. Del Percio, R. Miliucci, A. Brancucci, C. Pazzaglia, L. De Armas, C. Babiloni, P. M. Rossini and M. Valeriani. (2015). Cortical inhibition of laser pain and laser-evoked potentials by non-nociceptive somatosensory input. 10.1111/ejn.13035

A. Ul Huque, E. Poliakoff and R. J. Brown . (2017). Effects of learning on somatosensory decision-making and experiences . 10.1037/xge0000364;10.1037/xge0000364.supp(Supplemental)

M. S. Volz, V. Suarez-Contreras, M. E. Mendonca, F. S. Pinheiro, L. B. Merabet and F. Fregni. (2013). Effects of Sensory Behavioral Tasks on Pain Threshold and Cortical Excitability. 10.1371/journal.pone.0052968

M. I. Lambert, P. Marcus, T. Burgess and T. D. Noakes. (2002). Electro-membrane microcurrent therapy reduces signs and symptoms of muscle damage. 10.1097/00005768-200204000-00007

Defrin, R., Pope, G., & Davis, K. D. (2008). Interactions between spatial summation, 2-point discrimination and habituation of heat pain. 10.1016/j.pain.2007.12.015

D. Copot and C. Ionescu. (2019). Models for Nociception Stimulation and Memory Effects in Awake and Aware Healthy Individuals. 10.1109/TBME.2018.2854917

Ditre, J. W., Zale, E. L., LaRowe, L. R., Kosiba, J. D., & De Vita, M. J. (2018). Nicotine deprivation increases pain intensity, neurogenic inflammation, and mechanical hyperalgesia among daily tobacco smokers. 10.1037/abn0000353

C. Ritter, M. N. Hebart, T. Wolbers and U. Bingel. (2014). Representation of spatial information in key areas of the descending pain modulatory system. 10.1523/JNEUROSCI.4342-13.2014

Algoet, M., Duque, J., Iannetti, G. D., & Mouraux, A. (2018). Temporal Profile and Limb-specificity of Phasic Pain-Evoked Changes in Motor Excitability. 10.1016/j.neuroscience.2018.06.039

D. Mantini, M. Caulo, A. Ferretti, G. L. Romani and A. Tartaro. (2009). Noxious somatosensory stimulation affects the default mode of brain function: Evidence from functional MR imaging. 10.1148/radiol.2533090602

Elina Tiippana, Katri Hamunen, Vesa Kontinen, Eija KalsoFirst published: 21 August 2012. (2012). The Effect of Paracetamol and Tropisetron on Pain: Experimental Studies and a Review of Published Data. 10.1111/j.1742-7843.2012.00935.x

L. Devoize, C. Dualé, C. Dubray and R. Dallel. (2017). Impact of sympathetic activation on pain threshold in human subjects. 10.1016/j.physbeh.2017.04.003

E. M. Dölker, S. Lau, M. A. Bernhard and J. Haueisen. (2022). Perception thresholds and qualitative perceptions for electrocutaneous stimulation. 10.1038/s41598-022-10708-9
C. Geber, R. Fondel, H. H. Krämer, R. Rolke, R.-D. Treede, C. Sommer and F. Birklein. (2007). Psychophysics, flare, and neurosecretory function in human pain models: Capsaicin versus electrically evoked pain. 10.1016j.jpain.2007.01.008
Koppert, Wolfgang; Filitz, J√∂rg; Tr√∂ster, Andreas; Ihmsen, Harald; Angst, Martin; Flor, Herta' Sch√tttler, J√trgen; Schmelz, Martin. (2005). Activation of naloxone-sensitive and -insensitive inhibitory systems in a human pain model. 10.1016/j.jpain.2005.07.002
I. R. Pahl, W. Koppert, C. Enk, R. Sittl, S. Mühldorfer, E. G. Hahn, M. Schmelz and D. Schwab. (2003). Different lipid profiles as constituencies of liquid formula diets do not influence pain perception and the efficacy of opioids in a human model of acute pain and hyperalgesia. 10.1016/S0304-3959(03)00094-0
W. Koppert, H. Ihmsen, N. Körber, A. Wehrfritz, R. Sittl, M. Schmelz and J. Schüttler. (2005). Different profiles of buprenorphine-induced analgesia and antihyperalgesia in a human pain model. 10.1016/j.pain.2005.06.030
B. A. Chizh, M. Göhring, A. Tröster, G. K. Quartey, M. Schmelz and W. Koppert. (2007). Effects of oral pregabalin and aprepitant on pain and central sensitization in the electrical hyperalgesia model in human volunteers. 10.1093bja/ael344
T. Schneider, L. Zurbriggen, M. Dieterle, E. Mauermann, P. Frei, K. Mercer-Chalmers-Bender and W.Ruppen. (2022). Pain response to cannabidiol in induced acute nociceptive pain, allodynia, and hyperalgesia by using a model mimicking acute pain in healthy adults in a randomized trial (CANAB I). 10.1097j.pain.0000000000002310
T. Schneider, J. Luethi, E. Mauermann, O. Bandschapp and W. Ruppen. (2020). Pain Response to Open Label Placebo in Induced Acute Pain in Healthy Adult Males. 10.1097/aln.0000000000003076
J. Filitz, H. Ihmsen, W. Günther, A. Tröster, H. Schwilden, J. Schüttler and W. Koppert. (2008). Supra-additive effects of tramadol and acetaminophen in a human pain model. 10.1016/j.pain.2007.06.036
Y. Haller, A. R. Gantenbein, P. Willimann, D. R. Spahn and K. Maurer. (2014). Systemic Ropivacaine Diminishes Pain Sensitization Processes: A Randomized, Double-Blinded, Placebo-Controlled, Crossover Study in Healthy Volunteers. 10.1007/s40122-013-0021-z
A. Chizh, M. Dusch, M. Puthawala, M. Schmelz, L. M. Cookson, R. Martina, J. Brown and W. Koppert. (2004). The effect of intravenous infusion of adenosine on electrically evoked hyperalgesia in a healthy volunteer model of central sensitization. 10.1213/01.Ane.0000132995.35119.99
Mauermann, C. A. Blum, G. Lurati Buse, O. Bandschapp and W. Ruppen. (2017). Time course of copeptin during a model of experimental pain and hyperalgesia: A randomised volunteer crossover trial. 10.1097eja.0000000000000592
Nilsson, H.-J., Psouni, E., & Schouenborg, J. (2003). Long term depression of human nociceptive skin senses induced by thin fibre stimulation. 10.1016/S1090-3801(02)00120-9
T. Eberle, B. Doganci, H. Krämer, M. Fechir, I. Wagner, C. Sommer and F. Birklein. (2010). Mechanical but not painful electrical stimuli trigger TNF alpha release in human skin. 10.1016/j.expneurol.2009.11.008
R. H. Watkins, J. Wessberg, H. B. Wasling, J. P. Dunham, H. Olausson, R. D. Johnson and R.Ackerley. (2017). Optimal delineation of single c-tactile and c-nociceptive afferents in humans by latency slowing. 10.1152/jn.00939.2016
B. van den Berg, H. J. Hijma, I. Koopmans, R. J. Doll, R. G. J. A. Zuiker, G. J. Groeneveld and J. R.Buitenweg. (2022). Simultaneous measurement of intra-epidermal electric detection thresholds and evoked potentials for observation of nociceptive processing following sleep deprivation. 10.1007/s00221-021-06284-5
B. van den Berg, R. J. Doll, A. L. H. Mentink, P. S. Siebenga, G. J. Groeneveld and J. R. Buitenweg. (2020). Simultaneous tracking of psychophysical detection thresholds and evoked potentials to study nociceptive processing. 10.3758/s13428-019-01338-7
J. Tigerholm, T. N. Hoberg, D. Brønnum, M. Vittinghus, K. S. Frahm and C. D. Mørch. (2020). Small and large cutaneous fibers display different excitability properties to slowly increasing ramp pulses. 10.1152/jn.00629.2019
Hayamizu, M., Hagiwara, K., Hironaga, N., Ogata, K., Hoka, S., & Tobimatsu, S. . (2016). A spatiotemporal signature of cortical pain relief by tactile stimulation: An MEG study. 10.1016/j.neuroimage.2016.01.065
L. Vo and P. D. Drummond. (2014). Coexistence of ipsilateral pain-inhibitory and facilitatory processes after high-frequency electrical stimulation. 10.1002/j.1532-2149.2013.00370.x
M. D. Holbert, A. Pedler, D. Camfermann and D. S. Harvie. (2017). Comparison of spatial summation properties at different body sites. 10.1016/j.sjpain.2017.07.017
R. Björkman and M. Elam. (1993). Diclofenac evaluated in a human experimental model of central pain. 10.10160304-3959(93)90209-8
W. Koppert, R. Sittl, K. Scheuber, M. Alsheimer, M. Schmelz and J. Schüttler. (2003). Differential modulation of remifentanil-induced analgesia and postinfusion hyperalgesia by S-ketamine and clonidine in humans. 10.109700000542-200307000-00025
M. Quante, E. Scharein, R. Zimmermann, B. Langer-Brauburger and B. Bromm. (2004). Dissociation of morphine analgesia and sedation evaluated by EEG measures in healthy volunteers. 10.1055/s-0031-1296951
R. J. Doll, A. C. A. Maten, S. P. G. Spaan, P. H. Veltink and J. R. Buitenweg. (2016). Effect of temporal stimulus properties on the nociceptive detection probability using intra-epidermal electrical stimulation. 10.1007s00221-015-4451-1
P. Zheng, Z. Lyu and T. Jackson. (2019). Effects of trait fear of pain on event-related potentials during word cue presentations that signal potential pain. 10.1111/ejn.14495
Y. Zeng, X.-C. Liang, J.-P. Dai, Y. Wang, Z.-L. Yang, M. Li, G.-Y. Huang and J. Shi. (2006). Electroacupuncture modulates cortical activities evoked by noxious somatosensory stimulations in human. 10.1016j.brainres.2006.03.123
A. Wehrfritz, M. Bauer, N. Noel, J. F. Ramirez-Gil, H. Ihmsen, J. Prottengeier, J. Schüttler and B.Bessiere. (2021). Evaluation of antihyperalgesic and analgesic effects of 35% nitrous oxide when combined with remifentanil: A randomised phase 1 trial in volunteers. 10.1097/eja.0000000000001468
J. Kalliomäki, K. Huizar, M. Kågedal, B. Hägglöf and M. Schmelz. (2013). Evaluation of the effects of a metabotropic glutamate receptor 5-antagonist on electrically induced pain and central sensitization in healthy human volunteers. 10.1002/j.1532-2149.2013.00327.x
G. P. N. Michaux, W. Magerl, F. Anton and R. D. Treede. (2012). Experimental characterization of the effects of acute stresslike doses of hydrocortisone in human neurogenic hyperalgesia models. 10.1016/j.pain.2011.10.043
J. B. Manresa, O. K. Andersen, A. Mouraux and E. N. van den Broeke. (2018). High frequency electrical stimulation induces a long-lasting enhancement of event-related potentials but does not change the perception elicited by intra-epidermal electrical stimuli delivered to the area of increased mechanical pinprick sensitivity. 10.1371journal.pone.0203365
K. Takahashi, T. Taguchi, K. Itoh, K. Okada, K. Kawakita and K. Mizumura. (2005). Influence of surface anesthesia on the pressure pain threshold measured with different-sized probes. 10.1111/j.1600-0765.2005.00794.x
A. M. González-Roldán, J. L. Terrasa, M. A. Prats-Sedano, C. Sitges, M. van der Meulen, F. Anton and P. Montoya. (2021). Intact pain modulation through manipulation of controllability and expectations in aging. 10.1002/ejp.1760
A. P. Wehrfritz, H. Ihmsen, S. Schmidt, C. Müller, J. Filitz, J. Schüttler and W. Koppert. (2010). Interaction of physostigmine and alfentanil in a human pain model. 10.1093/bja/aep372
B. Knost, H. Flor, N. Birbaumer and M. M. Schugens. (1999). Learned maintenance of pain: muscle tension reduces central nervous system processing of painful stimulation in chronic and subchronic pain patients. https://pubmed.ncbi.nlm.nih.gov/10554589/
B. Singler, A. Tröster, N. Manering, J. Schüttler and W. Koppert. (2007). Modulation of remifentanil-induced postinfusion hyperalgesia by propofol. 10.1213/01.ane.0000261305.22324.f3
L. F. Chu, T. Cun, L. K. Ngai, J. E. Kim, A. K. Zamora, C. A. Young, M. S. Angst and D. J. Clark. (2012). Modulation of remifentanil-induced postinfusion hyperalgesia by the β-blocker propranolol in humans. 10.1016j.pain.2012.01.014
M. Segerdahl. (2006). Multiple dose gabapentin attenuates cutaneous pain and central sensitisation but not muscle pain in healthy volunteers. 10.1016/j.pain.2006.05.008
L. Leistritz, T. Weiss, K. J. Bär, F. De VicoFallani, F. Babiloni, H. Witte and T. Lehmann. (2013). Network Redundancy Analysis of Effective Brain Networks; a Comparison of Healthy Controls and Patients with Major Depression. 10.1371/journal.pone.0060956
M. Valeriani, P. Tonali, L. De Armas, S. Mariani, F. Vigevano and D. Le Pera. (2008). Nociceptive contribution to the evoked potentials after painful intramuscular electrical stimulation. 10.1016/j.neures.2007.10.008
Diers, C. Koeppe, P. Yilmaz, K. Thieme, J. Markela-Lerenc, M. Schiltenwolf, K. Van Ackern and H.Flor. (2008). Pain ratings and somatosensory evoked responses to repetitive intramuscular and intracutaneous stimulation in fibromyalgia syndrome. 10.1097/WNP.0b013e31817759c5
M. Dieterle, L. Zurbriggen, E. Mauermann, K. Mercer-Chalmers-Bender, P. Frei, W. Ruppen and T. Schneider. (2022). Pain response to cannabidiol in opioid-induced hyperalgesia, acute nociceptive pain, and allodynia using a model mimicking acute pain in healthy adults in a randomized trial (CANAB II). 10.1097j.pain.0000000000002591
A. Ikoma, M. Fartasch, G. Heyer, Y. Miyachi, H. Handwerker and M. Schmelz. (2004). Painful stimuli evoke itch in patients with chronic pruritus: central sensitization for itch. 10.1212/wnl.62.2.212
S. Omori, S. Isose, S. Misawa, K. Watanabe, Y. Sekiguchi, K. Shibuya, M. Beppu, H. Amino and S.Kuwabara. (2017). Pain-related evoked potentials after intraepidermal electrical stimulation to Aδ and C fibers in patients with neuropathic pain. 10.1016/j.neures.2017.03.007
K. Inui, T. D. Tran, M. Hoshiyama and R. Kakigi. (2002). Preferential stimulation of Aδ fibers by intra-epidermal needle electrode in humans. 10.1016/S0304-3959(01)00453-5
B. Geng, J. Dong, W. Jensen, S. Dosen, D. Farina and E. N. Kamavuako. (2018). Psychophysical evaluation of subdermal electrical stimulation in relation to prosthesis sensory feedback. 10.1109/TNSRE.2018.2803844
H. E. Torebjörk, J. L. Ochoa and W. Schady. (1984). Referred pain from intraneural stimulation of muscle fascicles in the median nerve. 10.1016/0304-3959(84)90882-0
A. Yoshino, Y. Okamoto, K. Onoda, S. Yoshimura, Y. Kunisato, Y. Demoto, G. Okada and S.Yamawaki. (2010). Sadness enhances the experience of pain via neural activation in the anterior cingulate cortex and amygdala: An fMRI study. 10.1016/j.neuroimage.2009.11.079
D. D. Price, J. Mao, H. Frenk and D. J. Mayer. (1994). The N-methyl-D-aspartate receptor antagonist dextromethorphan selectively reduces temporal summation of second pain in man. 10.10160304-3959(94)90069-8
O. Bandschapp, J. Filitz, A. Urwyler, W. Koppert and W. Ruppen. (2011). Tropisetron blocks analgesic action of acetaminophen: a human pain model study. 10.1016/j.pain.2011.02.003
Richter, M., Schroeter, C., Puensch, T., Straube, T., Hecht, H., Ritter, A., Miltner, W. H., & Weiss, T. (2014). Pain-related and negative semantic priming enhances perceived pain intensity. 10.1155/2014/425321
S. Petersen-Felix, L. Arendt-Nielsen, P. Bak, M. Fischer and A. M. Zbinden. (1996). Psychophysical and electrophysiological responses to experimental pain may be influenced by sedation: comparison of the effects of a hypnotic (propofol) and an analgesic (alfentanil). 10.1093/bja/77.2.165
H. Vossen, G. Van Breukelen, H. Hermens, J. Van Os and R. Lousberg. (2011). More potential in statistical analyses of event-related potentials: A mixed regression approach. 10.1002/mpr.348
B. Bromm, W. Meier and E. Scharein. (1989). Pre-stimulus/post-stimulus relations in EEG spectra and their modulations by an opioid and an antidepressant. 10.1016/0013-4694(89)90119-3
J. Brennum and T. S. Jensen. (1992). Relationship between vertex potentials and magnitude of pre-pain and pain sensations evoked by electrical skin stimuli. 10.1016/0013-4694(92)90008-6
E. Mauermann, J. Filitz, P. Dolder, K. M. Rentsch, O. Bandschapp and W. Ruppen. (2016). Does Fentanyl Lead to Opioid-induced Hyperalgesia in Healthy Volunteers?: A Double-blind, Randomized, Crossover Trial. 10.1097aln.0000000000000976
H. Lenz, J. Raeder, T. Draegni, F. Heyerdahl, M. Schmelz and A. Stubhaug. (2011). Effects of COX inhibition on experimental pain and hyperalgesia during and after remifentanil infusion in humans. 10.1016j.pain.2011.02.007
A. Tröster, H. Ihmsen, B. Singler, J. Filitz and W. Koppert. (2012). Interaction of fentanyl and buprenorphine in an experimental model of pain and central sensitization in human volunteers. 10.1097/AJP.0b013e318241d948
A. Wehrfritz, S. Schaefer, A. Troester, N. Noel, B. Bessiere, G. Apiou-Sbirlea, G. Simonnet, J. Schuettler and P. Richebé. (2016). A randomized phase I trial evaluating the effects of inhaled 50-50% N2 O-O2 on remifentanil-induced hyperalgesia and allodynia in human volunteers. 10.1002/ejp.870
O. Bandschapp, J. Filitz, H. Ihmsen, A. Berset, A. Urwyler, W. Koppert and W. Ruppen. (2010). Analgesic and antihyperalgesic properties of propofol in a human pain model. 10.1097/ALN.0b013e3181e33ac8
L. Vo and P. D. Drummond. (2017). Effect of combined opioid receptor and α2-adrenoceptor blockade on anxiety and electrically evoked startle responses. 10.1177/0269881116689259
L. F. Chu, J. Dairmont, A. K. Zamora, C. A. Young and M. S. Angst. (2011). The endogenous opioid system is not involved in modulation of opioid-induced hyperalgesia. 10.1016/j.jpain.2010.05.006
L. Vo and P. D. Drummond. (2016). Involvement of α2-adrenoceptors in inhibitory and facilitatory pain modulation processes. 10.1002/ejp.736
S. S. Robertson, M. S. Mouksassi and F. Varin. (2019). Population Pharmacokinetic/Pharmacodynamic Modeling of O-Desmethyltramadol in Young and Elderly Healthy Volunteers. 10.1007/s40266-019-00681-w
A. Rosen, A. Feldreich, N. Dabirian and M. Ernberg. (2008). Effect of heterotopic noxious conditioning stimulation on electrical and pressure pain thresholds in two different anatomical regions. 10.1080/00016350802169111
M. Nordin and J. Fagius. (1995). Effect of noxious stimulation on sympathetic vasoconstrictor outflow to human muscles. 10.1113/jphysiol.1995.sp021101
S. Skinner-Robertson, M. S. Mouksassi and F. Varin. (2018). Evaluation of an Experimental Pain Model by Noncompartmental Analysis of Results from a Randomized Placebo Controlled Trial. https://pubmed.ncbi.nlm.nih.gov/30045593/
M. Roosink, J. R. Buitenweg, G. J. Renzenbrink, A. C. H. Geurts and M. J. Ijzerman. (2011). Altered cortical somatosensory processing in chronic stroke: A relationship with post-stroke shoulder pain. 10.3233/NRE-2011-0661
M. Amand, F. Nguyen-Huu and C. Balestra. (2011). Acupuncture effect on thermal tolerance and electrical pain threshold: a randomised controlled trial. 10.1136/aim.2010.002485
C. Marson, S. Schneider, F. Meys and R. Wennig. (2001). Cutaneomotor integration in human hand motor areas: Somatotopic effect and interaction of afferents. 10.1007/s002210100859
O. Sahin, S. Yildiz and N. Yildiz. (2011). Cutaneous silent period in fibromyalgia. 10.1179016164110x12767786356516
A. V. Syrovegin, M. L. Kukushkin, A. V. Gnezdilov, A. M. Ovechkin and T. S. Li. (2000). EMG responses in humans during painful heterosegmentary stimulation. https://pubmed.ncbi.nlm.nih.gov/11182818/
P. J. Broser and C. Braun. (2012). Hydraulic driven fast and precise nonmagnetic tactile stimulator for neurophysiological and MEG measurements. 10.1109/TBME.2012.2212191
M. Kofler, F. X. Glocker, A. A. Leis, C. Seifert, J. Wissel, M. F. Kronenberg and P. Fuhr. (1998). Modulation of upper extremity motoneurone excitability following noxious finger tip stimulation in man: a study with transcranial magnetic stimulation. 10.1016/s0304-3940(98)00243-2
J. M. Pimentel, R. Petrillo, M. M. F. Vieira, L. M. Pereira Giuliano, C. J. Tierra-Criollo, N. I. O. De Braga, J. A. MacielNóbrega and G. M. Manzano. (2006). Perceptions and electric senoidal current stimulation. 10.1590S0004-282X2006000100003
B. A. Coda, M. C. Brown, R. Schaffer, G. Donaldson, R. Jacobson, B. Hautman and D. D. Shen. (1994). Pharmacology of epidural fentanyl, alfentanil, and sufentanil in volunteers. 10.1097/00000542-199411000-00008
E. P. V. Félix, L. M. P. Giuliano, C. J. Tierra-Criollo, G. Gronich, N. I. O. Braga, C. A. Peres, J. A. M. Nóbrega and G. M. Manzano. (2009). Sensations and reaction times evoked by electrical sinusoidal stimulation. 10.1016j.neucli.2009.10.001
C. Christmann, M. Ruf, D. F. Braus and H. Flor. (2002). Simultaneous electroencephalography and functional magnetic resonance imaging of primary and secondary somatosensory cortex in humans after electrical stimulation. 10.1016/S0304-3940(02)00969-2
Brown, Christopher A.; Jones, Anthony K. P. (2008). A role for midcingulate cortex in the interruptive effects of pain anticipation on attention. 10.1016/j.clinph.2008.06.014
D. De Massari, T. Matuz, A. Furdea, C. A. Ruf, S. Halder and N. Birbaumer. (2013). Brain-computer interface and semantic classical conditioning of communication in paralysis. 10.1016/j.biopsycho.2012.10.015
Horland, Allan A.; Wolff, B. Berthold. (1973). Changes in descending pain threshold related to rate of noxious stimulation. 10.1037/h0033868
Sharar, Sam R.; Alamdari, Ava; Hoffer, Christine; Hoffman, Hunter G.; Jensen, Mark P.; Patterson, David R. (2016). Circumplex model of affect: A measure of pleasure and arousal during virtual reality distraction analgesia. 10.1089/g4h.2015.0046
Gustorff, K. H. Hoerauf, P. Lierz and H. G. Kress. (2003). Comparison of different quantitative sensory testing methods during remifentanil infusion in volunteers. 10.1093/bja/aeg161
A. Dawson and T. List. (2009). Comparison of pain thresholds and pain tolerance levels between Middle Easterners and Swedes and between genders. 10.1111/j.1365-2842.2009.01943.x
C. Babiloni, L. Arendt-Nielsen, R. D. Pascual-Marqui, P. M. Rossini, A. Brancucci, C. Del Percio, F. Babiloni, G.Sabbatini and A. C. N. Chen. (2004). Cortical sensorimotor interactions during the expectancy of a Go/No-Go task: Effects of painful stimuli. 10.1037/0735-7044.118.5.925
M. Wicking, F. Steiger, F. Nees, S. J. Diener, O. Grimm, M. Ruttorf, L. R. Schad, T. Winkelmann, G. Wirtz and H.Flor. (2016). Deficient fear extinction memory in posttraumatic stress disorder. 10.1016/j.nlm.2016.09.016
M. E. Berman, J. R. Fanning, C. R. Guillot, A. Marsic, J. Bullock, M. R. Nadorff and M. S. McCloskey . (2017). Effect of alcohol dose on deliberate self-harm in men and women . 10.1037/ccp0000222
A. Doehring, N. Küsener, K. Flühr, T. J. Neddermeyer, G. Schneider and J. Lötsch. (2011). Effect sizes in experimental pain produced by gender, genetic variants and sensitization procedures. 10.1371/journal.pone.0017724
F. Pujia, M. Serrao, M. Brienza, E. Vestrini, G. O. Valente, G. Coppola and F. Pierelli. (2014). Effects of a selective serotonin reuptake inhibitor escitalopram on the cutaneous silent period: a randomized controlled study in healthy volunteers. 10.1016/j.neulet.2014.02.036
H. Yamasaki, R. Kakigi, S. Watanabe and M. Hoshiyama. (2000). Effects of distraction on pain-related somatosensory evoked magnetic fields and potentials following painful electrical stimulation. 10.1016S0926-6410(99)00056-7
Warbrick, D. Sheffield and A. Nouwen. (2006). Effects of pain-related anxiety on components of the pain event-related potential. 10.1111/j.1469-8986.2006.00425.x
Mueller, D., Obermann, M., Koeppen, S., Kavuk, I., Yoon, M. S., Sack, F., Diener, H. C., Kaube, H., & Katsarava, Z. . (2010). Electrically evoked nociceptive potentials for early detection of diabetic small-fiber neuropathy. 10.1111/j.1468-1331.2009.02938.x
L. M. May, P. Kosek, F. Zeidan and E. T. Berkman. (2018). Enhancement of Meditation Analgesia by Opioid Antagonist in Experienced Meditators. 10.1097/PSY.0000000000000580
B. A. Coda, M. C. Brown, L. Risler, K. Syrjala and D. D. Shen. (1999). Equivalent analgesia and side effects during epidural and pharmacokinetically tailored intravenous infusion with matching plasma alfentanil concentration. 10.1097/00000542-199901000-00015
I. Lund, T. Lundeberg, J. Kowalski, L. Sandberg, C. N. Budh and E. Svensson. (2005). Evaluation of variations in sensory and pain threshold assessments by electrocutaneous stimulation. 10.1080/09593980590922307
M. Kofler. (2003). Functional organization of exteroceptive inhibition following nociceptive electrical fingertip stimulation in humans. 10.1016/s1388-2457(03)00060-9
P. R. Giancola and D. J. Parrott. (2008). Further evidence for the validity of the Taylor Aggression Paradigm. 10.1002/ab.20235
R. J. Milne, N. E. Kay and R. J. Irwin. (1991). Habituation to repeated painful and non-painful cutaneous stimuli: a quantitative psychophysical study. 10.1007/bf00231861
M. Sanchez, M. Delpont, M. Bachy, R. Kabbaj, D. Annequin and R. Vialle. (2015). How can surgeonfish help pediatric surgeons? A pilot study investigating the antinociceptive effect of fish aquariums in adult volunteers. 10.1155/2015/419412
K. M. Vogt, C. J. Becker, A. D. Wasan and J. W. Ibinson. (2016). Human Posterior Insula Functional Connectivity Differs between Electrical Pain and the Resting State. 10.1089/brain.2016.0436
T. Jochum, M. K. Boettger, C. Burkhardt, G. Juckel and K. J. Bär. (2010). Increased pain sensitivity in alcohol withdrawal syndrome. 10.1016/j.ejpain.2009.11.008
C. Klemenz, M. Regard, P. Brugger and O. Emch. (2009). Laterality of pain: Modulation by placebo and participants' paranormal belief. 10.1097/WNN.0b013e3181b27aeb
R. Don, F. Pierelli, A. Ranavolo, M. Serrao, M. Mangone, M. Paoloni, A. Cacchio, G. Sandrini and V.Santilli. (2008). Modulation of spinal inhibitory reflex responses to cutaneous nociceptive stimuli during upper limb movement. 10.1111/j.1460-9568.2008.06330.x
G. W. Donaldson, C. R. Chapman, Y. Nakamura, D. H. Bradshaw, R. C. Jacobson and C. N. Chapman. (2003). Pain and the defense response: Structural equation modeling reveals a coordinated psychophysiological response to increasing painful stimulation. 10.1016/s0304-3959(02)00351-2
F. H. S. M. Welman, A. E. Smit, J. L. M. Jongen, D. Tibboel, J. N. van der Geest and J. C. Holstege. (2018). Pain experience is somatotopically organized and overlaps with pain anticipation in the human cerebellum. 10.1007s12311-018-0930-9
V. De Pascalis, V. Varriale and I. Cacace. (2015). Pain modulation in waking and hypnosis in women: Event-related potentials and sources of cortical activity. 10.1371/journal.pone.0128474
K. J. Bär, S. Brehm, M. K. Boettger, S. Boettger, G. Wagner and H. Sauer. (2005). Pain perception in major depression depends on pain modality. 10.1016/j.pain.2005.05.016
V. De Pascalis, I. Cacace and F. Massicolle. (2004). Perception and modulation of pain in waking and hypnosis: Functional significance of phase-ordered gamma oscillations. 10.1016/j.pain.2004.07.003
M. Riello, M. P. Cecchini, A. Zanini, M. Di Chiappari, M. Tinazzi and M. Fiorio. (2019). Perception of phasic pain is modulated by smell and taste. 10.1002/ejp.1453
B. Blitz, A. J. Dinnerstein and M. Lowenthal. (1968). Performance on the Pain Apperception Test and tolerance for experimental pain: A lack of relationship. 10.10021097-4679(196801)24:1<73::AID-JCLP2270240121>3.0.CO;2-5
I. Tegeder, S. Meier, M. Burian, H. Schmidt, G. Geisslinger and J. Lötsch. (2003). Peripheral opioid analgesia in experimental human pain models. 10.1093/brain/awg115
P. Ravn, D. J. Foster, M. Kreilgaard, L. Christrup, M. U. Werner, E. L. Secher, U. Skram and R.Upton. (2014). Pharmacokinetic-pharmacodynamic modelling of the analgesic and antihyperalgesic effects of morphine after intravenous infusion in human volunteers. 10.1111/bcpt.12213
C. R. Chapman, S. Oka, D. H. Bradshaw, R. C. Jacobson and G. W. Donaldson. (1999). Phasic pupil dilation response to noxious stimulation in normal volunteers: relationship to brain evoked potentials and pain report. 10.1017s0048577299970373
S. Oka, C. R. Chapman and R. C. Jacobson . (2000). Phasic pupil dilation response to noxious stimulation: Effects of conduction distance, sex, and age . 10.1027//0269-8803.14.2.97
S. Oka, C. R. Chapman, B. Kim, O. Shimizu, N. Noma, O. Takeichi, Y. Imamura and Y. Oi. (2010). Predictability of Painful Stimulation Modulates Subjective and Physiological Responses. 10.1016/j.jpain.2009.07.009
S. Oka, C. R. Chapman, B. Kim, I. Nakajima, O. Shimizu and Y. Oi. (2007). Pupil dilation response to noxious stimulation: Effect of varying nitrous oxide concentration. 10.1016/j.clinph.2007.04.023
F. Beissner, A. Brandau, C. Henke, L. Felden, U. Baumgärtner, R. D. Treede, B. G. Oertel and J. Lötsch. (2010). Quick discrimination of Adelta and C fiber mediated pain based on three verbal descriptors. 10.1371journal.pone.0012944
G. Xie, M. Piché, M. Khoshnejad, V. Perlbarg, J. I. Chen, R. D. Hoge, H. Benali, S. Rossignol, P. Rainville and J. Cohen-Adad. (2012). Reduction of physiological noise with independent component analysis improves the detection of nociceptive responses with fMRI of the human spinal cord. 10.1016/j.neuroimage.2012.06.057
L. Wang, L. Arendt-Nielsen and A. C. Chen. (2004). Scalp field potentials of human pain: spatial effects and temporal relation in finger stimulation. 10.1007/s10548-004-1006-3
B. Gustorff, G. Nahlik, K. H. Hoerauf and H. G. Kress. (2002). The absence of acute tolerance during remifentanil infusion in volunteers. 10.1097/00000539-200205000-00032
H. Kumru, E. Opisso, J. Valls-Solé and M. Kofler. (2009). The effect of a prepulse stimulus on the EMG rebound following the cutaneous silent period. 10.1113/jphysiol.2008.164186
P. R. Giancola, E. L. Helton, A. B. Osborne, M. K. Terry, A. M. Fuss and J. A. Westerfield. (2002). The effects of alcohol and provocation on aggressive behavior in men and women. https://pubmed.ncbi.nlm.nih.gov/11925061/
M. E. Berman, G. D. Jones and M. S. McCloskey. (2005). The effects of diazepam on human self-aggressive behavior. 10.1007/s00213-004-1966-8
BG Oertel, A Schneider, M Rohrbacher, H Schmidt, I Tegeder, G Geisslinger, J Lötsch. (2006). The Partial 5-Hydroxytryptamine1A Receptor Agonist Buspirone does not Antagonize Morphine-induced Respiratory Depression in Humans. 10.1038/sj.clpt.6100018
B. G. Oertel, R. Schmidt, A. Schneider, G. Geisslinger and J. Lötsch. (2006). The μ-opioid receptor gene polymorphism 118A>G depletes alfentanil-induced analgesia and protects against respiratory depression in homozygous carriers. 10.1097/01.fpc.0000220566.90466.a2
Xiaoyun Li a, Junjie Yao a, Xinxin Lin a, Shengxiong Chen b, Richu Jin c, Weiwei Peng a. (2023). Transcranial random noise stimulation over the left dorsolateral prefrontal cortex attenuates pain expectation and perception. 10.1016/j.clinph.2022.12.009
E. W. Howland, R. T. Wakai, B. A. Mjaanes, J. P. Balog and C. S. Cleeland. (1995). Whole head mapping of magnetic fields following painful electric finger shock. 10.1016/0926-6410(95)90005-5
P. M. Rossini, G. Deuschl, V. Pizzella, F. Tecchio, A. Pasquarelli, E. Feifel, G. L. Romani and C. H. Lücking. (1996). Topography and sources of electromagnetic cerebral responses to electrical and air-puff stimulation of the hand. 10.1016/0168-5597(95)00275-8
F. Blankenburg, J. Ruben, R. Meyer, J. Schwiemann and A. Villringer. (2003). Evidence for a rostral-to-caudal somatotopic organization in human primary somatosensory cortex with mirror-reversal in areas 3b and 1. 10.1093cercor/13.9.987
B. Isak, H. Tankisi, K. Pugdahl, L. Ventzel, N. B. Finnerup and A. Fuglsang-Frederiksen. (2021). Neurophysiologic assessment of small fibre damage in chemotherapy-induced peripheral neuropathy. 10.1016j.clinph.2021.02.406
B. A. Coda, M. C. Brown, R. L. Schaffer, G. Donaldson and D. D. Shen. (1995). A pharmacokinetic approach to resolving spinal and systemic contributions to epidural alfentanil analgesia and side-effects. 10.1016/0304-3959(95)00008-G
W. Meissner, T. Weiss, R. H. Trippe, H. Hecht, C. Krapp and W. H. Miltner. (2004). Acupuncture decreases somatosensory evoked potential amplitudes to noxious stimuli in anesthetized volunteers. 10.121301.Ane.0000096191.07929.44
H. G. M. Vossen, G. J. P. van Breukelen, J. van Os, H. J. Hermens and R. Lousberg . (2011). Association between event-related potentials and pain ratings: Not as straightforward as often thought . 10.1027/0269-8803/a000028
M. Hauck, J. Lorenz and A. K. Engel. (2007). Attention to painful stimulation enhances γ-band activity and synchronization in human sensorimotor cortex. 10.1523/JNEUROSCI.2283-07.2007
H. G. Vossen, J. van Os, H. Hermens and R. Lousberg. (2006). Evidence that trait-anxiety and trait-depression differentially moderate cortical processing of pain. 10.1097/01.ajp.0000210913.95664.1a
F. Sartucci, L. Bonfiglio, C. Del Seppia, P. Luschi, S. Ghione, L. Murri and F. Papi. (1997). Changes in pain perception and pain-related somatosensory evoked potentials in humans produced by exposure to oscillating magnetic fields. 10.1016/S0006-8993(97)00755-5
C. Del Percio, A. I. Triggiani, N. Marzano, M. De Rosas, A. Valenzano, A. Petito, A. Bellomo, A. Soricelli, G. Cibelli and C. Babiloni. (2013). Subjects' hypnotizability level affects somatosensory evoked potentials to non-painful and painful stimuli. 10.1016/j.clinph.2013.02.008
Koyama, K. Nakagawa and S. Tanaka. (2017). Transcranial direct current stimulation over the opercular somatosensory region does not influence experimentally induced pain: A triple blind, sham-controlled study. 10.1097/WNR.0000000000000723
M. Hauck, P. Bischoff, G. Schmidt, R. Zimmermann, J. Lorenz, T. J. Morrow and B. Bromm. (2006). Clonidine effects on pain evoked SII activity in humans. 10.1016/j.ejpain.2005.12.003
L. Leistritz, T. Weiss, J. Ionov, K. J. Bär, W. H. R. Miltner and H. Witte. (2010). Connectivity analysis of somato-sensory evoked potentials in patients with major depression. 10.3414/ME09-02-0038
C. Babiloni, A. Brancucci, V. Pizzella, G. L. Romani, F. Tecchio, K. Torquati, F. Zappasodi, L. Arendt-Nielsen, A. C. N. Chen and P. M. Rossini . (2005). Contingent Negative Variation in the Parasylvian Cortex Increases During the Expectancy of Painful Sensorimotor Events: A Magnetoencephalographic Study. 10.1037/0735-7044.119.2.491
Babiloni, C., Capotosto, P., Brancucci, A., Del Percio, C., Petrini, L., Buttiglione, M., Cibelli, G., Romani, G. L., Rossini, P. M., & Arendt-Nielsen, L. (2008). Cortical alpha rhythms are related to the anticipation of sensorimotor interaction between painful stimuli and movements: a high-resolution EEG study. 10.1016/j.pain.2008.05.007
C. J. Vossen, H. G. M. Vossen, E. A. Joosten, J. Van Os and R. Lousberg. (2015). Does habituation differ in chronic low back pain subjects compared to pain-free controls? A cross-sectional pain rating ERP study reanalyzed with the ERFIA multilevel method. 10.1097/MD.0000000000000865
B. Bromm, W. Forth, E. Richter and E. Scharein. (1992). Effects of acetaminophen and antipyrine on non-inflammatory pain and EEG activity. 10.1016/0304-3959(92)90165-8
C. Babiloni, A. Brancucci, P. Capotosto, L. Arendt-Nielsen, A. C. N. Chen and P. M. Rossini . (2005). Expectancy of Pain Is Influenced by Motor Preparation: A High-Resolution EEG Study of Cortical Alpha Rhythms . 10.10370735-7044.119.2.503
H. Flor, C. Braun, T. Elbert and N. Birbaumer. (1997). Extensive reorganization of primary somatosensory cortex in chronic back pain patients. 10.1016/S0304-3940(97)13441-3
J. Vossen, H. G. Vossen, M. A. Marcus, J. van Os and R. Lousberg. (2013). Introducing the event related fixed interval area (ERFIA) multilevel technique: a method to analyze the complete epoch of event-related potentials at single trial level. 10.1371/journal.pone.0079905
E. T. Erdoğan, Z. Küçük, G. Eskikurt, A. Kurt, N. Ermutlu and S. Karamürsel . (2022). Single session anodal transcranial direct current stimulation on different cortical areas: Effects on pain modulation in healthy subjects . 10.1027/0269-8803/a000311
W. Miltner, R. Johnson, C. Braun and W. Larbig. (1989). Somatosensory event-related potentials to painful and non-painful stimuli: Effects of attention. 10.1016/0304-3959(89)90217-0
Andrew C.N. Chen, Rolf-Detlef Treede, Burkhart Bromm. (1985). Tonic pain inhibits phasic pain: Evoked cerebral potential correlates in man. 10.1016/0165-1781(85)90102-7
Klinger, R., Matter, N., Kothe, R., Dahme, B., Hofmann, U. G., & Krug, F. (2010). Unconditioned and conditioned muscular responses in patients with chronic back pain and chronic tension-type headaches and in healthy controls. 10.1016/j.pain.2010.03.036
Chapman, C. Richard; Bradshaw, David H.; Donaldson, Gary W.; Jacobson, Robert C.; Nakamura, Yoshio. (2014). Central noradrenergic mechanisms and the acute stress response during painful stimulation. 10.1177/0269881114543718
D. Roth, S. Petersen-Felix, P. Bak, L. Arendt-Nielsen, M. Fischer, P. Bjerring and A. M. Zbinden. (1996). Analgesic effect in humans of subanaesthetic isoflurane concentrations evaluated by evoked potentials. 10.1093/bja76.1.38
E. Kochs, E. Scharein, O. Möllenberg, B. Bromm and J. Schulte am Esch. (1996). Analgesic efficacy of low-dose ketamine: Somatosensory-evoked responses in relation to subjective pain ratings. 10.109700000542-199608000-00012
Diesch, Eugen; Flor, Herta. (2007). Alteration in the response properties of primary somatosensory cortex related to differential aversive Pavlovian conditioning. 10.1016/j.pain.2007.01.016
F. Pierelli, E. Iacovelli, M. Bracaglia, M. Serrao and G. Coppola. (2013). Abnormal sensorimotor plasticity in migraine without aura patients. 10.1016/j.pain.2013.05.023
L. Vo, S. Hood and P. D. Drummond. (2016). Involvement of Opioid Receptors and α(2)-Adrenoceptors in Inhibitory Pain Modulation Processes: A Double-Blind Placebo-Controlled Crossover Study. 10.1016/j.jpain.2016.07.004
B. van den Berg and J. R. Buitenweg. (2021). Observation of nociceptive processing: Effect of intra-epidermal electric stimulus properties on detection probability and evoked potentials. 10.1007/s10548-020-00816-y
M. J. van Esdonk, I. Lindeman, P. Okkerse, M. L. de Kam, G. J. Groeneveld and J. Stevens. (2018). Population Pharmacokinetic/Pharmacodynamic Analysis of Nociceptive Pain Models Following an Oral Pregabalin Dose Administration to Healthy Subjects. 10.1002/psp4.12318
K. Bromm, W. M. Herrmann and H. Schulz. (1995). Do the B-vitamins exhibit antinociceptive efficacy in men? Results of a placebo-controlled repeated-measures double-blind study. 10.1159/000119186

Snijders, T. J.; Ramsey, N. F.; Koerselman, F.; van Gijn, J. (2009). Attentional modulation fails to attenuate the subjective pain experience in chronic, unexplained pain. 10.1016/j.ejpain.2009.05.019

C. Staahl, R. Upton, D. J. Foster, L. L. Christrup, K. Kristensen, S. H. Hansen, L. Arendt-Nielsen and A. M.Drewes. (2008). Pharmacokinetic-pharmacodynamic modeling of morphine and oxycodone concentrations and analgesic effect in a multimodal experimental pain model. 10.1177/0091270008314465

O. Vassend and S. Knardahl. (2004). Cardiovascular responsiveness to brief cognitive challenges and pain sensitivity in women. 10.1016/j.ejpain.2003.10.006

J. Meincke, M. Hewitt, M. Reischl, R. Rupp, C. Schmidt-Samoa and D. Liebetanz. (2018). Cortical representation of auricular muscles in humans: A robot-controlled TMS mapping and fMRI study. 10.1371/journal.pone.0201277

M. C. Kahya, Ş. Utku Yavuz and K. S. Türker. (2010). Cutaneous silent period in human FDI motor units. 10.1007s00221-010-2380-6

C. H. Wilder-Smith, X. Li, L. Shen, Y. Cao, K. Y. Ho and R. K. Wong. (2014). Dysfunctional endogenous pain modulation in patients with functional dyspepsia. 10.1111/nmo.12291

A. Mailis, P. Plapler, P. Ashby, R. Shoichet and S. Roe. (1997). Effect of intravenous sodium amytal on cutaneous limb temperatures and sympathetic skin responses in normal subjects and pain patients with and without Complex Regional Pain Syndromes (type I and II). I. 10.1016/s0304-3959(96)03301-5

B. Olausson, E. Eriksson, L. Ellmarker, B. Rydenhag, B. C. Shyu and S. A. Andersson. (1986). Effects of naloxone on dental pain threshold following muscle exercise and low frequency transcutaneous nerve stimulation: a comparative study in man. 10.1111/j.1748-1716.1986.tb07818.x

S. Bashir, D. Aisha, A. Hamza, F. Al-Hussain and W. K. Yoo. (2021). Effects of transcranial direct current stimulation on cortex modulation by stimulation of the primary motor cortex and parietal cortex in humans. 10.108000207454.2020.1775594

T. S. Campbell and B. Ditto. (2002). Exaggeration of blood pressure-related hypoalgesia and reduction of blood pressure with low frequency transcutaneous electrical nerve stimulation. 10.1017/S0048577202000847

Ali Mohammadi; Mohammadhossein Ebrahimi; Siru Kaartinen; Gustaf Järnefelt; Jari Karhu; Petro Julkunen. (2018). Individual Characterization of Fast Intracortical Facilitation With Paired Biphasic-Wave Transcranial Magnetic Stimulation. 10.1109/TNSRE.2018.2864311

A. C. N. Chen, F. J. Liu, L. Wang and L. Arendt-Nielsen. (2006). Mode and site of acupuncture modulation in the human brain: 3D (124-ch) EEG power spectrum mapping and source imaging. 10.1016j.neuroimage.2005.08.066

A. Naro, A. Leo, P. Bramanti and R. S. Calabrò. (2015). Moving Toward Conscious Pain Processing Detection in Chronic Disorders of Consciousness: Anterior Cingulate Cortex Neuromodulation. 10.1016/j.jpain.2015.06.014

K. Yamashiro, K. Inui, N. Otsuru, T. Urakawa and R. Kakigi. (2011). Temporal window of integration in the somatosensory modality: An MEG study. 10.1016/j.clinph.2011.03.028

M. de Tommaso, R. Santostasi, V. Devitofrancesco, G. Franco, E. Vecchio, M. Delussi, P. Livrea and Z. Katzarava. (2011). A comparative study of cortical responses evoked by transcutaneous electrical vs CO 2 laser stimulation. 10.1016/j.clinph.2011.05.006

M. Gavaret, S. S. Ayache, V. Mylius, A. Mhalla, M. A. Chalah and J. P. Lefaucheur. (2018). A reappraisal of pain-paired associative stimulation suggesting motor inhibition at spinal level. 10.1016/j.neucli.2018.04.003

H. Hartmann, M. Rütgen, F. Riva and C. Lamm. (2021). Another's pain in my brain: No evidence that placebo analgesia affects the sensory-discriminative component in empathy for pain. 10.1016j.neuroimage.2020.117397

E. M. Bauch and N. Bunzeck. (2015). Anticipation of electric shocks modulates low beta power and event-related fields during memory encoding. 10.1016/j.nlm.2015.06.010

B. Colagiuri and V. F. Quinn. (2018). Autonomic Arousal as a Mechanism of the Persistence of Nocebo Hyperalgesia. 10.1016/j.jpain.2017.12.006

D. M. Niddam, L. Arendt-Nielsen and A. C. N. Chen. (2000). Cerebral dynamics of SEPs to non-painful and painful cutaneous electrical stimulation of the thenar and hypothenar. 10.1023/A:1026655001804

K. Wiech, F. Eippert, J. Vandekerckhove, J. Zaman, K. Placek, F. Tuerlinckx, J. W. S. Vlaeyen and I. Tracey. (2022). Cortico-Brainstem Mechanisms of Biased Perceptual Decision-Making in the Context of Pain. 10.1016/j.jpain.2021.11.006

A. Romaniello, A. Truini, F. Galeotti, C. De Lena, J. C. Willer and G. Cruccu. (2004). Cutaneous silent period in hand muscle is evoked by laser stimulation of the palm, but not the hand dorsum. 10.1002/mus.20040

S. Rottmann, K. Jung and J. Ellrich. (2010). Electrical low-frequency stimulation induces long-term depression of sensory and affective components of pain in healthy man. 10.1016/j.ejpain.2009.06.001

K. Schmidt, K. Forkmann, S. Elsenbruch and U. Bingel. (2020). Enhanced pain-related conditioning for face compared to hand pain. 10.1371/journal.pone.0234160

M. Osumi, M. Sumitani, Y. Nishi, S. Nobusako, B. Dilek and S. Morioka. (2021). Fear of movement-related pain disturbs cortical preparatory activity after becoming aware of motor intention. 10.1016/j.bbr.2021.113379

S. Rottmann, K. Jung, R. Vohn and J. Ellrich. (2010). Long-term depression of pain-related cerebral activation in healthy man: An fMRI study. 10.1016/j.ejpain.2009.10.006

K. Jung, S. Rottmann and J. Ellrich. (2009). Long-term depression of spinal nociception and pain in man: Influence of varying stimulation parameters. 10.1016/j.ejpain.2008.04.001

E. M. Bauch, C. Andreou, V. H. Rausch and N. Bunzeck. (2017). Neural habituation to painful stimuli is modulated by dopamine: Evidence from a pharmacological fMRI study. 10.3389/fnhum.2017.00630

B. Colagiuri, V. F. Quinn and L. Colloca. (2015). Nocebo hyperalgesia, partial reinforcement, and extinction. 10.1016j.jpain.2015.06.012

F. Fidanza, M. Varanini, A. Ciaramella, G. Carli and E. L. Santarcangelo. (2017). Pain modulation as a function of hypnotizability: Diffuse noxious inhibitory control induced by cold pressor test vs explicit suggestions of analgesia. 10.1016/j.physbeh.2017.01.013

S. Sarkar, C. J. Woolf, A. R. Hobson, D. G. Thompson and Q. Aziz. (2006). Perceptual wind-up in the human oesophagus is enhanced by central sensitisation. 10.1136/gut.2005.073643

D. Andrew and J. D. Greenspan. (1999). Peripheral coding of tonic mechanical cutaneous pain: comparison of nociceptor activity in rat and human psychophysics. 10.1152/jn.1999.82.5.2641

I. Vlaev, B. Seymour, N. Chater, J. S. Winston, W. Yoshida, N. Wright, M. Symmonds and R. Dolan . (2014). Prices need no preferences: Social trends determine decisions in experimental markets for pain relief . 10.1037a0030372

J. P. Lefaucheur, S. A. Abbas, I. Lefaucheur-Ménard, D. Rouie, D. Tebbal, J. Bismuth and T. Nordine. (2021). Small nerve fiber selectivity of laser and intraepidermal electrical stimulation: A comparative study between glabrous and hairy skin. 10.1016/j.neucli.2021.06.004

F. Mancini, N. Bolognini, P. Haggard and G. Vallar. (2012). TDCS modulation of visually induced analgesia. 10.1162jocn_a_00293

E. A. Christou, J. M. Jakobi, A. Critchlow, M. Fleshner and R. M. Enoka. (2004). The 1- to 2-Hz oscillations in muscle force are exacerbated by stress, especially in older adults. 10.1152/japplphysiol.00066.2004

K. Schmidt, K. Forkmann, C. Sinke, M. Gratz, A. Bitz and U. Bingel. (2016). The differential effect of trigeminal vs. peripheral pain stimulation on visual processing and memory encoding is influenced by pain-related fear. 10.1016j.neuroimage.2016.03.026

. (). The effect of eye orientation on slowly increasing pain. 10.1016/j.ejpain.2004.04.007

Ting, C. C., Palminteri, S., Lebreton, M., & Engelmann, J. B. (2022). The elusive effects of incidental anxiety on reinforcement-learning. 10.1037/xlm0001033

C. H. Wilder-Smith, E. Hufschmid and W. Thormann. (1998). The visceral and somatic antinociceptive effects of dihydrocodeine and its metabolite, dihydromorphine. A cross-over study with extensive and quinidine-induced poor metabolizers. 10.1046/j.1365-2125.1998.00727.x

Y. Iwama, K. Takamoto, D. Hibi, H. Nishimaru, J. Matsumoto, T. Setogawa and H. Nishijo. (2022). Young female participants show blunted placebo effects associated with blunted responses to a cue predicting a safe stimulus in the right dorsolateral prefrontal cortex. 10.3389/fnins.2022.1001177

U. Moore, V. Marsh, C. Ashton and R. Seymour. (1995). Effects of peripherally and centrally acting analgesics on somato- sensory evoked potentials [see comments]. 10.1111/j.1365-2125.1995.tb05766.x

C. Brodhun, E. Borelli and T. Weiss. (2021). Influence of acute pain on valence rating of words. 10.1371journal.pone.0248744

C. Droste, M. W. Greenlee, M. Schreck and H. Roskamm. (1991). Experimental pain thresholds and plasma beta-endorphin levels during exercise. https://pubmed.ncbi.nlm.nih.gov/2020272/

Y. Rothemund, S. Ziegler, C. Hermann, S. M. Gruesser, J. Foell, C. J. Patrick and H. Flor. (2012). Fear conditioning in psychopaths: Event-related potentials and peripheral measures. 10.1016/j.biopsycho.2012.02.011

X. Niu, M. Zhang, Z. Liu, L. Bai, C. Sun, S. Wang, X. Wang, Z. Chen, H. Chen and J. Tian. (2017). Interaction of acupuncture treatment and manipulation laterality modulated by the default mode network. 10.11771744806916683684

B. van den Berg, M. Manoochehri, M. Kasting, A. C. Schouten, F. C. T. van der Helm and J. R.Buitenweg. (2021). Multisine frequency modulation of intra-epidermal electric pulse sequences: A novel tool to study nociceptive processing. 10.1016/j.jneumeth.2021.109106

B. van den Berg, M. Manoochehri, A. C. Schouten, F. C. T. van der Helm and J. R. Buitenweg. (2022). Nociceptive Intra-epidermal Electric Stimulation Evokes Steady-State Responses in the Secondary Somatosensory Cortex. 10.1007/s10548-022-00888-y

E. Tanaka, K. Inui, T. Kida, T. Miyazaki, Y. Takeshima and R. Kakigi. (2008). A transition from unimodal to multimodal activations in four sensory modalities in humans: An electrophysiological study. 10.11861471-2202-9-116

N. Otsuru, K. Inui, K. Yamashiro, T. Miyazaki, Y. Takeshima and R. Kakigi. (2010). Assessing Aδ Fiber Function With Lidocaine Using Intraepidermal Electrical Stimulation. 10.1016/j.jpain.2009.10.001

M. Lim, M. Roosink, J. S. Kim, H. W. Kim, E. B. Lee, K. M. Son, H. A. Kim and C. K. Chung. (2016). Augmented Pain Processing in Primary and Secondary Somatosensory Cortex in Fibromyalgia: A Magnetoencephalography Study Using Intra-Epidermal Electrical Stimulation. 10.1371/journal.pone.0151776

A. Vecchio and V. De Pascalis. (2021). ERP indicators of self-pain and other pain reductions due to placebo analgesia responding: The moderating role of the fight-flight-freeze system. 10.3390/brainsci11091192

T. Iwabe, I. Ozaki and A. Hashizume. (2014). The respiratory cycle modulates brain potentials, sympathetic activity, and subjective pain sensation induced by noxious stimulation. 10.1016/j.neures.2014.03.003

Y. Shiroshita, H. Kirimoto, T. Watanabe, K. Yunoki and I. Sobue. (2021). Event-related potentials evoked by skin puncture reflect activation of Aβ fibers: Comparison with intraepidermal and transcutaneous electrical stimulations. 10.7717/peerj.12250

H. Tada, T. Torisu, M. Tanaka, H. Murata, A. De Laat and P. Svensson. (2015). Experimental low-level jaw clenching inhibits temporal summation evoked by electrical stimulation in healthy human volunteers. 10.1016j.archoralbio.2015.02.013

Reutter, M., & Gamer, M. (2022). Individual patterns of visual exploration predict the extent of fear generalization in humans. 10.1037/emo0001134

A. Sebastian, A. Schick, M. Sandner, R. M. Werzlau, A. Chmitorz, K. Lieb and O. Tüscher . (2021). Perceived threat modulates inhibitory performance . 10.1037/emo0000746;10.1037/emo0000746.supp(Supplemental)

E. M. Seidel, D. M. Pfabigan, A. Hahn, R. Sladky, A. Grahl, K. Paul, C. Kraus, M. Küblböck, G. S. Kranz, A. Hummer, R. Lanzenberger, C. Windischberger and C. Lamm. (2015). Uncertainty during pain anticipation: The adaptive value of preparatory processes. 10.1002/hbm.22661

D. Hewitt, A. Byrne, J. Henderson, A. Newton-Fenner, J. Tyson-Carr, N. Fallon, C. Brown and A.Stancak. (2021). Inhibition of cortical somatosensory processing during and after low frequency peripheral nerve stimulation in humans. 10.1016/j.clinph.2021.03.024

A. R. Lloyd, S. C. Gandevia and J. P. Hales. (1991). Muscle performance, voluntary activation, twitch properties and perceived effort in normal subjects and patients with the chronic fatigue syndrome. 10.1093oxfordjournals.brain.a101869

Xiang, Y. Jiang, Y. Ni, M. Fan, F. Shen, X. Wang, J. Han and C. Cui. (2012). Catechol-O-methyltransferase polymorphisms do not play a significant role in pain perception in male Chinese Han population. 10.1152physiolgenomics.00162.2011

S. Li, J. C. Berliner, D. H. Melton and S. Li. (2013). Modification of electrical pain threshold by voluntary breathing-controlled electrical stimulation (BreEStim) in healthy subjects. 10.1371/journal.pone.0070282

M. K. Greenwald, M. M. Bradley, B. N. Cuthbert and P. J. Lang . (1998). Startle potentiation: Shock sensitization, aversive learning, and affective picture modulation . 10.1037/0735-7044.112.5.1069

Y. Laufer. (2013). A brief interphase interval interposed within biphasic pulses enhances the contraction force of the quadriceps femoris muscle. 10.3109/09593985.2012.757405

C. R. France and S. Suchowiecki. (1999). A comparison of diffuse noxious inhibitory controls in men and women. 10.1016/S0304-3959(98)00272-3

W. R. Holcomb, S. Golestani and S. Hill. (2000). A comparison of knee-extension torque production with biphasic versus Russian current. 10.1123/jsr.9.3.229

J. Ozcan, A. R. Ward and V. J. Robertson. (2004). A comparison of true and premodulated interferential currents. 10.1016/s0003-9993(03)00478-7

H. Kaube, Z. Katsarava, T. Käufer, H. C. Diener and J. Ellrich. (2000). A new method to increase nociception specificity of the human blink reflex. 10.1016/S1388-2457(99)00295-3

M. B. Jensen, J. B. Manresa and O. K. Andersen. (2015). A new objective method for acquisition and quantification of reflex receptive fields. 10.1097/01.j.pain.0000460332.41009.4a

A. C. Lints-Martindale, T. Hadjistavropoulos, B. Barber and S. J. Gibson. (2007). A psychophysical investigation of the facial action coding system as an index of pain variability among older adults with and without Alzheimer's disease. 10.1111/j.1526-4637.2007.00358.x

A. Delitto, M. J. Strube, A. D. Shulman and S. D. Minor. (1992). A study of discomfort with electrical stimulation. 10.1093/ptj/72.6.410

Tondo, T. R.; Gill, Kinloch. (1975). A technique for the assessment of the effects of electrical aversion on human operant behavior: An exploratory study. 10.1016/S0005-7894(75)80061-X

L. Arendt-Nielsen, H. Mansikka, C. Staahl, H. Rees, K. Tan, T. S. Smart, R. Monhemius, R. Suzuki and A. M. Drewes. (2011). A translational study of the effects of ketamine and pregabalin on temporal summation of experimental pain. 10.1097/AAP.0b013e31822b0db0

T. W. Lim, T. Loh, H. Kranz and D. Scott. (1977). Acupuncture--effect on normal subjects. https://pubmed.ncbi.nlm.nih.gov/865358/

Newstead, H. Young, D. Benton, G. Jiga-Boy, M. L. Andrade Sienz, R. M. Clement and F. Boy. (2018). Acute and repetitive fronto-cerebellar tDCS stimulation improves mood in non-depressed participants. 10.1007s00221-017-5109-y

Head, P., Waldron, M., Theis, N., & Patterson, S. D. (2020). Acute Neuromuscular Electrical Stimulation (NMES) With Blood Flow Restriction: The Effect of Restriction Pressures. 10.1123/jsr.2019-0505

Silvestrini, N.; Rainville, P. (2013). After,Äéeffects of cognitive control on pain. 10.1002/j.1532-2149.2013.00299.x

Y. Tu, Z. Zhang, A. Tan, W. Peng, Y. S. Hung, M. Moayedi, G. D. Iannetti and L. Hu. (2016). Alpha and gamma oscillation amplitudes synergistically predict the perception of forthcoming nociceptive stimuli. 10.1002hbm.23048

N. Hansen, A. K. Kahn, D. Zeller, Z. Katsarava, C. Sommer and N. Üçeyler. (2015). Amplitudes of pain-related evoked potentials are useful to detect small fiber involvement in painful mixed fiber neuropathies in addition to quantitative sensory testing - An electrophysiological study. 10.3389/fneur.2015.00244

E. Ambrosini, S. Ferrante, T. Schauer, G. Ferrigno, F. Molteni and A. Pedrocchi. (2014). An automatic identification procedure to promote the use of FES-cycling training for hemiparetic patients. 10.1260/2040-2295.5.3.275

E. Olofsen, M. Boom, E. Sarton, M. van Velzen, P. Baily, K. J. Smith, A. Oksche, A. Dahan and M.Niesters. (2019). Analgesic and Respiratory Depressant Effects of R-dihydroetorphine: A Pharmacokinetic-Pharmacodynamic Analysis in Healthy Male Volunteers. 10.1097/aln.0000000000002991

S. Petersen-Felix, L. Arendt-Nielsen, P. Bak, D. Roth, M. Fischer, P. Bjerring and A. M. Zbinden. (1995). Analgesic effect in humans of subanaesthetic isoflurane concentrations evaluated by experimentally induced pain. 10.1093bja/75.1.55

E. B. Liem, C. M. Lin, M. I. Suleman, A. G. Doufas, R. G. Gregg, J. M. Veauthier, G. Loyd and D. I.Sessler. (2004). Anesthetic requirement is increased in redheads. 10.1097/00000542-200408000-00006

Bruehl, Stephen; al'Absi, Mustafa; France, Christopher R.; France, Janis; Harju, Angie; Burns, John W.; Chung, Ok Y. (2008). Anger management style and endogenous opioid function: Is gender a moderator?. 10.1007/s10865-007-9099-2

Rhudy, Jamie L.; Bartley, Emily J.; Williams, Amy E.; McCabe, Klanci M.; Chandler, Mary C.; Russell, Jennifer L.; Kerr, Kara L. (2010). Are there sex differences in affective modulation of spinal nociception and pain?. 10.1016/j.jpain.2010.04.003

Rukwied, R.; Weinkauf, B.; Main, M.; Obreja, O.; Schmelz, M. (2014). Axonal hyperexcitability after combined NGF sensitization and UV-B inflammation in humans. 10.1002/j.1532-2149.2013.00423.x

P. G. Qiao, X. Cheng, Z. W. Zuo, C. Han, Z. H. Yang and G. J. Li. (2019). Blood Oxygen Level-Dependent Response Changes in the Ipsilateral Primary Somatosensory Cortex and Thalamus of Patients With Moyamoya Disease During Median Nerve Electrical Stimulation. 10.1097/RCT.0000000000000891

J. Wiemer, S. M. Schulz, P. Reicherts, E. Glotzbach-Schoon, M. Andreatta and P. Pauli. (2014). Brain activity associated with illusory correlations in animal phobia. 10.1093/scan/nsu142

J. C. Choi, J. Kim, E. Kang, J. M. Lee, J. Cha, Y. J. Kim, H. G. Lee, J. H. Choi and D. J. Yi. (2016). Brain mechanisms of pain relief by transcutaneous electrical nerve stimulation: A functional magnetic resonance imaging study. 10.1002ejp.696

H. E. Torebjörk, L. E. Lundberg and R. H. LaMotte. (1992). Central changes in processing of mechanoreceptive input in capsaicin-induced secondary hyperalgesia in humans. 10.1113/jphysiol.1992.sp019069

Lindelof, Kim; Ellrich, Jens; Jensen, Rigmor; Bendtsen, Lars. (2009). Central pain processing in chronic tension-type headache. 10.1016/j.clinph.2009.04.013

Bocci, T., Santarcangelo, E., Vannini, B., Torzini, A., Carli, G., Ferrucci, R., Priori, A., Valeriani, M., & Sartucci, F. (2015). Cerebellar direct current stimulation modulates pain perception in humans. 10.3233/RNN-140453

K. Yamashiro, D. Sato, H. Onishi, K. Sugawara, N. Otsuru, H. Kirimoto, S. Nakazawa, Y. Yamazaki, H. Shirozu and A. Maruyama. (2019). Change-Driven M100 Component in the Bilateral Secondary Somatosensory Cortex: A Magnetoencephalographic Study. 10.1007/s10548-018-0687-y

M. J. Cohen, P. A. Ament, S. L. Schandler and M. Vulpe. (1996). Changes in the P300 component of the tactile event-related potential following spinal cord injury. 10.1038/sc.1996.19

M. L. Peters, A. J. M. Schmidt, M. A. Van den Hout, R. Koopmans and M. E. Sluijter. (1992). Chronic back pain, acute postoperative pain and the activation of diffuse noxious inhibitory controls (DNIC). 10.10160304-3959(92)90159-9

Harvie, Daniel S.; Poolman, Eva Y.; Madden, Victoria J.; Olthof, Nick A.; Coppieters, Michel W. (2022). Classically conditioned modulation of pain depends on stimulus intensity. 10.1007/s00221-021-06285-4

S. Liu, J. E. Pollock, M. F. Mulroy, H. W. Allen, J. M. Neal and R. L. Carpenter. (1995). Comparison of 5% with dextrose, 1.5% with dextrose, and 1.5% dextrose-free lidocaine solutions for spinal anesthesia in human volunteers. 10.1097/00000539-199510000-00007

W. Yuan, L. Dan, R. Netra, M. Shaohui, J. Chenwang and Z. Ming. (2013). A pharmaco-fMRI study on pain networks induced by electrical stimulation after sumatriptan injection. 10.1007/s00221-013-3405-8

N. Theysohn, K. E. Choi, E. R. Gizewski, M. Wen, T. Rampp, T. Gasser, G. J. Dobos, M. Forsting and F. Musial. (2014). Acupuncture-related modulation of pain-associated brain networks during electrical pain stimulation: A functional magnetic resonance imaging study. 10.1089/acm.2014.0105

P. Piletta, H. C. Porchet and P. Dayer. (1991). Central analgesic effect of acetaminophen but not of aspirin. 10.1038clpt.1991.40

G. Stacher, H. Steinringer, G. Schmierer, S. Winklehner and C. Schneider. (1982). Ceruletide increases threshold and tolerance to experimentally induced pain in healthy man. 10.1016/0196-9781(82)90064-X

Ward, L., Wright, E., & McMahon, S. B. . (1996). A comparison of the effects of noxious and innocuous counterstimuli on experimentally induced itch and pain. 10.1016/0304-3959(95)00080-1

L. Arendt-Nielsen, S. Petersen-Felix, M. Fischer, P. Bak, P. Bjerring and A. M. Zbinden. (1995). The effect of N-methyl-D-aspartate antagonist (ketamine) on single and repeated nociceptive stimuli: a placebo-controlled experimental human study. 10.1097/00000539-199507000-00013

Brunner, M.; L√∂ffler, M.; Kamping, S.; Bustan, S.; Gonz√°lez-Rold√°n, A. M.; Anton, F.; Flor, H. (2017). Assessing suffering in experimental pain models: Psychological and psychophysiological correlates. 10.1027/2151-2604/a000279

B. Moret, R. Camilleri Gorrieri, M. Grassi and G. Campana . (2021). Cognitive exergame training and transcranial random noise stimulation effects on executive control in healthy young adults . 10.1037/neu0000745

A. Zarkou, S. Stackhouse, S. A. Binder-Macleod and S. C. K. Lee. (2017). Comparison of techniques to determine human skeletal muscle voluntary activation. 10.1016/j.jelekin.2017.05.011

B. Rehberg, J. H. Baars, J. Kotsch, P. Koppe and F. von Dincklage. (2012). Comparison of trigeminal and spinal modulation of pain and nociception. 10.3109/00207454.2011.649868

F. Facchinetti, G. Sandrini, F. Petraglia, E. Alfonsi, G. Nappi and A. R. Genazzani. (1984). Concomitant increase in nociceptive flexion reflex threshold and plasma opioids following transcutaneous nerve stimulation. 10.10160304-3959(84)90006-x

R. Ruscheweyh, F. Weinges, M. Schiffer, M. Bäumler, M. Feller, S. Krafft, A. Straube, J. Sommer and M.Marziniak. (2015). Control over spinal nociception as quantified by the nociceptive flexor reflex (RIII reflex) can be achieved under feedback of the RIII reflex. 10.1002/ejp.570

B. A. Valdés, K. Lajoie, D. S. Marigold and C. Menon. (2021). Cortical effects of noisy galvanic vestibular stimulation using functional near-infrared spectroscopy. 10.3390/s21041476

Danziger, N., Rozenberg, S., Bourgeois, P., Charpentier, G., & Willer, J. C. (1998). Depressive effects of segmental and heterotopic application of transcutaneous electrical nerve stimulation and piezoelectric current on lower limb nociceptive flexion reflex in human subjects. 10.1016/s0003-9993(98)90299-4

C. Jurth, T. M. Dörig, G. Lichtner, A. Golebiewski, A. Jakuscheit and F. von Dincklage. (2018). Development, validation and utility of a simulation model of the nociceptive flexion reflex threshold. 10.1016j.clinph.2017.11.030

J. C. Willer and M. Ernst. (1986). Diazepam reduces stress-induced analgesia in humans. 10.10160006-8993(86)90472-5

M. L. Peters and A. J. M. Schmidt. (1992). Differences in pain perception and sensory discrimination between chronic low back pain patients and healthy controls. 10.1016/0022-3999(92)90113-G

B. Gierse-Plogmeier, R. Colak-Ekici, A. Wolowski, I. Gralow, M. Marziniak and S. Evers. (2009). Differences in trigeminal and peripheral electrical pain perception in women with and without migraine. 10.1007s10194-009-0118-2

S. S. Liu and P. D. Ware. (1997). Differential sensory block after spinal bupivacaine in volunteers. 10.109700000539-199701000-00022

Tabernig, C. B., Carrere, L. C., Manresa, J. B., & Spaich, E. G. (2021). Does feedback based on FES-evoked nociceptive withdrawal reflex condition event-related desynchronization? An exploratory study with brain-computer interfaces. 10.1088/2057-1976/ac2077

M. Elam, B. Olausson, J. O. Skarphedinsson and B. G. Wallin. (1999). Does sympathetic nerve discharge affect the firing of polymodal C-fibre afferents in humans?. 10.1093/brain/122.12.2237

E. Meine, J. Meier, B. Meyer and M. Wessa. (2021). Don't stress, it's under control: Neural correlates of stressor controllability in humans. 10.1016/j.neuroimage.2021.118701

A. Monaco, R. Cattaneo, L. Mesin, E. Ortu, M. Giannoni and D. Pietropaoli. (2015). Dysregulation of the descending pain system in temporomandibular disorders revealed by low-frequency sensory transcutaneous electrical nerve stimulation: A pupillometric study. 10.1371/journal.pone.0122826

Larbig W, Elbert T, Lutzenberger W, Rockstroh B, Schnerr G, Birbaumer N. (1982). EEG and slow brain potentials during anticipation and control of painful stimulation. 10.1016/0013-4694(82)90088-8

B. Guzmán-González, P. Llanos, J. Calatayud, N. A. Maffiuletti and C. Cruz-Montecinos. (2019). Effect of neuromuscular electrical stimulation frequency on postprandial glycemia, current-related discomfort, and muscle soreness. A crossover study. 10.1139/apnm-2018-0801

S. J. Aboodarda, J. Mira, M. Floreani, R. Jaswal, S. J. Moon, K. Amery, T. Rupp and G. Y. Millet. (2018). Effects of endurance cycling training on neuromuscular fatigue in healthy active men. Part II: Corticospinal excitability and voluntary activation. 10.1007/s00421-018-3951-7

G. Sandrini, C. Tassorelli, A. P. Cecchini, E. Alfonsi and G. Nappi. (2002). Effects of nimesulide on nitric oxide-induced hyperalgesia in humans--a neurophysiological study. 10.1016/s0014-2999(02)02188-x

J. W. Bellew, M. Allen, A. Biefnes, S. Grantham, J. Miglin and D. Swartzell. (2018). Efficiency of neuromuscular electrical stimulation: A comparison of elicited force and subject tolerance using three electrical waveforms. 10.1080/09593985.2017.1422820

I. M. Oliveira, A. R. P. Machado, A. A. Pereira and A. O. Andrade . (2014). Estimated features from surface EMG of the lower limb correlate with the subjective sensation of pain . 10.3922/j.psns.2014.044

E. L. Terry, K. A. Thompson and J. L. Rhudy. (2015). Experimental reduction of pain catastrophizing modulates pain report but not spinal nociception as verified by mediation analyses. 10.1097/j.pain.0000000000000192

L. Bendtsen, R. Jensen, J. Brennum, L. Arendt-Nielsen and J. Olesen. (1996). Exteroceptive suppression of temporal muscle activity is normal in chronic tension-type headache and not related to actual headache state. 10.1046j.1468-2982.1996.1604251.x

J. C. Smith, D. P. Stephens, P. K. Winchester and J. W. Williamson. (1997). Facial cooling-induced bradycardia: Attenuating effect of central command at exercise onset. 10.1097/00005768-199703000-00006

S. Liu, A. A. Chiu, R. L. Carpenter, M. F. Mulroy, H. W. Allen, J. M. Neal and J. E. Pollock. (1995). Fentanyl prolongs lidocaine spinal anesthesia without prolonging recovery. 10.1097/00000539-199504000-00014

Armando Perrotta MD, PhD, Maria Grazia Anastasio MD, PhD, Roberto De Icco MD, Gianluca Coppola MD, PhD, Anna Ambrosini MD, PhD, Mariano Serrao MD, PhD, Giorgio Sandrini MD, Prof, Francesco Pierelli MD, Prof . (2017). Frequency-Dependent Habituation Deficit of the Nociceptive Blink Reflex in Aura With Migraine Headache. Can Migraine Aura Modulate Trigeminal Excitability?. 10.1111/head.13111

C. Vogel, R. Rukwied, L. Stockinger, M. Schley, M. Schmelz, W. Schleinzer and C. Konrad. (2017). Functional Characterization of At-Level Hypersensitivity in Patients With Spinal Cord Injury. 10.1016/j.jpain.2016.10.003

C. F. Samer, Y. Daali, M. Wagner, G. Hopfgartner, C. B. Eap, M. C. Rebsamen, M. F. Rossier, D. Hochstrasser, P. Dayer and J. A. Desmeules. (2010). Genetic polymorphisms and drug interactions modulating CYP2D6 and CYP3A activities have a major effect on oxycodone analgesic efficacy and safety. 10.1111/j.1476-5381.2010.00709.x

Tatsuya Kondo 1, Kazunari Sasaki 1, Hironori Adachi 1, Yoshiharu Nakayama 1, Masahiro Hatemura 1, Rina Matsuyama 1, Kaku Tsuruzoe 1, Noboru Furukawa 1, Hiroyuki Motoshima 1, Saori Morino Koga 1, Yasuyuki Yamashita 1, Nobuhiro Miyamura 1, Hirofumi Kai 1, Eiichi Araki 2. (2009). Heat shock treatment with mild electrical stimulation safely reduced inflammatory markers in healthy male subjects. 10.1016/j.orcp.2009.09.007

Carvalho VO, Roque JM, Bocchi EA, Ciolac EG, Guimarães GV. . (2011). Hemodynamic response in one session of strength exercise with and without electrostimulation in heart failure patients: A randomized controlled trial. -

E. R. Ferrè, B. L. Day, G. Bottini and P. Haggard. (2013). How the vestibular system interacts with somatosensory perception: A sham-controlled study with galvanic vestibular stimulation. 10.1016/j.neulet.2013.06.046

C. Lopez, B. Lenggenhager and O. Blanke. (2010). How vestibular stimulation interacts with illusory hand ownership. 10.1016/j.concog.2009.12.003

P. Compton, C. P. Canamar, M. Hillhouse and W. Ling. (2012). Hyperalgesia in heroin dependent patients and the effects of opioid substitution therapy. 10.1016/j.jpain.2012.01.001

Ayesh, E. E., Jensen, T. S., & Svensson, P. (2007). Hypersensitivity to mechanical and intra-articular electrical stimuli in persons with painful temporomandibular joints. 10.1177/154405910708601209

R. Duranti, T. Pantaleo and F. Bellini. (1988). Increase in muscular pain threshold following low frequency-high intensity peripheral conditioning stimulation in humans. 10.1016/0006-8993(88)90009-1

L. Edwards, C. Ring, D. McIntyre, D. Carroll, R. Clarke, O. Webb and U. Martin . (2006). Increases in arousal are associated with reductions in the human nociceptive flexion reflex threshold and pain ratings: Evidence for dissociation between nociception and pain . 10.1027/0269-8803.20.4.259

A. Ladouceur, N. Rustamov, J. D. Dubois, J. Tessier, A. Lehmann, M. Descarreaux, P. Rainville and M.Piché. (2018). Inhibition of Pain and Pain-Related Brain Activity by Heterotopic Noxious Counter-Stimulation and Selective Attention in Chronic Non-Specific Low Back Pain. 10.1016/j.neuroscience.2017.09.054

Nabi Rustamov, Jessica Tessier, Benjamin Provencher, Alexandre Lehmann, Mathieu Piché. (2016). Inhibitory effects of heterotopic noxious counter-stimulation on perception and brain activity related to Aβ-fibre activation. 10.1111/ejn.13258

C. J. Merz, K. Tabbert, J. Schweckendiek, T. Klucken, D. Vaitl, R. Stark and O. T. Wolf. (2010). Investigating the impact of sex and cortisol on implicit fear conditioning with fMRI. 10.1016/j.psyneuen.2009.07.009

G. Alder, N. Signal, A. C. Vandal, S. Olsen, M. Jochumsen, I. K. Niazi and D. Taylor. (2021). Investigating the intervention parameters of endogenous paired associative stimulation (ePAS). 10.3390/brainsci11020224

E. Kuatsjah, M. Khoshnam and C. Menon. (2019). Investigation on the effect of noisy galvanic vestibular stimulation on fine motor skills during a visuomotor task in healthy participants. 10.1371/journal.pone.0216214

S. A. Bouwense, S. S. Olesen, A. M. Drewes, J. B. Frøkjær, H. van Goor and O. H. Wilder-Smith. (2013). Is altered central pain processing related to disease stage in chronic pancreatitis patients with pain? An exploratory study. 10.1371/journal.pone.0055460

J. A. Biurrun Manresa, R. Fritsche, P. H. Vuilleumier, C. Oehler, C. D. Mørch, L. Arendt-Nielsen, O. K. Andersen and M. Curatolo. (2014). Is the conditioned pain modulation paradigm reliable? A test-retest assessment using the nociceptive withdrawal reflex. 10.1371/journal.pone.0100241

M. Dusch, J. Van Der Ham, B. Weinkauf, J. Benrath, R. Rukwied, M. Ringkamp, M. Schmelz, R. D. Treede and U. Baumgärtner. (2016). Laser-evoked potentials mediated by mechano-insensitive nociceptors in human skin. 10.1002/ejp.810

Y. Jiang, H. Wang, Z. Liu, Y. Dong, Y. Dong, X. Xiang, L. Bai, J. Tian, L. Wu, J. Han and C. Cui. (2013). Manipulation of and Sustained Effects on the Human Brain Induced by Different Modalities of Acupuncture: An fMRI Study. 10.1371journal.pone.0066815

O. Telli and U. Cavlak. (2006). Measuring the pain threshold and tolerance using electrical stimulation in patients with Type II diabetes mellitus. 10.1016/j.jdiacomp.2005.07.004

B. Weinkauf, M. Main, M. Schmelz and R. Rukwied. (2013). Modality-specific nociceptor sensitization following UV-B irradiation of human skin. 10.1016/j.jpain.2013.02.007

J. L. Rhudy, N. Hellman, C. A. Sturycz, T. A. Toledo and S. Palit. (2020). Modified biofeedback (conditioned biofeedback) promotes antinociception by increasing the nociceptive flexion reflex threshold and reducing temporal summation of pain: A controlled trial. 10.1016/j.jpain.2019.10.006

W. T. Zhang, Z. Jin, J. Huang, L. Zhang, Y. W. Zeng, F. Luo, A. C. N. Chen and J. S. Han. (2003). Modulation of cold pain in human brain by electric acupoint stimulation: Evidence from fMRI. 10.1097/00001756-200308260-00010

V. Mylius, J. Reis, M. Kunz, T. F. Beyer, W. H. Oertel, F. Rosenow and K. Schepelmann. (2006). Modulation of electrically induced pain by paired pulse transcranial magnetic stimulation of the medial frontal cortex. 10.1016j.clinph.2006.04.013

Hansen, N., Obermann, M., Poitz, F., Holle, D., Diener, H. C., Antal, A., Paulus, W., & Katsarava, Z. (2011). Modulation of human trigeminal and extracranial nociceptive processing by transcranial direct current stimulation of the motor cortex. 10.1177/0333102410390394

M. Luginbühl, A. Gerber, T. W. Schnider, S. Petersen-Felix, L. Arendt-Nielsen and M. Curatolo. (2003). Modulation of remifentanil-induced analgesia, hyperalgesia, and tolerance by small-dose ketamine in humans. 10.121301.Ane.0000048086.58161.18

C. Donnelly, J. Stegmüller, A. J. Blazevich, F. Crettaz von Roten, B. Kayser, D. Neyroud and N.Place. (2021). Modulation of torque evoked by wide-pulse, high-frequency neuromuscular electrical stimulation and the potential implications for rehabilitation and training. 10.1038/s41598-021-85645-0

P. Okkerse, R. Alvarez-Jimenez, J. L. Hay, A. Tehim, R. Kumar, M. L. de Kam and G. J. Groeneveld. (2017). No evidence of potentiation of buprenorphine by milnacipran in healthy subjects using a nociceptive test battery. 10.1002/ejp.943

L. Edwards, C. Ring, R. C. France, M. al'Absi, D. McIntyre, D. Carroll and U. Martin. (2007). Nociceptive flexion reflex thresholds and pain during rest and computer game play in patients with hypertension and individuals at risk for hypertension. 10.1016/j.biopsycho.2007.06.005

J. Bartley, S. Palit, B. L. Kuhn, K. L. Kerr, E. L. Terry, J. L. DelVentura and J. L. Rhudy. (2015). Nociceptive processing in women with premenstrual dysphoric disorder (PMDD): The role of menstrual phase and sex hormones. 10.1097AJP.0000000000000112
J. A. B. Manresa, F. G. Arguissain, D. E. M. Redondo, C. D. Mørch and O. K. Andersen. (2015). On the agreement between manual and automated methods for single-trial detection and estimation of features from event-related potentials. 10.1371/journal.pone.0134127
S. Petersen-Felix, L. Arendt-Nielsen, P. Bak, P. Bjerring, H. Breivik, P. Svensson and A. M.Zbinden. (1994). Ondansetron does not inhibit the analgesic effect of alfentanil. 10.1093/bja/73.3.326
S. C. Manyam, D. K. Gupta, K. B. Johnson, J. L. White, N. L. Pace, D. R. Westenskow and T. D.Egan. (2006). Opioid-volatile anesthetic synergy: a response surface model with remifentanil and sevoflurane as prototypes. 10.1097/00000542-200608000-00009
P. W. Cheng, C. S. Yang, T. W. Huang and Y. H. Young. (2008). Optimal stimulation mode for galvanic-evoked myogenic potentials. 10.1097/AUD.0b013e31818713c3
S. Liu, A. A. Chiu, J. M. Neal, R. L. Carpenter, B. G. Bainton and J. C. Gerancher. (1995). Oral clonidine prolongs lidocaine spinal anesthesia in human volunteers. 10.1097/00000542-199506000-00005
D. E. Washington, D. I. Sessler, J. McGuire, J. Hynson, M. Schroeder and A. Moayeri. (1992). Painful stimulation minimally increases the thermoregulatory threshold for vasoconstriction during enflurane anesthesia in humans. 10.1097/00000542-199208000-00011
Tsao, Y. C., Lai, K. L., Chen, J. T., Liao, K. K., & Wang, S. J. . (2019). Paired nociceptive blink stimuli can facilitate trigeminofacial circuit at a long inter-stimulus interval. 10.1097/JCMA.0000000000000003
L. Kuhlmann, S. S. Olesen, D. Grønlund, A. E. Olesen, A. E. Phillips, M. Faghih and A. M. Drewes. (2019). Patient and disease characteristics associate with sensory testing results in chronic pancreatitis. 10.1097AJP.0000000000000740
M. Escher, Y. Daali, J. Chabert, G. Hopfgartner, P. Dayer and J. Desmeules. (2007). Pharmacokinetic and pharmacodynamic properties of buprenorphine after a single intravenous administration in healthy volunteers: a randomized, double-blind, placebo-controlled, crossover study. 10.1016/j.clinthera.2007.08.007
E. Rostrup, M. H. E. Christensen, R. Seifert and J. E. Nordrehaug. (2012). Physiological effects of combined thermal and electrical muscle stimulation (cTEMS) in healthy individuals: A pilot study. 10.3109/00365513.2012.657666
J. Valls-Solé, M. Veciana, J. Serra, G. Cruccu and A. Romaniello. (2000). Prepulse inhibition of the blink reflex by laser stimuli in normal humans. 10.1016/S0304-3940(00)01085-5
M. E. Kızıltan, P. Bekdik Şirinocak, T. Akıncı, T. Cerrahoğlu Şirin, B. N. Arkalı, F. Candan and A.Gündüz. (2019). Prepulse modulation and recovery of trigemino-cervical reflex in normal subjects. 10.1007s10072-018-3624-7
N. R. Swerdlow, J. M. Shoemaker, N. Stephany, L. Wasserman, H. J. Ro and M. A. Geyer . (2002). Prestimulus effects on startle magnitude: Sensory or motor? . 10.1037/0735-7044.116.4.672
S. Hughes, S. Grimsey and P. H. Strutton. (2019). Primary motor cortex transcranial direct current stimulation modulates temporal summation of the nociceptive withdrawal reflex in healthy subjects. 10.1093/pm/pny200
S. M. Schabrun, M. C. Ridding, M. P. Galea, P. W. Hodges and L. S. Chipchase. (2012). Primary Sensory and Motor Cortex Excitability Are Co-Modulated in Response to Peripheral Electrical Nerve Stimulation. 10.1371journal.pone.0051298
Sheather-Reid, R. B., & Cohen, M. L. . (1998). Psychophysical evidence for a neuropathic component of chronic neck pain. 10.1016/S0304-3959(98)00013-X
M. Yüksel, Ş. Ayaş, M. T. Cabioğlu, D. Yilmaz and C. Cabioğlu. (2019). Quantitative Data for Transcutaneous Electrical Nerve Stimulation and Acupuncture Effectiveness in Treatment of Fibromyalgia Syndrome. 10.1155/20199684649
J. Brennum, L. Arendt-Nielsen, A. Horn, N. H. Secher and T. S. Jensen. (1993). Quantitative sensory examination during epidural anaesthesia and analgesia in man: effects of morphine. 10.1016/0304-3959(93)90117-8
J. Brennum, K. L. Petersen, A. Horn, L. Arendt-Nielsen, N. H. Secher and T. S. Jensen. (1994). Quantitative sensory examination of epidural anaesthesia and analgesia in man: Combination of morphine and bupivacaine. 10.10160304-3959(94)90171-6
J. Brennum, P. T. Nielsen, A. Horn, L. Arendt-Nielsen and N. H. Secher. (1994). Quantitative sensory examination of epidural anaesthesia and analgesia in man: Dose-response effect of bupivacaine. 10.10160304-3959(94)90170-8
S. A. Janssen and A. Arntz . (2001). Real-life stress and opioid-mediated analgesia in novice parachute jumpers . 10.1027//0269-8803.15.2.106
A. Gündüz, U. Uygunoğlu, D. Uluduz, S. Saip, A. Siva, B. Göksan, M.E. Kızıltan. (2018). Reduced inhibition in brainstem circuits in classical trigeminal neuralgia. 10.1002/ejp.1293
M. Pitman and J. G. Semmler. (2012). Reduced short-interval intracortical inhibition after eccentric muscle damage in human elbow flexor muscles. 10.1152/japplphysiol.00361.2012
H. Schulte, M. Segerdahl, T. Graven-Nielsen and S. Grass. (2006). Reduction of human experimental muscle pain by alfentanil and morphine. 10.1016/j.ejpain.2005.11.006
R. Duranti, R. Galletti and T. Pantaleo. (1983). Relationships between characteristics of electrical stimulation, muscle pain and blink responses in man. 10.1016/0013-4694(83)90273-0
P. S. Siebenga, G. van Amerongen, P. Okkerse, W. S. Denney, P. Dua, R. P. Butt, J. L. Hay and G. J.Groeneveld. (2019). Reproducibility of a battery of human evoked pain models to detect pharmacological effects of analgesic drugs. 10.1002/ejp.1379
G. S. Hughes, Jr., P. R. Lichstein, D. Whitlock and C. Harker. (1984). Response of plasma beta-endorphins to transcutaneous electrical nerve stimulation in healthy subjects. 10.1093/ptj/64.7.1062
Haberbosch, L., Datta, A., Thomas, C., Jooß, A., Köhn, A., Rönnefarth, M., Scholz, M., Brandt, S. A., & Schmidt, S. (2019). Safety Aspects, Tolerability and Modeling of Retinofugal Alternating Current Stimulation. 10.3389/fnins.2019.00783
Annalisa Monaco a, Ruggero Cattaneo a, Eleonora Ortu a, Marian Vladimir Constantinescu b, Davide Pietropaoli. (2017). Sensory trigeminal ULF-TENS stimulation reduces HRV response to experimentally induced arithmetic stress: A randomized clinical trial. 10.1016/j.physbeh.2017.02.014
S. Palit, R. J. Sheaff, C. R. France, S. T. McGlone, W. T. Potter, A. R. Harkness, J. L. McNulty, E. J. Bartley, R. Hoffmann, J. K. Monda and J. L. Rhudy. (2011). Serotonin transporter gene (5-HTTLPR) polymorphisms are associated with emotional modulation of pain but not emotional modulation of spinal nociception. 10.1016j.biopsycho.2011.01.008
P. Goffaux, K. Michaud, J. Gaudreau, P. Chalaye, P. Rainville and S. Marchand. (2011). Sex differences in perceived pain are affected by an anxious brain. 10.1016/j.pain.2011.05.002
P. Svensson, C. H. Hashikawa and K. L. Casey. (1999). Site- and modality-specific modulation of experimental muscle pain in humans. 10.1016/S0006-8993(99)02073-9
F. V. A. Medeiros, A. Vieira, R. L. Carregaro, M. Bottaro, N. A. Maffiuletti and J. L. Q. Durigan. (2015). Skinfold thickness affects the isometric knee extension torque evoked by neuromuscular electrical stimulation. 10.1590bjpt-rbf.2014.0114
M. E. Kouri and D. J. Kopacz. (2004). Spinal 2-chloroprocaine: a comparison with lidocaine in volunteers. 10.121301.Ane.0000093228.61443.Ee
D. T. Warren and D. J. Kopacz. (2004). Spinal 2-chloroprocaine: the effect of added dextrose. 10.121301.Ane.0000093362.95618.89
E. L. Terry, C. R. France, E. J. Bartley, J. L. Delventura, K. L. Kerr, A. L. Vincent and J. L. Rhudy. (2011). Standardizing procedures to study sensitization of human spinal nociceptive processes: comparing parameters for temporal summation of the nociceptive flexion reflex (TS-NFR). 10.1016/j.ijpsycho.2011.06.021
M. Fechir a, T. Schlereth a, S. Kritzmann a, S. Balon a, N. Pfeifer a, C. Geber a, M. Breimhorst a, T. Eberle a, M. Gamer b, F. Birklein a. (2009). Stress and thermoregulation: Different sympathetic responses and different effects on experimental pain. 10.1016/j.ejpain.2008.11.002
F. Boureau, M. Luu and J. F. Doubrère. (1991). Study of experimental pain measures and nociceptive reflex in chronic pain patients and normal subjects. 10.1016/0304-3959(91)90126-I
M. K. Boettger, W. Greiner, T. Rachow, C. Brühl and K. J. Bär. (2010). Sympathetic skin response following painful electrical stimulation is increased in major depression. 10.1016/j.pain.2010.01.022
A. Perrotta, P. Chiacchiaretta, M. G. Anastasio, L. Pavone, G. Grillea, M. Bartolo, E. Siravo, C. Colonnese, R. De Icco, M. Serrao, G. Sandrini, F. Pierelli and A. Ferretti. (2017). Temporal summation of the nociceptive withdrawal reflex involves deactivation of posterior cingulate cortex. 10.1002/ejp.923
T. Schunck, G. Erb, A. Mathis, N. Jacob, C. Gilles, I. J. Namer, D. Meier and R. Luthringer. (2008). Test-retest reliability of a functional MRI anticipatory anxiety paradigm in healthy volunteers. 10.1002/jmri.21237
J. C. Willer, R. Von Frenkell, D. Bonnet and G. Le Fur. (1986). The ability of PK 8165, a quinoline derivative, to reduce responses to a stressful situation in a double-blind study in man. 10.1016/0028-3908(86)90252-2
R. Ruscheweyh, A. Kreusch, C. Albers, J. Sommer and M. Marziniak. (2011). The effect of distraction strategies on pain perception and the nociceptive flexor reflex (RIII reflex). 10.1016/j.pain.2011.08.016
J. E. Sanderson, B. Tomlinson, M. S. W. Lau, K. W. H. So, A. H. K. Cheung, J. A. J. H. Critchley and K. S. Woo. (1995). The effect of transcutaneous electrical nerve stimulation (TENS) on autonomic cardiovascular reflexes. 10.1007/BF01827467
L. Poulsen, L. Arendt-Nielsen, K. Brøsen, K. K. Nielsen and L. F. Gram. (1995). The hypoalgesic effect of imipramine in different human experimental pain models. 10.1016/0304-3959(94)00142-2
M. Wang, E. C. Lin, I. Maranets and Z. N. Kain. (2009). The impact of asynchronous electroacupuncture stimulation duration on cold thermal pain threshold. 10.1213/ane.0b013e3181ad9292
Emma L Healey 1, Adrian M Burden, Islay M McEwan, Neil E Fowler. (2008). The impact of increasing paraspinal muscle activity on stature recovery in asymptomatic people. 10.1016/j.apmr.2007.09.044
P. Kemppainen, C. Forster and H. O. Handwerker. (2001). The importance of stimulus site and intensity in differences of pain-induced vascular reflexes in human orofacial regions. 10.1016/S0304-3959(00)00462-0
Nahian S Chowdhury 1, Nigel C Rogasch 2, Alan K I Chiang 3, Samantha K Millard 4, Patrick Skippen 3, Wei-Ju Chang 3, Katarzyna Bilska 4, Emily Si 3, David A Seminowicz 5, Siobhan M Schabrun 3. (2022). The influence of sensory potentials on transcranial magnetic stimulation - Electroencephalography recordings. 10.1016/j.clinph.2022.05.015
J. Lötsch and M. S. Angst. (2003). The mu-opioid agonist remifentanil attenuates hyperalgesia evoked by blunt and punctuated stimuli with different potency: a pharmacological evaluation of the freeze lesion in humans. 10.1016s0304-3959(02)00349-4
A. Y. Neziri, O. K. Andersen, S. Petersen-Felix, B. Radanov, A. H. Dickenson, P. Scaramozzino, L. Arendt-Nielsen and M. Curatolo. (2010). The nociceptive withdrawal reflex: Normative values of thresholds and reflex receptive fields. 10.1016/j.ejpain.2009.04.010
R. Dowman and S. Schell. (1999). The pain-related negative difference potential: A direct measure of central pain pathway activity or of interactions between the innocuous somatosensory and pain pathways?. 10.1016S0987-7053(00)87266-4
N. Silvestrini, E. Vuignier, A. Matthey and V. Piguet . (2019). The perception of available resources influences the after-effect of cognitive control on cognitive performance and pain . 10.1027/1864-9335/a000386
F. A. Huber, T. A. Toledo, G. Newsom and J. L. Rhudy. (2022). The relationship between sleep quality and emotional modulation of spinal, supraspinal, and perceptual measures of pain. 10.1016/j.biopsycho.2022.108352
T. Forkmann, M. Heins, T. Bruns, W. Paulus and B. Kröner-Herwig. (2009). The second exteroceptive suppression is affected by psychophysiological factors. 10.1016/j.jpsychores.2008.12.008
Y. Ginosar, E. T. Riley and M. S. Angst. (2003). The Site of Action of Epidural Fentanyl in Humans: The Difference Between Infusion and Bolus Administration. 10.1213/01.ANE.0000081793.60059.10
C. Stein, P. Dal Lago, J. B. Ferreira, K. R. Casali and R. D. M. Plentz. (2011). Transcutaneous electrical nerve stimulation at different frequencies on heart rate variability in healthy subjects. 10.1016/j.autneu.2011.07.003
L. Oudejans, M. van Velzen, E. Olofsen, R. Beun, A. Dahan and M. Niesters. (2016). Translation of random painful stimuli into numerical responses in fibromyalgia and perioperative patients. 10.1097/j.pain.0000000000000338
Serrao, M., Rossi, P., Parisi, L., Perrotta, A., Bartolo, M., Cardinali, P., Amabile, G., & Pierelli, F. (2003). Trigemino-cervical-spinal reflexes in humans. 10.1016/s1388-2457(03)00132-9
C. R. France, J. L. Rhudy and S. McGlone. (2009). Using normalized EMG to define the nociceptive flexion reflex (NFR) threshold: Further evaluation of standardized NFR scoring criteria. 10.1016/j.pain.2009.06.022
J. Quaghebeur and J. J. Wyndaele. (2015). Variability of pudendal and median nerve sensory perception thresholds in healthy persons. 10.1002/nau.22565
M. Kunz, V. Mylius, S. Scharmann, K. Schepelman and S. Lautenbacher. (2009). Influence of dementia on multiple components of pain. 10.1016/j.ejpain.2008.05.001
D. G. Buma, J. R. Buitenweg and P. H. Veltink. (2007). Intermittent stimulation delays adaptation to electrocutaneous sensory feedback. 10.1109/TNSRE.2007.903942
A. K. Frew and P. D. Drummond. (2007). Negative affect, pain and sex: The role of endogenous opioids. 10.1016j.pain.2007.04.010
Perrotta, A., Coppola, G., Anastasio, M. G., De Icco, R., Ambrosini, A., Serrao, M., Parisi, V., Evangelista, M., Sandrini, G., & Pierelli, F. (2018). Trait- and Frequency-Dependent Dysfunctional Habituation to Trigeminal Nociceptive Stimulation in Trigeminal Autonomic Cephalalgias. 10.1016/j.jpain.2018.03.015
S. Petersen-Felix, M. Luginbühl, T. W. Schnider, M. Curatolo, L. Arendt-Nielsen and A. M.Zbinden. (1998). Comparison of the analgesic potency of xenon and nitrous oxide in humans evaluated by experimental pain. 10.1093/bja/81.5.742
G. Stacher, P. Bauer, C. Schneider, S. Winklehner and G. Schmierer. (1982). Effects of a combination of oral naproxen sodium and codeine on experimentally induced pain. 10.1007/bf00542043
R. Zachariae, O. K. Andersen, P. Bjerring, M. M. Jørgensen and L. Arendt-Nielsen. (1998). Effects of an opioid antagonist on pain intensity and withdrawal reflexes during induction of hypnotic analgesia in high - and low-hypnotizable volunteers. 10.1016/S1090-3801(98)90043-X
L. Arendt-Nielsen, J. B. Frøkjaer, C. Staahl, T. Graven-Nielsen, J. P. Huggins, T. S. Smart and A. M. Drewes. (2007). Effects of gabapentin on experimental somatic pain and temporal summation. 10.1016j.rapm.2007.05.002
G. Stacher, H. Steinringer, S. Winklehner, G. Mittelbach and C. Schneider. (1983). Effects of graded oral doses of meptazinol and pentazocine in comparison with placebo on experimentally induced pain in healthy humans. 10.1111/j.1365-2125.1983.tb04979.x
A. E. Olesen, C. Staahl, Z. Ali, A. M. Drewes and L. Arendt-Nielsen. (2007). Effects of paracetamol combined with dextromethorphan in human experimental muscle and skin pain. 10.1111/j.1742-7843.2007.00095.x
T. K. Kim, P. J. Niklewski, J. F. Martin, S. Obara and T. D. Egan. (2015). Enhancing a sedation score to include truly noxious stimulation: the Extended Observer's Assessment of Alertness and Sedation (EOAA/S). 10.1093/bja/aev306
C. M. Campbell, C. R. France, M. E. Robinson, H. L. Logan, G. R. Geffken and R. B. Fillingim. (2008). Ethnic differences in diffuse noxious inhibitory controls. 10.1016/j.jpain.2008.03.010
E. M. Jegindø, L. Vase, J. C. Skewes, A. J. Terkelsen, J. Hansen, A. W. Geertz, A. Roepstorff and T. S. Jensen. (2013). Expectations contribute to reduced pain levels during prayer in highly religious participants. 10.1007/s10865-012-9438-9
G. Stacher, H. Steinringer, S. Schneider, G. Mittelbach, S. Winklehner and G. Gaupmann. (1986). Experimental pain induced by electrical and thermal stimulation of the skin in healthy man: sensitivity to 75 and 150 mg diclofenac sodium in comparison with 60 mg codeine and placebo. 10.1111/j.1365-2125.1986.tb02820.x
S. Palit, K. L. Kerr, B. L. Kuhn, E. L. Terry, J. L. DelVentura, E. J. Bartley, J. O. Shadlow and J. L.Rhudy. (2013). Exploring pain processing differences in Native Americans. 10.1037/a0031057
Prem Bajaj a b, Lars Arendt-Nielsen a, Ole K. Andersen a. (2004). Facilitation and inhibition of withdrawal reflexes following repetitive stimulation: electro- and psychophysiological evidence for activation of noxious inhibitory controls in humans. 10.1016/j.ejpain.2004.02.009
A. Huber, F. Lui and C. A. Porro. (2013). Hypnotic susceptibility modulates brain activity related to experimental placebo analgesia. 10.1016/j.pain.2013.03.031
G. Stacher, T. A. Abatzi, F. Schulte, C. Schneider, G. Stacher-Janotta, G. Gaupmann, G. Mittelbach and H.Steinringer. (1988). Naloxone does not alter the perception of pain induced by electrical and thermal stimulation of the skin in healthy humans. 10.1016/0304-3959(88)90122-4
M. Fechir, M. Breimhorst, S. Kritzmann, C. Geber, T. Schlereth, B. Baier and F. Birklein. (2012). Naloxone inhibits not only stress-induced analgesia but also sympathetic activation and baroreceptor-reflex sensitivity. 10.1016j.ejpain.2011.06.009
C. R. France, M. al'Absi, C. Ring, J. L. France, A. Harju and L. E. Wittmers. (2007). Nociceptive flexion reflex and pain rating responses during endogenous opiate blockade with naltrexone in healthy young adults. 10.1016j.biopsycho.2006.12.005
P. S. Micalos and S. C. Pak. (2011). Noneffect of manual needle acupuncture on experimental pain parameters in healthy young men. 10.1089/acm.2010.0040
J. L. Rhudy, S. L. Martin, E. L. Terry, C. R. France, E. J. Bartley, J. L. DelVentura and K. L. Kerr. (2011). Pain catastrophizing is related to temporal summation of pain but not temporal summation of the nociceptive flexion reflex. 10.1016/j.pain.2010.12.041
Schliessbach, C. Lütolf, K. Streitberger, P. Scaramozzino, L. Arendt-Nielsen and M. Curatolo. (2019). Reference values of conditioned pain modulation. 10.1515/sjpain-2018-0356
P. S. Micalos, E. J. Drinkwater, J. Cannon, L. Arendt-Nielsen and F. E. Marino. (2009). Reliability of the nociceptive flexor reflex (RIII) threshold and association with Pain threshold. 10.1007/s00421-008-0872-x
M. Curatolo, S. Petersen-Felix, A. Gerber and L. Arendt-Nielsen. (2000). Remifentanil inhibits muscular more than cutaneous pain in humans. 10.1093/bja/85.4.529
H. Jafari, K. Van de Broek, L. Plaghki, J. W. S. Vlaeyen, O. Van den Bergh and I. Van Diest. (2016). Respiratory hypoalgesia? Breath-holding, but not respiratory phase modulates nociceptive flexion reflex and pain intensity. 10.1016/j.ijpsycho.2016.01.005
R. D. Rouleau, L. Lagrandeur, K. Daigle, D. Lorrain, G. Léonard, K. Whittingstall and P. Goffaux. (2015). Significance of non-phase locked oscillatory brain activity in response to noxious stimuli. 10.1017/cjn.2015.294
Thomas P. Enggaard a b, Niels A. Klitgaard c, Søren H. Sindrup. (2006). Specific effect of levetiracetam in experimental human pain models. 10.1016/j.ejpain.2005.03.011
M. Naef, M. Curatolo, S. Petersen-Felix, L. Arendt-Nielsen, A. Zbinden and R. Brenneisen. (2003). The analgesic effect of oral delta-9-tetrahydrocannabinol (THC), morphine, and a THC-morphine combination in healthy subjects under experimental pain conditions. 10.1016/s0304-3959(03)00163-5
S. T. Zwisler, T. P. Enggaard, L. Noehr-Jensen, S. Mikkelsen, C. Verstuyft, L. Becquemont, S. H. Sindrup and K. Brosen. (2010). The antinociceptive effect and adverse drug reactions of oxycodone in human experimental pain in relation to genetic variations in the OPRM1 and ABCB1 genes. 10.1111/j.1472-8206.2009.00781.x
Y. Boyle, D. Fernando, H. Kurz, S. R. Miller, M. Zucchetto and J. Storey. (2014). The effect of a combination of gabapentin and donepezil in an experimental pain model in healthy volunteers: Results of a randomized controlled trial. 10.1016/j.pain.2014.09.003
S. S. Liu, J. C. Gerancher, B. G. Bainton, D. J. Kopacz and R. L. Carpenter. (1996). The effects of electrical stimulation at different frequencies on perception and pain in human volunteers: epidural versus intravenous administration of fentanyl. 10.1097/00000539-199601000-00017
K. Karos, A. Meulders, L. Goubert and J. W. S. Vlaeyen. (2018). The influence of social threat on pain, aggression, and empathy in women. 10.1016/j.jpain.2017.11.003
P. Okkerse, G. van Amerongen, M. L. de Kam, J. Stevens, R. P. Butt, R. Gurrell, A. Dahan, J. M. van Gerven, J. L. Hay and G. J. Groeneveld. (2017). The use of a battery of pain models to detect analgesic properties of compounds: a two-part four-way crossover study. 10.1111/bcp.13183
P. Arnold, P. Vuadens, T. Kuntzer, C. Gobelet and O. Deriaz. (2008). Mirtazapine decreases the pain feeling in healthy participants. 10.1097/AJP.0b013e318159f94a
T. Hummel, M. Springborn, I. Croy, J. Kaiser and J. Lötsch. (2011). High pain sensitivity is distinct from high susceptibility to non-painful sensory input at threshold level. 10.1016/j.ijpsycho.2011.01.012
F. Kalbe and L. Schwabe . (2022). On the search for a selective and retroactive strengthening of memory: Is there evidence for category-specific behavioral tagging? . 10.1037/xge0001075;10.1037/xge0001075.supp(Supplemental)
E. Sarton, E. Olofsen, R. Romberg, J. den Hartigh, B. Kest, D. Nieuwenhuijs, A. Burm, L. Teppema and A. Dahan. (2000). Sex differences in morphine analgesia: an experimental study in healthy volunteers. 10.109700000542-200011000-00018
T. Mankovsky-Arnold, T. H. Wideman, C. Larivière and M. J. Sullivan. (2013). TENS attenuates repetition-induced summation of activity-related pain following experimentally induced muscle soreness. 10.1016j.jpain.2013.07.019
A. Chiu, S. Liu, R. L. Carpenter, G. S. Kasman, J. E. Pollock and J. M. Neal. (1995). The effects of epinephrine on lidocaine spinal anesthesia: a cross-over study. 10.1097/00000539-199504000-00015
L. Petrini, K. Hennings, X. Li, F. Negro and L. Arendt-Nielsen. (2014). A human experimental model of episodic pain. 10.1016/j.ijpsycho.2014.07.013
M. Pajoutan, M. Ghesmaty Sangachin and L. A. Cavuoto. (2017). Central and peripheral fatigue development in the shoulder muscle with obesity during an isometric endurance task. 10.1186/s12891-017-1676-0
A. M. Drewes, K. D. Nielsen, C. Rasmussen, T. Arima, P. Svensson, P. Rössel and L. Arendt-Nielsen. (2000). The effects of controlled delta sleep deprivation on experimental pain in healthy subjects. 10.1300/J094v08n03_05

No callibration procedure /no pain stimuli

Frölich, M. A., Zhang, K., & Ness, T. J. (2013). Effect of sedation on pain perception. The Journal of the American Society of Anesthesiologists, 118(3), 611-621.
Chan, S., Ferreira, S., Ramos, B., Santos, M. J., Matos, L. C., & Machado, J. (2017). Assessment of acupuncture and moxibustion effects on the electrophysiological properties of the ulnar nerve: A nerve conduction study. Integrative Medicine International, 4, 198-207.
Bradshaw, D. H., Donaldson, G. W., Jacobson, R. C., Nakamura, Y., & Chapman, C. R. (2011). Individual differences in the effects of music engagement on responses to painful stimulation. The journal of pain, 12(12), 1262-1273.
Schnakenberg, M., Thomas, C., Schmelz, M., & Rukwied, R. (2020). Nerve growth factor sensitizes nociceptors to C-fibre selective supra-threshold electrical stimuli in human skin. European Journal of Pain, 25(2), 385–397. doi:10.1002/ejp.1678
Issa, R., Julien, M., Décary, E., Verdonck, O., Fortier, L.-P., Drolet, P., & Richebé, P. (2017). Evaluation of the analgesia nociception index (ANI) in healthy awake volunteers. Canadian Journal of Anesthesia/Journal Canadien D'anesthésie, 64(8), 828–835. doi:10.1007/s12630-017-0887-z
SANDIN, M., THÖRN, S.-E., DAHLQVIST, A., WATTWIL, L., AXELSSON, K., & WATTWIL, M. (2008). Effects of pain stimulation on bispectral index, heart rate and blood pressure at different minimal alveolar concentration values of sevoflurane. Acta Anaesthesiologica Scandinavica, 52(3), 420–426. doi:10.1111/j.1399-6576.2007.01569.x
VANCLEEF, L., & PETERS, M. (2006). Pain Catastrophizing, but not Injury/Illness Sensitivity or Anxiety Sensitivity, Enhances Attentional Interference by Pain. The Journal of Pain, 7(1), 23–30. doi:10.1016/j.jpain.2005.04.003 S
Li, Y., Li, W., Zhang, T., Zhang, J., Jin, Z., & Li, L. (2021). Probing the role of the right inferior frontal gyrus during Pain-Related empathy processing: Evidence from fMRI and TMS. Human brain mapping, 42(5), 1518-1531.
Müller MJ. Helplessness and perceived pain intensity: relations to cortisol concentrations after electrocutaneous stimulation in healthy young men. Biopsychosoc Med. 2011 Jun 30;5:8. doi: 10.1186/1751-0759-5-8. PMID: 21718526; PMCID: PMC3141369.
Schrooten, M. G. S., Karsdorp, P. A., & Vlaeyen, J. W. S. (2013). Pain catastrophizing moderates the effects of pain-contingent task interruptions. European Journal of Pain, 17(7), 1082–1092. doi:10.1002/j.1532-2149.2012.00276.x
Jess, G., Pogatzki-Zahn, E. M., Zahn, P. K., & Meyer-Frieem, C. H. (2016). Monitoring heart rate variability to assess experimentally induced pain using the analgesia nociception index. European Journal of Anaesthesiology, 33(2), 118–125. doi:10.1097/eja.0000000000000304
Lautenbacher, S., Prager, M., & Rollman, G. B. (2007). Pain additivity, diffuse noxious inhibitory controls, and attention: A functional measurement analysis. Somatosensory & Motor Research, 24(6), 189–201. doi:10.1080/08990220701637638
Bromm, B., & Seide, K. (1982). The influence of tilidine and prazepam on withdrawal reflex, skin resistance reaction and pain rating in man. Pain, 12(3), 247–258. doi:10.1016/0304-3959(82)90156-7
Müller, M. J., & Netter, P. (2000). Relationship of subjective helplessness and pain perception after electric skin stimuli. Stress Medicine, 16(2), 109-115.
Ashton, H., Golding, J. F., Marsh, V. R., & Thompson, J. W. (1984). Effects of transcutaneous electrical nerve stimulation and aspirin on late somatosensory evoked potentials in normal subjects. Pain, 18(4), 377–386. doi:10.1016/0304-3959(84)90050-2
Nordin, M., & Fagius, J. (1995). Effect of noxious stimulation on sympathetic vasoconstrictor outflow to human muscles. The Journal of physiology, 489(3), 885-894.
Reeves, J. L., Graff-Radford, S. B., & Shipman, D. (2004). The effects of transcutaneous electrical nerve stimulation on experimental pain and sympathetic nervous system response. Pain Medicine, 5(2), 150-161.
Skinner-Robertson, S., Mouksassi, M. S., & Varin, F. (2018). Evaluation of an Experimental Pain Model by Noncompartmental Analysis of Results from a Randomized Placebo Controlled Trial. Pain Physician, 21(4), 363-372.
Müller, M. J. (2013). Depressive attribution style and stressor uncontrollability increase perceived pain intensity after electrical skin stimuli in healthy young men. Pain Research and Management, 18(4), 203-206.
Svensson, P., Miles, T. S., McKay, D., & Ridding, M. C. (2003). Suppression of motor evoked potentials in a hand muscle following prolonged painful stimulation. European Journal of Pain, 7(1), 55–62. doi:10.1016/s1090-3801(02)00050-2
Robertson, S. S., Mouksassi, M. S., & Varin, F. (2019). Population pharmacokinetic/pharmacodynamic modeling of O-desmethyltramadol in young and elderly healthy volunteers. Drugs & Aging, 36(8), 747-758.
Van Damme, S., Crombez, G., Eccleston, C., & Goubert, L. (2004). Impaired disengagement from threatening cues of impending pain in a crossmodal cueing paradigm. European Journal of Pain, 8(3), 227-236.
Seki, T. (1978). Evaluation of effect of acetylsalicylic acid using electrical stimulation on the forefinger of healthy volunteers. British Journal of Clinical Pharmacology, 6(6), 521-524.
van Esdonk, M. J., Lindeman, I., Okkerse, P., de Kam, M. L., Groeneveld, G. J., & Stevens, J. (2018). Population Pharmacokinetic/Pharmacodynamic Analysis of Nociceptive Pain Models Following an Oral Pregabalin Dose Administration to Healthy Subjects. CPT: pharmacometrics & systems pharmacology, 7(9), 573-580.
Stacher, G., Bauer, P., Schneider, C., Winklehner, S., & Schmierer, G. (1982). Effects of a combination of oral naproxen sodium and codeine on experimentally induced pain. European Journal of Clinical Pharmacology, 21(6), 485–490. doi:10.1007/bf00542043
Gram, M., Graversen, C., Nielsen, A. K., Arendt-Nielsen, T., Mørch, C. D., Andresen, T., & Drewes, A. M. (2013). A novel approach to pharmaco-EEG for investigating analgesics: assessment of spectral indices in single-sweep evoked brain potentials. British Journal of Clinical Pharmacology, 76(6), 951–963. doi:10.1111/bcp.12120
van Esdonk, M. J., Lindeman, I., Okkerse, P., de Kam, M. L., Groeneveld, G. J., & Stevens, J. (2018). Population Pharmacokinetic/Pharmacodynamic Analysis of Nociceptive Pain Models Following an Oral Pregabalin Dose Administration to Healthy Subjects. CPT: pharmacometrics & systems pharmacology, 7(9), 573-580.
Bąbel, P. (2017). The influence of state and trait anxiety on the memory of pain. Pain Medicine, 18(12), 2340-2349.
Schwerdtfeger, A., & Heer, J. (2008). Second to fourth digit ratio (2D:4D) of the right hand is associated with nociception and augmenting-reducing. Personality and Individual Differences, 45(6), 493–497. doi:10.1016/j.paid.2008.05.027
Chipchase, L. S., Schabrun, S. M., & Hodges, P. W. (2011). Corticospinal Excitability is Dependent on the Parameters of Peripheral Electric Stimulation: A Preliminary Study. Archives of Physical Medicine and Rehabilitation, 92(9), 1423–1430. doi:10.1016/j.apmr.2011.01.011
Bräscher, A.-K., & Witthöft, M. (2019). Nocebo hyperalgesia induced by implicit conditioning. Journal of Behavior Therapy and Experimental Psychiatry. doi:10.1016/j.jbtep.2019.03.006
Van Ryckeghem, D. M. L., Crombez, G., Van Hulle, L., & Van Damme, S. (2012). Attentional bias towards pain-related information diminishes the efficacy of distraction. Pain, 153(12), 2345–2351. doi:10.1016/j.pain.2012.07.032
C. M. Klingner, R. Huonker, S. Flemming, C. Hasler, S. Brodoehl, C. Preul, H. Burmeister, A. Kastrup and O. W. Witte. (2011). Functional deactivations: Multiple ipsilateral brain areas engaged in the processing of somatosensory information. 10.1002/hbm.21006
K. Inui, T. D. Tran, Y. Qiu, X. Wang, M. Hoshiyama and R. Kakigi. (2003). A comparative magnetoencephalographic study of cortical activations evoked by noxious and innocuous somatosensory stimulations. 10.1016s0306-4522(03)00261-6
M. J. Müller. (2013). Depressive attribution style and stressor uncontrollability increase perceived pain intensity after electric skin stimuli in healthy young men. 10.1155/2013/263084
M. Hoshiyama and R. Kakigi. (2000). After-effect of transcutaneous electrical nerve stimulation (TENS) on pain-related evoked potentials and magnetic fields in normal subjects. 10.1016/S1388-2457(99)00299-0
H. Basedau, T. Nielsen, K. Asmussen, K. Gloss, J. Mehnert, R. H. Jensen and A. May. (2022). Experimental evidence of a functional relationship within the brainstem trigeminocervical complex in humans. 10.1097j.pain.0000000000002417
M. M. Yudina, G. G. Toropina, A. N. Lvov and U. Gieler. (2011). Innovative neurophysiological methods in itch research: Longlatency evoked potentials after electrical and thermal stimulation in patients with atopic dermatitis. 10.2340/00015555-1173
A. Yucel, A. Miyazawa, O. K. Andersen and L. Arendt-Nielsen. (2004). Comparison of hyperalgesia induced by capsaicin injection and controlled heat injury: Effect on temporal summation. 10.1080/0899022042000201263
W. Freund, A. P. Wunderlich, G. Stuber, F. Mayer, P. Steffen, M. Mentzel, F. Weber and B. Schmitz. (2010). Different activation of opercular and posterior cingulate cortex (PCC) in patients with complex regional pain syndrome (CRPS I) compared with healthy controls during perception of electrically induced pain: a functional MRI study. 10.1097AJP.0b013e3181cb4055
Hsu, F. Farahani and L. C. Parra. (2021). Cutaneous sensation of electrical stimulation waveforms. 10.1016j.brs.2021.04.008
W. C. Clark, J. D. Carroll and M. N. Janal. (2010). A novel method for the construction of verbal rating scales at the ratio scale level of measurement precision: The multidimensionalpsychophysical scaling procedure (M-PSP). 10.3758/APP.72.2.548
Baron, R., Baron, Y., Disbrow, E., & Roberts, T. P. (2000). Activation of the somatosensory cortex during Aβ-fiber mediated hyperalgesia: a MSI study. 10.1016/S0006-8993(00)02444-6
H. Haavik Taylor and B. A. Murphy. (2007). Altered cortical integration of dual somatosensory input following the cessation of a 20 min period of repetitive muscle activity. 10.1007/s00221-006-0755-5
G. Li, H. L. Liu, R. T. F. Cheung, Y. C. Hung, K. K. K. Wong, G. G. X. Shen, Q. Y. Ma and E. S. Yang. (2003). An fMRI study comparing brain activation between word generation and electrical stimulation of language-implicated acupoints. 10.1002/hbm.10098
A. Blenkinship, A. E. Benham, O. Tashani and M. I. Johnson. (2011). An investigation into the hypoalgesic effects of transcutaneous piezoelectric current on experimentally induced thermal stimuli in healthy participants. 10.1111j.1525-1403.2011.00349.x
S. Durand, B. Fromy, A. Humeau, D. Sigaudo-Roussel, J. L. Saumet and P. Abraham. (2002). Break excitation alone does not explain the delay and amplitude of anodal current-induced vasodilatation in human skin. 10.1113jphysiol.2002.022731
L. B. Macedo, A. M. Josué, P. H. Maia, A. E. Câmara and J. S. Brasileiro. (2015). Effect of burst TENS and conventional TENS combined with cryotherapy on pressure pain threshold: randomised, controlled, clinical trial. 10.1016j.physio.2014.07.004
J. C. Simons, E. Pierce, D. Diaz-Gil, S. A. Malviya, M. J. Meyer, F. P. Timm, J. B. Stokholm, C. E. Rosow, R. M.Kacmarek and M. Eikermann. (2016). Effects of Depth of Propofol and Sevoflurane Anesthesia on Upper Airway Collapsibility, Respiratory Genioglossus Activation, and Breathing in Healthy Volunteers. 10.1097aln.0000000000001225
M. Mücke, H. Cuhls, L. Radbruch, T. Weigl and R. Rolke. (2014). Evidence of heterosynaptic LTD in the human nociceptive system: superficial skin neuromodulation using a matrix electrode reduces deep pain sensitivity. 10.1371/journal.pone.0107718
J. F. Antognini, M. H. Buonocore, E. A. Disbrow and E. Carstens. (1997). Isoflurane anesthesia blunts cerebral responses to noxious and innocuous stimuli: A fMRI study. 10.1016/S0024-3205(97)00960-0
V. Kumari, D. H. ffytche, M. Das, G. D. Wilson, S. Goswami and T. Sharma . (2007). Neuroticism and brain responses to anticipatory fear . 10.1037/0735-7044.121.4.643;10.1037/0735-7044.121.4.643.supp(Supplemental)
A. Katafigioti, A. Paraskeva, G. Petropoulos, I. Siafaka and A. Fassoulaki. (2009). Pregnancy at term does not alter the responses to a mechanical and an electrical stimulus after skin EMLA application. https://pubmed.ncbi.nlm.nih.gov/19583074/
M. Yaman, D. Uluduz, O. Solak, G. Pay and M. E. Kiziltan. (2007). The cutaneous silent period in carpal tunnel syndrome. https://www.scopus.com/inwardrecord.uri?eid=2-s2.0-34547147512&partnerID=40&md5=d413b0621d2d8ecf17909e35a67044c1

M. Gram, C. Graversen, A. K. Nielsen, T. Arendt-Nielsen, C. D. Mørch, T. Andresen and A. M. Drewes. (2013). A novel approach to pharmaco-EEG for investigating analgesics: assessment of spectral indices in single-sweep evoked brain potentials. 10.1111/bcp.12120
J. Mpendo, G. Mutua, A. Nanvubya, O. Anzala, J. Nyombayire, E. Karita, L. Dally, D. Hannaman, M. Price, P. E. Fast, F. Priddy, H. C. Gelderblom and N. K. Hills. (2020). Acceptability and tolerability of repeated intramuscular electroporation of Multi-antigenic HIV (HIVMAG) DNA vaccine among healthy African participants in a phase 1 randomized controlled trial. 10.1371/journal.pone.0233151
R. Nemes, G. Nagy, G. S. Murphy, Logvinov, II, B. Fülesdi and J. R. Renew. (2020). Awake Volunteer Pain Scores During Neuromuscular Monitoring. 10.1213/ane.0000000000004326
Van Ryckeghem, Dimitri M. L.; Crombez, Geert; Van Hulle, Lore; Van Damme, Stefaan. (2012). Attentional bias towards pain-related information diminishes the efficacy of distraction. 10.1016/j.pain.2012.07.032
C. H. Meyer-Frießem, G. Jess, E. M. Pogatzki-Zahn and P. K. Zahn. (2017). Cerebral oxygenation for pain monitoring in adults is ineffective: A sequence-randomized, sham controlled study in volunteers. 10.1016j.sjpain.2017.05.001
A. Dickie, G. Tabasam, O. Tashani, P. Marchant and M. I. Johnson. (2009). A preliminary investigation into the effect of coffee on hypolagesia associated with transcutaneous electrical nerve stimulation. 10.1111j.1475-097X.2009.00869.x
T. D. Tran, D. Matre and K. L. Casey. (2008). An inhibitory interaction of human cortical responses to stimuli preferentially exciting Aδ or C fibers. 10.1016/j.neuroscience.2007.11.050
C. A. Miller, D. J. Parrott and P. R. Giancola. (2009). Agreeableness and alcohol-related aggression: the mediating effect of trait aggressivity. 10.1037/a0017727
R. Jonas, B. Namer, L. Stockinger, K. Chisholm, M. Schnakenberg, G. Landmann, M. Kucharczyk, C. Konrad, R. Schmidt, R. Carr, S. McMahon, M. Schmelz and R. Rukwied. (2018). Tuning in C-nociceptors to reveal mechanisms in chronic neuropathic pain. 10.1002/ana.25231
M. S. Buchsbaum, G. Muscettola and F. K. Goodwin. (1981). Urinary MHPG, stress response, personality factors and somatosensory evoked potentials in normal subjects and patients with major affective disorders. 10.1159000117853
M. S. Buchsbaum, G. C. Davis, D. Naber and D. Pickar. (1983). Pain enhances naloxone-induced hyperalgesia in humans as assessed by somatosensory evoked potentials. 10.1007/bf00427792
P. C. Duker, H. Douwenga, S. Joosten and T. Franken. (2002). Effects of single and repeated shock on perceived pain and startle response in healthy volunteers. 10.1016/s0891-4222(02)00119-1
P. Sommer, O. Kluschina, M. Schley, B. Namer, M. Schmelz and R. Rukwied. (2011). Electrically induced quantitative sudomotor axon reflex test in human volunteers. 10.1016/j.autneu.2010.09.004
S. Leis, M. Weber, M. Schmelz and F. Birklein. (2004). Facilitated neurogenic inflammation in unaffected limbs of patients with complex regional pain syndrome. 10.1016/j.neulet.2004.02.025
S. Alaid, I. Tyagi and M. Kornhuber. (2012). Facilitation and habituation of the startle reflex over the tonically active biceps brachii muscle contralateral to electrical stimuli. 10.1016/j.neulet.2012.08.025
M. F. Nolan, J. K. Hartsfield Jr, D. M. Witters and P. James Wason. (1993). Failure of transcutaneous electrical nerve stimulation in the conventional and burst modes to alter digital skin temperature. https://www.scopus.cominward/record.uri?eid=2-s2.0-0027399167&partnerID=40&md5=3e9b752b815393cf12f21d72d6f41692
J. L. Rhudy, J. S. Grimes and M. W. Meagher. (2004). Fear-Induced Hypoalgesia in Humans: Effects on Low Intensity Thermal Stimulation and Finger Temperature. 10.1016/j.jpain.2004.08.001
P. C. Duker, J. van den Bercken and M.-A. Foekens. (1999). Focusing versus distraction and the response to clinical electric shocks. 10.1016/S0005-7916(99)00025-7
D. Matre, M. B. Olsen, L. M. Jacobsen, T. Klein and J. Gjerstad. (2013). Induction of the perceptual correlate of human long-term potentiation (LTP) is associated with the 5-HTT genotype. 10.1016/j.brainres.2012.10.045
D. Algom, N. Raphaeli and L. Cohen-Raz . (1986). Integration of noxious stimulation across separate somatosensory communications systems: A functional theory of pain . 10.1037/0096-1523.12.1.92
R. Lütolf, S. U. Júlio, M. Schubert and M. Hubli. (2022). Intra-epidermal evoked potentials: A promising tool for spinal disorders?. 10.1016/j.neucli.2021.11.004
B. Murphy, N. Dawson and R. John Irwin. (2002). Intramuscular sensation in conscious human subjects: A qualitative and quantitative study utilizing signal detection theory methodology. 10.108008990220210000009099
Carvalho, M. French, A. Thibaut, W. Lima, M. Simis, J. Leite and F. Fregni. (2018). Median nerve stimulation induced motor learning in healthy adults: A study of timing of stimulation and type of learning. 10.1111ejn.13990
A. Gündüz, S. Ayas, M. Kofler, Ş. Aydın and M. E. Kızıltan. (2021). Modulation of the excitatory phase following the cutaneous silent period by vibration. 10.1007/s10072-020-04557-5
C. Z. Duval, Y. Goumon, V. Kemmel, J. Kornmeier, A. Dufour, O. Andlauer, P. Vidailhet, P. Poisbeau, E. Salvat, A. Muller, A. G. Mensah-Nyagan, C. Schmidt-Mutter and A. Giersch. (2016). Neurophysiological responses to unpleasant stimuli (acute electrical stimulations and emotional pictures) are increased in patients with schizophrenia. 10.1038/srep22542
T. Keller, M. Lawrence, A. Kuhn and M. Morari. (2006). New multi-channel transcutaneous electrical stimulation technology for rehabilitation. 10.1109/iembs.2006.259399
D. Kindgen-Milles and H. Holthusen. (1997). Nociception from blood vessels is independent of the sympathetic nervous system under physiological conditions in humans. 10.1016/s0014-2999(97)83025-7
G. Lichtner, R. Auksztulewicz, H. Velten, D. Mavrodis, M. Scheel, F. Blankenburg and F. von Dincklage. (2018). Nociceptive activation in spinal cord and brain persists during deep general anaesthesia. 10.1016/j.bja.2018.03.031
C. Geber, W. Magerl, R. Fondel, M. Fechir, R. Rolke, T. Vogt, R. D. Treede and F. Birklein. (2008). Numbness in clinical and experimental pain--a cross-sectional study exploring the mechanisms of reduced tactile function. 10.1016/j.pain.2008.03.006
P. Kokotis, M. Schmelz, A. E. Papagianni, T. Zambelis and N. Karandreas. (2013). Objective assessment of C-fiber function by electrically induced axon reflex flare in patients with axonal and demyelinating polyneuropathy. 10.1097/WNP.0b013e31829ddb97
A. Dufour, S. Guergova, T. Pebayle and P. Touzalin-Chretien. (2011). On the selective activation of unmyelinated C-fibers using sinusoidal electrical stimulation: An ERP study. 10.1016/j.clinph.2011.01.046
Y. Saitoh, K. Nakazawa, H. Toyooka and K. Amaha. (1995). Optimal stimulating current for train-of-four stimulation in conscious subjects. 10.1007/bf03011071
M. D. Larson, D. I. Sessler, M. Ozaki, J. McGuire and M. Schroeder. (1993). Pupillary assessment of sensory block level during combined epidural/general anesthesia. 10.1097/00000542-199307000-00009
Y. Okajima, N. Chino, E. Saitoh and A. Kimura. (1991). Recovery functions of somatosensory vertex potentials in man: interaction of evoked responses from right and left fingers. 10.1016/0168-5597(91)90135-K
W. Wei and J. Schoenen. (1994). Reduction of temporalis exteroceptive suppression by peripheral electrical stimulation in migraine and tension-type headaches. 10.1016/0304-3959(94)90018-3
R. Kakigi and H. Shibasaki. (1984). Scalp topography of mechanically and electrically evoked somatosensory potentials in man. 10.1016/0168-5597(84)90019-4
J. C. Scaife, J. Groves, R. W. Langley, C. M. Bradshaw and E. Szabadi. (2006). Sensitivity of late-latency auditory and somatosensory evoked potentials to threat of electric shock and the sedative drugs diazepam and diphenhydramine in human volunteers. 10.1177/0269881105059343
A. Fassoulaki, C. Sarantopoulos, G. Karabinis and C. Derveniotis. (1998). Skin application of isoflurane attenuates the responses to a mechanical and an electrical stimulation. 10.1007/bf03012455
N Marlowe. (1995). Somatosensory evoked potentials and headache: a further examination of the central theory. 10.1016/0022-3999(94)00072-D
Connelly, N. R., Silverman, D. G., O'Connor, T. Z., & Brull, S. J. (1990). Subjective responses to train-of-four and double burst stimulation in awake patients. 10.1213/00000539-199006000-00012
H. I. Ozisik, O. Kamisli, R. Karlidag, S. Kizkin and C. Ozcan. (2005). Sympathetic skin response in premenstrual syndrome. 10.1007/s10286-005-0281-8
B. Selçuk, M. Ersoz, M. Inanir, A. Kurtaran and M. Akyuz. (2006). Sympathetic skin responses in hemiplegic patients with and without complex regional pain syndrome. 10.4103/0028-3886.27153
Suswardana, S. Radiono, N. Chusniyati, Rosmelia and Y. W. Wirohadidjojo. (2008). The effect of 50% glycolic acid on the percutaneous absorption of eutectic mixture of local anesthetics (EMLA): a study of the electrofulguration-induced pain. 10.1111/j.1365-4632.2008.03476.x
H. V. Wilson, A. Jones, M. I. Johnson and P. Francis. (2019). The effect of inter-electrode distance on radial muscle displacement and contraction time of the biceps femoris, gastrocnemius medialis and biceps brachii, using tensiomyography in healthy participants. 10.1088/1361-6579/ab1cef
B. Murphy and N. Dawson. (2002). The effects of repetitive contractions and ischemia on the ability to discriminate intramuscular sensation. 10.1080/0899022021000009107
M. D. Weber, F. J. Servedio and W. R. Woodall. (1994). The effects of three modalities on delayed onset muscle soreness. 10.2519/jospt.1994.20.5.236
A. Y. Leung, J. Park, G. Schulteis, J. R. Duann and T. Yaksh. (2006). The electrophysiology of de qi sensations. 10.1089/acm.2006.12.743
T. Mittwoch, M. Weisenberg and M. Mikulincer. (1990). The influence of warning signal timing and cognitive preparation on the aversiveness of electric shock. 10.1016/0304-3959(90)91150-h
P. J. Conrod. (2006). The role of anxiety sensitivity in subjective and physiological responses to social and physical stressors. 10.1080/16506070600898587
R. Casale, C. Glynn and M. Buonocore. (1992). The role of ischaemia in the analgesia which follows Bier's block technique. 10.1016/0304-3959(92)90158-8
M. Breimhorst, S. Sandrock, M. Fechir, N. Hausenblas, C. Geber and F. Birklein. (2011). Do intensity ratings and skin conductance responses reliably discriminate between different stimulus intensities in experimentally induced pain?. 10.1016/j.jpain.2010.04.012
M. A. Frölich, K. Zhang and T. J. Ness. (2013). Effect of sedation on pain perception. 10.1097ALN.0b013e318281592d
M. Sandin, S. E. Thörn, A. Dahlqvist, L. Wattwil, K. Axelsson and M. Wattwil. (2008). Effects of pain stimulation on bispectral index, heart rate and blood pressure at different minimal alveolar concentration values of sevoflurane. 10.1111/j.1399-6576.2007.01569.x
T. Seki. (1978). Evaluation of effect of acetylsalicylic acid using electrical stimulation on the forefinger of healthy volunteers. 10.1111/j.1365-2125.1978.tb00876.x
S. Van Damme, G. Crombez, C. Eccleston and L. Goubert. (2004). Impaired disengagement from threatening cues of impending pain in a crossmodal cueing paradigm. 10.1016/j.ejpain.2003.08.005
D. H. Bradshaw, G. W. Donaldson, R. C. Jacobson, Y. Nakamura and C. R. Chapman. (2011). Individual differences in the effects of music engagement on responses to painful stimulation. 10.1016/j.jpain.2011.08.010
I. Skouteri, C. Staikou, C. Sarantopoulos, I. Siafaka and A. Fassoulaki. (2007). Local application of halothane, isoflurane or sevoflurane increases the response to an electrical stimulus in humans. https://pubmed.ncbi.nlm.nih.gov/18018837/
G. Jess, E. M. Pogatzki-Zahn, P. K. Zahn and C. H. Meyer-Frießem. (2016). Monitoring heart rate variability to assess experimentally induced pain using the analgesia nociception index. 10.1097/EJA.0000000000000304
L. M. G. Vancleef and M. L. Peters. (2006). Pain catastrophizing, but not injury/Illness sensitivity or anxiety sensitivity, enhances attentional interference by pain. 10.1016/j.jpain.2005.04.003
A. Sellgren Engskov, I. Lejbman and J. Åkeson. (2021). Randomized cross-over evaluation of investigator gender on pain thresholds in healthy volunteers. 10.3205/000301
Schwerdtfeger and J. Heer. (2008). Second to fourth digit ratio (2d:4d) of the right hand is associated with nociception and augmenting-reducing. 10.1016/j.paid.2008.05.027
P. Bąbel. (2017). The influence of state and trait anxiety on the memory of pain. 10.1093/pm/pnw354
G. Crombez, C. Eccleston, A. Van den Broeck, B. Van Houdenhove and L. Goubert. (2002). The effects of catastrophic thinking about pain on attentional interference by pain: No mediation of negative affectivity in healthy volunteers and in patients with low back pain. 10.1155/2002/576792
J. L. Reeves Ii, S. B. Radford-Graff and D. Shipman. (2004). The effects of transcutaneous electrical nerve stimulation on experimental pain and sympathetic nervous system response. 10.1111/j.1526-4637.2004.04027.x
A. Eken, M. Kara, B. Baskak, A. Baltaci and D. Gökçay. (2018). Differential efficiency of transcutaneous electrical nerve stimulation in dominant versus nondominant hands in fibromyalgia: Placebo-controlled functional near-infrared spectroscopy study. 10.1117/1.NPh.5.1.011005
S. Van Damme, G. Crombez and C. Eccleston. (2004). Disengagement from pain: the role of catastrophic thinking about pain. 10.1016/j.pain.2003.09.023
J. S. Grimes, S. K. Creech, E. E. Young, E. G. Vichaya and M. W. Meagher. (2009). Distraction speeds the decay of shock-induced hypoalgesia: Evidence for the contribution of memory systems in affective pain modulation. 10.1016j.jpain.2008.09.005
E. L. Nussbaum and S. Gabison. (1998). Rebox effect on exercise-induced acute inflammation in human muscle. 10.1016/s0003-9993(98)90272-6
Maryam Zoghi 1, Shapour Jaberzadeh. (2002). Effects of High Voltage Electro-auriculotherapy on Experimental Pain Threshold. 10.1016/S0031-9406(05)60108-3
Roser, F., Ebner, F. H., Liebsch, M., Dietz, K., & Tatagiba, M. (2008). A new concept in the electrophysiological evaluation of syringomyelia. 10.3171/SPI/2008/8/6/517
G. van Amerongen, P. Siebenga, M. L. de Kam, J. L. Hay and G. J. Groeneveld. (2018). Effect profile of paracetamol, Δ9-THC and promethazine using an evoked pain test battery in healthy subjects. 10.1002/ejp.1222
B. A. Stuck, J. Baja, F. Lenz, R. M. Herr and C. Heiser. (2011). Co-stimulation with an olfactory stimulus increases arousal responses to trigeminal stimulation. 10.1016/j.neuroscience.2011.01.009
M. G. S. Schrooten, P. A. Karsdorp and J. W. S. Vlaeyen. (2013). Pain catastrophizing moderates the effects of pain-contingent task interruptions. 10.1002/j.1532-2149.2012.00276.x
M. Schnakenberg, C. Thomas, M. Schmelz and R. Rukwied. (2021). Nerve growth factor sensitizes nociceptors to C-fibre selective supra-threshold electrical stimuli in human skin. 10.1002/ejp.1678
K. Tripanpitak, W. Viriyavit, S. Y. Huang and W. Yu. (2020). Classification of pain event related potential for evaluation of pain perception induced by electrical stimulation. 10.3390/s20051491
S. J. Jones. (1981). An 'interference' approach to the study of somatosensory evoked potentials in man. 10.10160013-4694(81)91427-9
S. Nagi, A. G. Marshall, A. Makdani, E. Jarocka, J. Liljencrantz, M. Ridderström, S. Shaikh, F. O'Neill, D. Saade, S.Donkervoort, A. Reghan Foley, J. Minde, M. Trulsson, J. Cole, C. G. Bönnemann, A. T. Chesler, M. Catherine Bushnell, F. McGlone and H. Olausson. (2019). An ultrafast system for signaling mechanical pain in human skin. 10.1126/sciadv.aaw1297
Ninomiya, Yotaro; Kitamura, Yoshihiro; Yamamoto, Shin; Okamoto, Motoi; Oka, Hisao; Yamada, Norihito; Kuroda, Shigetoshi. (2001). Analysis of pain-related somatosensory evoked magnetic fields using the MUSIC (multiple signal classification) algorithm for magnetoencephalography. 10.1097/00001756-200106130-00029
Sabatini, Elisabetta; Della Penna, Stefania; Franciotti, Raffaella; Ferretti, Antonio; Zoccolotti, Pierluigi; Rossini, Paolo M.; Romani, Gian Luca; Gainotti, Guido. (2009). Brain structures activated by overt and covert emotional visual stimuli. 10.1016/j.brainresbull.2009.03.001
F. Henrich, W. Magerl, T. Klein, W. Greffrath and R. D. Treede. (2015). Capsaicin-sensitive C- and A-fibre nociceptors control long-term potentiation-like pain amplification in humans. 10.1093/brain/awv108
B. Frøkjær, E. Søfteland, C. Graversen, G. Dimcevski, L. L. Egsgaard, L. Arendt-Nielsen and A. M.Drewes. (2009). Central processing of gut pain in diabetic patients with gastrointestinal symptoms. 10.2337dc09-0324
Stowell, Hilton. (1978). Cerebral slow waves related to the perception of pain in man. 10.1016/0361-9230(77)90021-1
L. K. Lee, H. J. Jeon, Y. D. Choi, B. Kim and J. Kim. (2013). Change in the interferential current therapy - induced sensory threshold on the bodies of elderly people. 10.1007/s13530-013-0154-9
Waschulewski-Floruß, W. Miltner, S. Brody and C. Braun. (1994). Classical conditioning of pain responses. 10.3109/00207459408986042
M. L. Kukushkin, A. V. Syrovegin, A. V. Gnezdilov, L. A. Medvedeva and O. I. Zagorul'ko. (2003). Heterotopic nociceptive EMG-reactions in M. masseter. 10.1023/A:1023425308139
Kowalewski, J. C. Kattenstroth, T. Kalisch and H. R. Dinse. (2012). Improved acuity and dexterity but unchanged touch and pain thresholds following repetitive sensory stimulation of the fingers. 10.1155/2012/974504
M. Kofler. (2004). Influence of transcutaneous electrical nerve stimulation on cutaneous silent periods in humans. 10.1016/j.neulet.2004.02.035
H. Mochizuki, K. Inui, K. Yamashiro, N. Ootsuru and R. Kakigi. (2008). Itching-related somatosensory evoked potentials. 10.1016/j.pain.2008.02.017
Nakanishi, Y. Shimada and Y. Toyokura. (1974). Somatosensory evoked responses to mechanical stimulation in normal subjects and in patients with neurological disorders. 10.1016/0022-510X(74)90173-7
A. Nicotra, H. D. Critchley, C. J. Mathias and R. J. Dolan. (2006). Emotional and autonomic consequences of spinal cord injury explored using functional brain imaging. 10.1093/brain/awh699
R. Kakigi and H. Shibasaki. (1991). Estimation of conduction velocity of the spino-thalamic tract in man. 10.10160168-5597(91)90041-U
T. Bocci, R. Ferrucci, D. Barloscio, L. Parenti, F. Cortese, A. Priori and F. Sartucci. (2018). Cerebellar direct current stimulation modulates hand blink reflex: implications for defensive behavior in humans. 10.14814/phy2.13471
M. A. Farrar, M. J. Lee, J. Howells, P. I. Andrews and C. S. Y. Lin. (2017). Burning pain: Axonal dysfunction in erythromelalgia. 10.1097/j.pain.0000000000000856
Notebaert, Lies; Crombez, Geert; Vogt, Julia; De Houwer, Jan; Van Damme, Stefaan; Theeuwes, Jan. (2011). Attempts to control pain prioritize attention towards signals of pain: An experimental study. 10.1016/j.pain.2011.01.020
D. T. W. Yu and A. Y. M. Jones. (2014). Are physiological changes experienced by healthy subjects during acu-TENS associated with acupuncture point sensations?. 10.1136/acupmed-2013-010428
C. Kraus, M. Klöbl, M. Tik, B. Auer, T. Vanicek, N. Geissberger, D. M. Pfabigan, A. Hahn, M. Woletz, K. Paul, A. Komorowski, S. Kasper, C. Windischberger, C. Lamm and R. Lanzenberger. (2019). The pulvinar nucleus and antidepressant treatment: Dynamic modeling of antidepressant response and remission with ultra-high field functional MRI. 10.1038/s41380-017-0009-x
A. Barton. (2019). Veinplicity versus heat treatment for vein dilation: A randomised cross-over study. 10.11771129729818818962
B. Bromm and K. Seide. (1982). The influence of tilidine and prazepam on withdrawal reflex, skin resistance reaction and pain rating in man. 10.1016/0304-3959(82)90156-7
Washington, L. L., Gibson, S. J., & Helme, R. D. (2000). Age-related differences in the endogenous analgesic response to repeated cold water immersion in human volunteers. 10.1016/S0304-3959(00)00352-3
T. Cayrol, J. Lebleu, A. Mouraux, N. Roussel, L. Pitance and E. N. van den Broeke. (2020). Within- and between-session reliability of secondary hyperalgesia induced by electrical high-frequency stimulation. 10.1002ejp.1613
H. J. Nilsson and J. Schouenborg. (1999). Differential inhibitory effect on human nociceptive skin senses induced by local stimulation of thin cutaneous fibers. 10.1016/s0304-3959(98)00205-x
Y. Cooper, C. J. Vierck and D. C. Yeomans. (1986). Selective reduction of second pain sensations by systemic morphine in humans. 10.1016/0304-3959(86)90030-8
J. S. Walker, J. F. Arroyo, T. Nguyen and R. O. Day. (1993). Analgesic efficacy of non-steroidal anti-inflammatory drugs in experimental pain in humans. 10.1111/j.1365-2125.1993.tb00390.x
M. J. Müller. (2011). Helplessness and perceived pain intensity: Relations to cortisol concentrations after electrocutaneous stimulation in healthy young men. 10.1186/1751-0759-5-8
C. H. Cheng, M. Y. Lin and S. H. Yang. (2018). Age effect on automatic inhibitory function of the somatosensory and motor cortex: An MEG study. 10.3389/fnagi.2018.00053
K. Takekuma. (2000). Age and Gender Differences in Skin Sensory Threshold Assessed by Current Perception in Community-Dwelling Japanese. 10.2188/jea.10.1sup_33
R. Hugosdottir, C. D. Mørch, C. K. Jørgensen, C. W. Nielsen, M. V. Olsen, M. J. Pedersen and J.Tigerholm. (2019). Altered excitability of small cutaneous nerve fibers during cooling assessed with the perception threshold tracking technique. 10.1186/s12868-019-0527-3
C. H. Cheng, P. Y. S. Chan, S. Baillet and Y. Y. Lin. (2015). Age-related reduced somatosensory gating is associated with altered alpha frequency desynchronization. 10.1155/2015/302878
M. A. Wittenberg, M. Morr, A. Schnitzler and J. Lange. (2019). 10 Hz tACS over somatosensory cortex does not modulate supra-threshold tactile temporal discrimination in humans. 10.3389/fnins.2019.00311
D. Serrano-Muñoz, J. Avendaño-Coy, C. Simón-Martínez, J. Taylor and J. Gómez-Soriano. (2020). 20-kHz alternating current stimulation: effects on motor and somatosensory thresholds. 10.1186/s12984-020-00661-x
M. I. Johnson and G. Tabasam. (2003). A single-blind investigation into the hypoalgesic effects of different swing patterns of interferential currents on cold-induced pain in healthy volunteers. 10.1053/apmr.2003.50005
R. A. Blauenfeldt, S. S. Olesen, J. B. Hansen, C. Graversen and A. M. Drewes. (2010). Abnormal brain processing in hepatic encephalopathy: Evidence of cerebral reorganization?. 10.1097/MEG.0b013e32833d4717
N. Biggs, D. M. Walsh and M. I. Johnson. (2012). A comparison of the hypoalgesic effects of transcutaneous electrical nerve stimulation (TENS) and non-invasive interactive neurostimulation (InterX(®)) on experimentally induced blunt pressure pain using healthy human volunteers. 10.1111/j.1525-1403.2011.00394.x
E. Eek, L. W. Holmqvist and D. K. Sommerfeld. (2012). Adult norms of the perceptual threshold of touch (PTT) in the hands and feet in relation to age, gender, and right and left side using transcutaneous electrical nerve stimulation. 10.3109/09593985.2011.629021
C. C. Chen and M. I. Johnson. (2010). A comparison of transcutaneous electrical nerve stimulation (TENS) at 3 and 80 pulses per second on cold-pressor pain in healthy human participants. 10.1111/j.1475-097X.2010.00936.x
A. R. Ward, S. Lucas-Toumbourou and B. McCarthy. (2009). A comparison of the analgesic efficacy of medium-frequency alternating current and TENS. 10.1016/j.physio.2009.06.005
M. A. Pantaleão, M. F. Laurino, N. L. Gallego, C. M. Cabral, B. Rakel, C. Vance, K. A. Sluka, D. M. Walsh and R. E. Liebano. (2011). Adjusting pulse amplitude during transcutaneous electrical nerve stimulation (TENS) application produces greater hypoalgesia. 10.1016/j.jpain.2010.11.001
Ren, J., Xiang, J., Chen, Y., Li, F., Wu, T., & Shi, J. . (2019). Abnormal functional connectivity under somatosensory stimulation in migraine: a multi-frequency magnetoencephalography study. 10.1186/s10194-019-0958-3
Leong, G. W., Lauschke, J., Rutowski, S. B., & Waite, P. M. (2010). Age, gender, and side differences of cutaneous electrical perceptual threshold testing in an able-bodied population. 10.1080/10790268.2010.11689702
M. I. Johnson and G. Tabasam. (2002). A single-blind placebo-controlled investigation into the analgesic effects of interferential currents on experimentally induced ischaemic pain in healthy subjects. 10.1046j.1475-097x.2002.00416.x
M. I. Johnson and G. Tabasam. (1999). A double blind placebo controlled investigation into the analgesic effects of inferential currents (IFC) and transcutaneous electrical nerve stimulation (TENS) on cold-induced pain in healthy subjects. 10.1080/095939899307630
İ. Çıtak Karakaya, F. Güney Ö, Y. Aydın and M. G. Karakaya. (2014). Effects of thermal agents on electrical sensory threshold and current tolerance when applied prior to neuromuscular electrical stimulation. 10.3233bmr-130435
C. Perchet, M. Frot, A. Charmarty, C. Flores, S. Mazza, M. Magnin and L. Garcia-Larrea. (2012). Do we activate specifically somatosensory thin fibres with the concentric planar electrode? A scalp and intracranial EEG study. 10.1016/j.pain.2012.03.004
Y. Kuwahara, R. Tsukahara, S. Iwase, Y. Shimizu, N. Nishimura, J. Sugenoya and M. Sato. (2015). Arousal electrical stimuli evoke sudomotor activity related to P300, and skin vasoconstrictor activity related to N140 in humans. 10.1016/j.clinph.2014.08.018
B. A. Murphy, H. H. Taylor, S. A. Wilson, J. A. Knight, K. M. Mathers and S. Schug. (2003). Changes in median nerve somatosensory transmission and motor output following transient deafferentation of the radial nerve in humans. 10.1016/S1388-2457(03)00131-7
T. Wasaka, T. Kida, H. Nakata, K. Akatsuka and R. Kakigi. (2007). Characteristics of sensori-motor interaction in the primary and secondary somatosensory cortices in humans: A magnetoencephalography study. 10.1016j.neuroscience.2007.07.040
A. Kanai, A. Suzuki and H. Okamoto. (2010). Comparison of cutaneous anesthetic effect of 8% lidocaine spray with lidocaine patch using current perception threshold test. 10.1111/j.1526-4637.2009.00790.x
R. P. Francis, P. R. Marchant and M. I. Johnson. (2011). Comparison of post-treatment effects of conventional and acupuncture-like transcutaneous electrical nerve stimulation (TENS): A randomised placebo-controlled study using cold-induced pain and healthy human participants. 10.3109/09593985.2010.551803
W. T. Chen, F. J. Hsiao, Y. C. Ko, H. Y. Liu, P. N. Wang, J. L. Fuh, Y. Y. Lin and S. J. Wang. (2018). Comparison of somatosensory cortex excitability between migraine and "strict-criteria" tension-type headache: A magnetoencephalographic study. 10.1097/j.pain.0000000000001151
A. H. Y. Astokorki and A. R. Mauger. (2017). Transcutaneous electrical nerve stimulation reduces exercise-induced perceived pain and improves endurance exercise performance. 10.1007/s00421-016-3532-6
Buglewicz-Przewoźnik, E., Adamczyk, W. M., & Bąbel, P. (2022). Is Pain Contagious? Innocuous Stimulation Can be Transformed Into the Pain Experience by Observational Learning. 10.1016/j.jpain.2022.07.015
M. Buonocore, N. Camuzzini, A. Dall'Angelo, S. Mandrini and E. Dalla Toffola. (2015). Contralateral antalgic effect of high-frequency transcutaneous peripheral nerve stimulation. 10.1016/j.pmrj.2014.06.012
R. P. Francis, P. Marchant and M. I. Johnson. (2011). Conventional versus acupuncture-like transcutaneous electrical nerve stimulation on cold-induced pain in healthy human participants: effects during stimulation. 10.1111j.1475-097X.2011.01025.x
A. Blickenstorfer, R. Kleiser, T. Keller, B. Keisker, M. Meyer, R. Riener and S. Kollias. (2009). Cortical and subcortical correlates of functional electrical stimulation of wrist extensor and flexor muscles revealed by fMRI. 10.1002hbm.20559
P. J. Theuvenet, B. W. van Dijk, M. J. Peters, J. M. van Ree, F. L. Lopes da Silva and A. C. Chen. (2006). Cortical characterization and inter-dipole distance between unilateral median versus ulnar nerve stimulation of both hands in MEG. 10.1007/s10548-006-0010-1
C. Dockstader, D. Cheyne and R. Tannock. (2010). Cortical dynamics of selective attention to somatosensory events. 10.1016/j.neuroimage.2009.09.035
A. Mouraux and L. Plaghki. (2007). Cortical interactions and integration of nociceptive and non-nociceptive somatosensory inputs in humans. 10.1016/j.neuroscience.2007.08.035
A. Kudoh, H. Katagai and T. Takazawa. (2003). Current perception thresholds of patients with long-term administration of maprotiline. 10.1055/s-2003-39040
S. Durand, B. Fromy, P. Bouyé, J. L. Saumet and P. Abraham. (2002). Current-induced vasodilation during water iontophoresis (5 min, 0.10 mA) is delayed from current onset and involves aspirin sensitive mechanisms. 10.1159000048994
A. Priori, C. Cinnante, A. Pesenti, A. Gallanti, A. Cappellari, G. Scarlato and S. Barbieri. (2001). Decreased EMG inhibition following electrical stimulation over muscle tendons in myopathies. 10.1016/s1388-2457(01)00628-9
K. Haß, N. Bak, G. R. Szycik, B. Y. Glenthøj and B. Oranje. (2017). Deficient prepulse inhibition of the startle reflex in schizophrenia using a cross-modal paradigm. 10.1016/j.biopsycho.2017.07.016
Y. Y. Lin, Y. H. Shih, J. T. Chen, J. C. Hsieh, T. C. Yeh, K. K. Liao, C. D. Kao, K. P. Lin, Z. A. Wu and L. T.Ho. (2003). Differential effects of stimulus intensity on peripheral and neuromagnetic cortical responses to median nerve stimulation. 10.1016/S1053-8119(03)00387-2
C. C. Chen and M. I. Johnson. (2011). Differential frequency effects of strong nonpainful transcutaneous electrical nerve stimulation on experimentally induced ischemic pain in healthy human participants. 10.1097AJP.0b013e318208c926
Y. Y. Lin, W. T. Chen, K. K. Liao, T. C. Yeh, Z. A. Wu, L. T. Ho and L. S. Lee. (2005). Differential generators for N20m and P35m responses to median nerve stimulation. 10.1016/j.neuroimage.2004.12.047
K. Malejko, R. C. Brown, P. L. Plener, M. Bonenberger, H. Graf and B. Abler. (2021). Differential neural processing of unpleasant sensory stimulation in patients with major depression. 10.1007/s00406-020-01123-0
K. Kalogianni, A. Daffertshofer, F. C. T. van der Helm, A. C. Schouten, J. C. de Munck, G. Kwakkel, C. G. M. Meskers,E. H. van Wegen, A. S. Andringa, D. Hoevenaars, C. Winters, S. Zandvliet, T. Solis-Escalante, M. P. Vlaar, L.Filatova, J. P. A. Dewald and J. Yao. (2018). Disentangling Somatosensory Evoked Potentials of the Fingers: Limitations and Clinical Potential. 10.1007/s10548-017-0617-4
M. L. Peters, J. W. S. Vlaeyen and C. Van Drunen. (2000). Do fibromyalgia patients display hypervigilance for innocuous somatosensory stimuli? Application of a body scanning reaction time paradigm. 10.1016S0304-3959(00)00259-1
K. Yamashiro, D. Sato, H. Onishi, K. Sugawara, S. Nakazawa, S. Kameyama and A. Maruyama. (2015). Effect of Changes in Stimulus Site on Activation of the Posterior Parietal Cortex. 10.1007/s10548-014-0378-2
A. Faghani Jadidi, A. J. T. Stevenson, A. A. Zarei, W. Jensen and R. Lontis. (2022). Effect of Modulated TENS on Corticospinal Excitability in Healthy Subjects. 10.1016/j.neuroscience.2022.01.004
E. Urasaki, S. I. Wada, H. Yasukouchi and A. Yokota. (1998). Effect of transcutaneous electrical nerve stimulation (TENS) on central nervous system amplification of somatosensory input. 10.1007/s004150050194
C. R. Jutzeler, A. Curt and J. L. K. Kramer. (2015). Effectiveness of high-frequency electrical stimulation following sensitization with capsaicin. 10.1016/j.jpain.2015.03.005
P. Barlas, S. L. Ting, L. S. Chesterton, P. W. Jones and J. Sim. (2006). Effects of intensity of electroacupuncture upon experimental pain in healthy human volunteers: a randomized, double-blind, placebo-controlled study. 10.1016j.pain.2006.01.012
L. Lazarou, A. Kitsios, I. Lazarou, E. Sikaras and A. Trampas. (2009). Effects of intensity of Transcutaneous Electrical Nerve Stimulation (TENS) on pressure pain threshold and blood pressure in healthy humans: A randomized, double-blind, placebo-controlled trial. 10.1097/AJP.0b013e3181a7ece3
V. I. Spoormaker, M. S. Schröter, K. C. Andrade, M. Dresler, S. A. Kiem, R. Goya-Maldonado, T. C. Wetter, F. Holsboer, P. G. Sämann and M. Czisch. (2012). Effects of rapid eye movement sleep deprivation on fear extinction recall and prediction error signaling. 10.1002/hbm.21369
L. S. Claydon, L. S. Chesterton, P. Barlas and J. Sim. (2008). Effects of simultaneous dual-site TENS stimulation on experimental pain. 10.1016/j.ejpain.2007.10.014
L. S. Chesterton, N. E. Foster, C. C. Wright, G. D. Baxter and P. Barlas. (2003). Effects of TENS frequency, intensity and stimulation site parameter manipulation on pressure pain thresholds in healthy human subjects. 10.1016S0304-3959(03)00292-6
C. C. Chen, W. B. Huang, Y. F. Chuang, A. C. W. Huang and Y. J. Chang. (2015). Effects of transcutaneous electrical nerve stimulation on experimental blunt pressure pain in healthy participants in randomized controlled trial: Pulse frequency and pad size. 10.1007/s40846-015-0062-3
S. Tanaka, J. Gomez-Tames, T. Wasaka, K. Inui, S. Ueno and A. Hirata. (2020). Electrical Characterisation of Aδ-Fibres Based on Human in vivo Electrostimulation Threshold. 10.3389/fnins.2020.588056
A. Hadzic, J. D. Vloka, R. E. Claudio, N. Hadzic, D. M. Thys and A. C. Santos. (2004). Electrical nerve localization: Effects of cutaneous electrode placement and duration of the stimulus on motor response. 10.109700000542-200406000-00027
A. Ikoma, H. Handwerker, Y. Miyachi and M. Schmelz. (2005). Electrically evoked itch in humans. 10.1016j.pain.2004.10.003

Y. Stussi, G. Pourtois and D. Sander . (2018). Enhanced Pavlovian aversive conditioning to positive emotional stimuli . 10.1037/xge0000424;10.1037/xge0000424.supp(Supplemental)
D. Lenoir, W. Willaert, K. Ickmans, J. Nijs, B. Cagnie, M. Meeus, K. Vonck, J. Delbeke, R. D. Pauw and I.Coppieters. (2022). Event-Related Potentials Following Cutaneous Electrical Stimulation in Patients With Chronic Whiplash-Associated Disorders. https://www.scopus.com/inwardrecord.uri?eid=2-s2.0-85131248547&partnerID=40&md5=75eb7b0039f1689f01482f3d704aab22
I. M. Tarkka, S. Micheloyannis and D. S. Stokić. (1996). Generators for human P300 elicited by somatosensory stimuli using multiple dipole source analysis. 10.1016/0306-4522(96)00287-4
C. M. Klingner, I. Nenadic, C. Hasler, S. Brodoehl and O. W. Witte. (2011). Habituation within the somatosensory processing hierarchy. 10.1016/j.bbr.2011.07.053
R. C. G. Helmich, T. Bäumer, H. R. Siebner, B. R. Bloem and A. Münchau. (2005). Hemispheric asymmetry and somatotopy of afferent inhibition in healthy humans. 10.1007/s00221-005-0014-1
C. E. Zyloney, K. Jensen, G. Polich, R. E. Loiotile, A. Cheetham, P. S. LaViolette, P. Tu, T. J. Kaptchuk, R. L. Gollub and J. Kong. (2010). Imaging the functional connectivity of the Periaqueductal Gray during genuine and sham electroacupuncture treatment. 10.1186/1744-8069-6-80
A. Nardone and M. Schieppati. (1989). Influences of transcutaneous electrical stimulation of cutaneous and mixed nerves on subcortical and cortical somatosensory evoked potentials. 10.1016/0168-5597(89)90048-8
A. Lozza, J. Schoenen and P. J. Delwaide. (1997). Inhibition of the blink reflex R2 component after supraorbital and index finger stimulations is reduced in cluster headache: an indication for both segmental and suprasegmental dysfunction?. 10.1016/s0304-3959(97)03342-3
G. Coppola, L. Di Clemente, A. Fumal, D. Magis, V. De Pasqua, F. Pierelli and J. Schoenen. (2007). Inhibition of the nociceptive R2 blink reflex after supraorbital or index finger stimulation is normal in migraine without aura between attacks. 10.1111/j.1468-2982.2007.01323.x
K. Saito, N. Otsuru, Y. Inukai, S. Kojima, S. Miyaguchi, S. Tsuiki, R. Sasaki and H. Onishi. (2018). Inhibitory Mechanisms in Primary Somatosensory Cortex Mediate the Effects of Peripheral Electrical Stimulation on Tactile Spatial Discrimination. 10.1016/j.neuroscience.2018.05.032
D. Ristic and J. Ellrich. (2014). Innocuous peripheral nerve stimulation shifts stimulus-response function of painful laser stimulation in man. 10.1111/ner.12133
C. Kavcıoğlu, F. Bublatzky, A. Pittig and G. W. Alpers . (2021). Instructed threat enhances threat perception in faces . 10.1037/emo0000708
S. D. Iftime-Nielsen, M. S. Christensen, R. J. Vingborg, T. Sinkjær, A. Roepstorff and M. J. Grey. (2012). Interaction of electrical stimulation and voluntary hand movement in SII and the cerebellum during simulated therapeutic functional electrical stimulation in healthy adults. 10.1002/hbm.21191
K. A. Kaczmarek, M. E. Tyler, U. O. Okpara and S. J. Haase. (2017). Interaction of Perceived Frequency and Intensity in Fingertip Electrotactile Stimulation: Dissimilarity Ratings and Multidimensional Scaling. 10.1109TNSRE.2017.2702628
S. N. Garfinkel, C. D. Gould van Praag, M. Engels, D. Watson, M. Silva, S. L. Evans, T. Duka and H. D. Critchley . (2021). Interoceptive cardiac signals selectively enhance fear memories . 10.1037/xge0000967
M. I. Lai, L. L. Pan, M. W. Tsai, Y. F. Shih, S. H. Wei and L. W. Chou. (2016). Investigating the effects of peripheral electrical stimulation on corticomuscular functional connectivity stroke survivors. 10.108010749357.2015.1122264
K. Cuypers, O. Levin, H. Thijs, S. P. Swinnen and R. L. J. Meesen. (2010). Long-term TENS treatment improves tactile sensitivity in MS patients. 10.1177/1545968309356301
R. J. Schneider. (1981). Low temperature painful stimulus alters brain wave pattern of transcutaneous electrical stimulus. 10.1016/0024-3205(81)90453-7
R. J. Schneider. (1982). Low temperature stimulation augments early wave component of somatosensory evoked response in humans and Macaca mulatta monkeys. 10.1016/0304-3959(82)90068-9
G. Wik, T. Elbert, M. Fredrikson and M. Hoke. (1996). Magnetic imaging in human classical conditioning. 10.109700001756-199602290-00014
Desmedt JE, Tomberg C. (1989). Mapping early somatosensory evoked potentials in selective attention: critical evaluation of control conditions used for titrating by difference the cognitive P30, P40, P100 and N140. 10.1016/0168-5597(89)90001-4
Lindström and A. Olsson . (2015). Mechanisms of social avoidance learning can explain the emergence of adaptive and arbitrary behavioral traditions in humans . 10.1037/xge0000071;10.1037/xge0000071.supp(Supplemental)
Rossi S, Pasqualetti P, Tecchio F, Sabato A, Rossini PM. (1998). Modulation of corticospinal output to human hand muscles following deprivation of sensory feedback. 10.1006/nimg.1998.0352
H. Kumru, S. Albu, J. Rothwell, D. Leon, C. Flores, E. Opisso, J. M. Tormos and J. Valls-Sole. (2017). Modulation of motor cortex excitability by paired peripheral and transcranial magnetic stimulation. 10.1016j.clinph.2017.06.041
V. Donadio, R. Liguori, M. Elam, T. Karlsson, M. P. Giannoccaro, G. Pegenius, F. Giambattistelli and B. G. Wallin. (2012). Muscle sympathetic response to arousal predicts neurovascular reactivity during mental stress. 10.1113/jphysiol.2012.228981
E. N. van den Broeke, C. M. van Rijn, J. A. Biurrun Manresa, O. K. Andersen, L. Arendt-Nielsen and O. H. Wilder-Smith. (2010). Neurophysiological correlates of nociceptive heterosynaptic long-term potentiation in humans. 10.1152/jn.00979.2009
M. F. van de Sand, M. M. Menz, C. Sprenger and C. Büchel. (2018). Nocebo-induced modulation of cerebral itch processing—An fMRI study. 10.1016/j.neuroimage.2017.10.056
Deriu, M. Milia, G. Sau, M. V. Podda, E. Ortu, G. Chessa, I. Aiello and E. Tolu. (2002). Non-nociceptive upper limb afferents modulate masseter muscle EMG activity in man. 10.1007/s00221-001-0997-1
P. Iovino, F. Tremolaterra, D. Consalvo, F. Sabbatini, G. Mazzacca and C. Ciacci. (2006). Perception of electrocutaneous stimuli in irritable bowel syndrome. 10.1111/j.1572-0241.2006.00414.x
C. M. Klingner, C. Hasler, S. Brodoehl, H. Axer and O. W. Witte. (2013). Perceptual plasticity is mediated by connectivity changes of the medial thalamic nucleus. 10.1002/hbm.22074
J. Ellrich and S. Lamp. (2005). Peripheral nerve stimulation inhibits nociceptive processing: An electrophysiological study in healthy volunteers. 10.1111/j.1525-1403.2005.00029.x
C. Cottone, L. Tomasevic, C. Porcaro, G. Filligoi and F. Tecchio. (2013). Physiological aging impacts the hemispheric balances of resting state primary somatosensory activities. 10.1007/s10548-012-0240-3
M. Fiorio, M. Emadi Andani, A. Marotta, J. Classen and M. Tinazzi. (2014). Placebo-induced changes in excitatory and inhibitory corticospinal circuits during motor performance. 10.1523/JNEUROSCI.3931-13.2014
Cindy S.-Y. Lin MEngSc, Ilona Mogyoros MD, PhD, David Burke MD, DSc. (2000). Recovery of excitability of cutaneous afferents in the median and sural nerves following activity. 10.1002/(SICI)1097-4598(200005)23:5<763::AID-MUS14>3.0.CO;2-D
P. Diederichsen, J. Nørregaard, M. Krogsgaard, T. Fischer-Rasmussen and P. Dyhre-Poulsen. (2004). Reflexes in the shoulder muscles elicited from the human coracoacromial ligament. 10.1016/j.orthres.2003.12.019
T. Krause, R. Kurth, J. Ruben, J. Schwiemann, K. Villringer, M. Deuchert, M. Moosmann, S. Brandt, K. J. Wolf, G. Curio and A. Villringer. (2001). Representational overlap of adjacent fingers in multiple areas of human primary somatosensory cortex depends on electrical stimulus intensity: An fMRI study. 10.1016/S0006-8993(01)02147-3
G. Novembre, V. M. Pawar, R. J. Bufacchi, M. Kilintari, M. Srinivasan, J. C. Rothwell, P. Haggard and G. D. Iannetti. (2018). Saliency detection as a reactive process: Unexpected sensory events evoke corticomuscular coupling. 10.1523/JNEUROSCI.2474-17.2017
Popovich, C. Dockstader, D. Cheyne and R. Tannock. (2010). Sex differences in sensorimotor mu rhythms during selective attentional processing. 10.1016/j.neuropsychologia.2010.10.016
Deguchi, H. Takeuchi, T. Touge, M. Kamoda, M. Tsukaguchi, I. Sasaki and M. Nishioka. (1996). Significance of cognitive function in the elicitation of sympathetic skin response in healthy humans. 10.1016S0165-1838(96)00067-7
Y. Zhang, C. B. Glielmi, Y. Jiang, J. Wang, X. Wang, J. Fang, C. Cui, J. Han, X. Hu and J. Zhang. (2012). Simultaneous CBF and BOLD mapping of high frequency acupuncture induced brain activity. 10.1016/j.neulet.2012.09.050
S. La Cesa, G. Di Stefano, C. Leone, A. Pepe, E. Galosi, F. Alu, A. Fasolino, G. Cruccu, M. Valeriani and A. Truini. (2018). Skin denervation does not alter cortical potentials to surface concentric electrode stimulation: A comparison with laser evoked potentials and contact heat evoked potentials. 10.1002/ejp.1112
S. Hellwig, M. Weisbrod, V. Jochum, M. Rentrop, J. Unger, S. Walther, K. Haefner, A. Roth, P. Fiedler and S. Bender. (2008). Slow cortical potentials in human aversive trace conditioning. 10.1016/j.ijpsycho.2008.02.011
W. Raabe and D. Walk. (2021). Slowly conducting potentials in human sensory nerves. 10.1016j.jneumeth.2020.109045
M. X. Huang, R. R. Lee, K. M. Gaa, T. Song, D. L. Harrington, C. Loh, R. J. Theilmann, J. C. Edgar, G. A. Miller, J. M. Canive and E. Granholm. (2010). Somatosensory system deficits in schizophrenia revealed by MEG during a median-nerve oddball task. 10.1007/s10548-009-0122-5
T. Weiss, K. Walter, D. Spohn, M. Richter, F. Torma and W. H. R. Miltner. (2007). Spatial discrimination learning of electrocutaneous stimuli. 10.1016/j.neulet.2007.09.021
R. P. Dhond, T. Witzel, M. Hämäläinen, N. Kettner and V. Napadow. (2008). Spatiotemporal mapping the neural correlates of acupuncture with MEG. 10.1089/acm.2007.0824
M. Gorka, L. Lieberman, S. A. Shankman and K. L. Phan . (2017). Startle potentiation to uncertain threat as a psychophysiological indicator of fear-based psychopathology: An examination across multiple internalizing disorders . 10.1037/abn0000233
J. M. Castellote and M. Kofler. (2018). StartReact effects in first dorsal interosseous muscle are absent in a pinch task, but present when combined with elbow flexion. 10.1371/journal.pone.0201301
P. Rossi, A. Truini, M. Serrao, G. D. Iannetti, L. Parisi, G. Pozzessere and G. Cruccu. (2002). Sympathetic skin response evoked by laser skin stimulation. https://www.scopus.com/inwardrecord.uri?eid=2-s2.0-0036665129&partnerID=40&md5=a1d26ff59b5cd3c84a0633fe54265c75
I. Weissman-Fogel, A. Dror and R. Defrin. (2015). Temporal and spatial aspects of experimental tonic pain: Understanding pain adaptation and intensification. 10.1002/ejp.562
J. Schliessbach, E. van der Klift, L. Arendt-Nielsen, M. Curatolo and K. Streitberger. (2011). The effect of brief electrical and manual acupuncture stimulation on mechanical experimental pain. 10.1111j.1526-4637.2010.01051.x
F. Musial, K. E. Choi, T. Gabriel, R. Lüdtke, T. Rampp, A. Michalsen and G. Dobos. (2012). The effect of electroacupuncture and tramadol on experimental tourniquet pain. 10.1136/acupmed-2011-010094
A. Ekblom, B. Fjellner and P. Hansson. (1984). The influence of mechanical vibratory stimulation and transcutaneous electrical nerve stimulation on experimental pruritus induced by histamine. 10.1111j.1748-1716.1984.tb07520.x
A. Ekblom and P. Hansson. (1987). Thermal sensitivity is not changed by acute pain or afferent stimulation. 10.1136/jnnp.50.9.1216
M. Tinazzi, M. Fiorio, L. Bertolasi and S. M. Aglioti. (2004). Timing of tactile and visuo-tactile events is impaired in patients with cervical dystonia. 10.1007/s00415-004-0282-x
İ. Çıtak Karakaya, M. G. Karakaya, E. Erğun, S. Elmalı and T. Fırat. (2014). Effects of different frequencies of conventional transcutaneous electrical nerve stimulation on pressure pain threshold and tolerance. 10.3233bmr-130436
D. P. Webster, L. Pellegrini and K. Duffy. (1992). Use of transcutaneous electrical nerve stimulation for fingertip analgesia: a pilot study. 10.1016/s0196-0644(05)80064-2
De Martino, E., Petrini, L., Schabrun, S., & Graven-Nielsen, T. (2018). Cortical Somatosensory Excitability Is Modulated in Response to Several Days of Muscle Soreness. 10.1016/j.jpain.2018.05.004
R. Kakigi and H. Shibasaki. (1991). Effects of age, gender, and stimulus side on scalp topography of somatosensory evoked potentials following median nerve stimulation. 10.1097/00004691-199107010-00008
T. Palmer, D. J. Martin, W. M. Steedman and J. Ravey. (2004). Effects of electric stimulation on C and A delta fiber-mediated thermal perception thresholds. 10.1016/s0003-9993(03)00432-5
T. W. Tourville, D. A. Connolly and B. V. Reed. (2006). Effects of sensory-level high-volt pulsed electrical current ondelayed-onset muscle soreness. 10.1080/02640410500357226
C. D. Mørch, K. Hennings and O. K. Andersen. (2011). Estimating nerve excitation thresholds to cutaneous electrical stimulation by finite element modeling combined with a stochastic branching nerve fiber model. 10.1007s11517-010-0725-8
A. A. Zarei, W. Jensen, A. Faghani Jadidi, E. R. Lontis and S. F. Atashzar. (2022). Gamma-band enhancement of functional brain connectivity following transcutaneous electrical nerve stimulation. 10.1088/1741-2552/ac59a1
T. G. Gasser, E. I. Sandalcioglu, H. Wiedemayer, V. Hans, E. Gizewski, M. Forsting and D. Stolke. (2004). A novel passive functional MRI paradigm for preoperative identification of the somatosensory cortex. 10.1007s10143-003-0318-1
R. Lavine, M. S. Buchsbaum and M. Poncy. (1976). Auditory Analgesia: Somatosensory Evoked Response and Subjective Pain Rating. 10.1111/j.1469-8986.1976.tb00089.x
M. Liang, M. C. Lee, J. O'Neill, A. H. Dickenson and G. D. Iannetti. (2016). Brain potentials evoked by intraepidermal electrical stimuli reflect the central sensitization of nociceptive pathways. 10.1152/jn.00013.2016
Zhao, Rui; Su, Qian; Song, Yingchao; Yang, Qingqing; Wang, Sijia; Zhang, Juan; Qin, Wen; Yu, Chunshui; Liang, Meng. (2022). Brain-activation-based individual identification reveals individually unique activation patterns elicited by pain and touch. 10.1016/j.neuroimage.2022.119436
B. C. McDowell, K. McCormack, D. M. Walsh, D. G. Baxter and J. M. Allen. (1999). Comparative analgesic effects of H-wave therapy and transcutaneous electrical nerve stimulation on pain threshold in humans. 10.1016s0003-9993(99)90051-5
J. L. Chen, T. Thompson, J. Kropotov and J. H. Gruzelier. (2011). Beneficial Effects of Electrostimulation Contingencies on Sustained Attention and Electrocortical Activity. 10.1111/j.1755-5949.2010.00190.x
D. Ristić, P. Spangenberg and J. Ellrich. (2008). Analgesic and antinociceptive effects of peripheral nerve neurostimulation in an advanced human experimental model. 10.1016/j.ejpain.2007.07.013
M. R. Mulvey, H. J. Fawkner and M. I. Johnson. (2015). An Investigation of the Effects of Different Pulse Patterns of Transcutaneous Electrical Nerve Stimulation (TENS) on Perceptual Embodiment of a Rubber Hand in Healthy Human Participants with Intact Limbs. 10.1111/ner.12329
A. O. Hamm, M. K. Greenwald, M. M. Bradley and P. J. Lang . (1993). Emotional learning, hedonic change, and the startle probe . 10.1037/0021-843X.102.3.453
L. S. Claydon, L. S. Chesterton, P. Barlas and J. Sim. (2013). Alternating-frequency TENS effects on experimental pain in healthy human participants: a randomized placebo-controlled trial. 10.1097/AJP.0b013e318262330f
C. C. Chen and M. I. Johnson. (2010). An investigation into the hypoalgesic effects of high- and low-frequency transcutaneous electrical nerve stimulation (TENS) on experimentally-induced blunt pressure pain in healthy human participants. 10.1016/j.jpain.2009.05.008
E. N. van den Broeke, P. de Hemptinne, M. Mercken, D. M. Torta, J. Lambert and A. Mouraux. (2020). Central sensitization of nociceptive pathways demonstrated by robot-controlled pinprick-evoked brain potentials. 10.1016j.clinph.2020.06.020
O. J. Arthurs, T. Donovan, D. J. Spiegelhalter, J. D. Pickard and S. J. Boniface. (2007). Intracortically distributed neurovascular coupling relationships within and between human somatosensory cortices. 10.1093/cercorbhk014
X. P. Zhong, Y. X. Chen, Z. Y. Li, Z. W. Shen, K. M. Kong and R. H. Wu. (2017). Cervical spinal functional magnetic resonance imaging of the spinal cord injured patient during electrical stimulation. 10.1007/s00586-016-4646-6
Enrico De Martino, David A.Seminowicz, Siobhan M.Schabrun, Laura Petrini, Thomas Graven-Nielsen. (2019). High frequency repetitive transcranial magnetic stimulation to the left dorsolateral prefrontal cortex modulates sensorimotor cortex function in the transition to sustained muscle pain. 10.1016/j.neuroimage.2018.10.076
H. H. Andersen, A. I. M. van Laarhoven, F. D. Justesen, J. B. Pedersen, L. L. Sørensen, L. P. Jensen and L. Arendt-Nielsen. (2018). Capsaicin-sensitive cutaneous primary afferents convey electrically induced itch in humans. 10.1016/j.neulet.2017.11.061
L. D. Hamilton, D. Mani, A. M. Almuklass, L. A. Davis, T. Vieira, A. Botter and R. M. Enoka. (2018). Electrical nerve stimulation modulates motor unit activity in contralateral biceps brachii during steady isometric contractions. 10.1152/jn.00235.2018
Y. Jiang, Y. Hao, Y. Zhang, J. Liu, X. Wang, J. Han, J. Fang, J. Zhang and C. Cui. (2012). Thirty minute transcutaneous electric acupoint stimulation modulates resting state brain activities: A perfusion and BOLD fMRI study. 10.1016j.brainres.2012.03.063
J. Witkoś, A. Fusińska-Korpik, M. Hartman-Petrycka and A. Nowak. (2022). An assessment of sensory sensitivity in women suffering from depression using transcutaneous electrical nerve stimulation. 10.7717/peerj.13373
Kofler, Markus; Kumru, Hatice; Schaller, Judith; Saltuari, Leopold. (2013). Blink reflex prepulse inhibition and excitability recovery: Influence of age and sex. 10.1016/j.clinph.2012.07.001
K. Hallén, T. Hrafnkelsdóttir, S. Jern, B. Biber, C. Mannheimer and S. D. Roy. (2010). Transcutaneous electrical nerve stimulation induces vasodilation in healthy controls but not in refractory angina patients. 10.1016j.jpainsymman.2009.12.009
Chih-Chung Chen, Mark I Johnson. (2009). An investigation into the effects of frequency-modulated transcutaneous electrical nerve stimulation (TENS) on experimentally-induced pressure pain in healthy human participants. 10.1016/j.jpain.2009.03.008
S. Cowan, J. McKenna, E. McCrum-Gardner, M. I. Johnson, K. A. Sluka and D. M. Walsh. (2009). An Investigation of the Hypoalgesic Effects of TENS Delivered by a Glove Electrode. 10.1016/j.jpain.2008.12.004
A. R. Ward and W. G. Oliver. (2007). Comparison of the hypoalgesic efficacy of low-frequency and burst-modulated kilohertz frequency currents. 10.2522/ptj.20060203
R. E. Liebano, C. G. Vance, B. A. Rakel, J. E. Lee, N. A. Cooper, S. Marchand, D. M. Walsh and K. A. Sluka. (2013). Transcutaneous electrical nerve stimulation and conditioned pain modulation influence the perception of pain in humans. 10.1002/j.1532-2149.2013.00328.x
Deirdre M. Walsh Dphil a, Andrea S. Lowe Dphil a, Kenneth McCormack BSC a, Jean-Claude Willer MD b, G.David Baxter Dphil a, Jim M. Allen PhD. (1998). Transcutaneous electrical nerve stimulation: Effect on peripheral nerve conduction, mechanical pain threshold, and tactile threshold in humans. 10.1016/s0003-9993(98)90170-8
J. Avendaño-Coy, E. Bravo-Esteban, A. Ferri-Morales, R. Martínez-de la Cruz and J. Gómez-Soriano. (2019). Does Frequency Modulation of Transcutaneous Electrical Nerve Stimulation Affect Habituation and Mechanical Hypoalgesia? A Randomized, Double-Blind, Sham-Controlled Crossover Trial. 10.1093/ptj/pzz054
M. Ebrahimian, M. Razeghi, A. Zamani, Z. Bagheri, K. Rastegar and A. Motealleh. (2018). Does high frequency transcutaneous electrical nerve stimulation (TENS) affect EEG gamma band activity?. 10.22086/jbpe.v0i0.780
J. Avendaño-Coy, J. Gómez-Soriano, C. Goicoechea-García, J. A. Basco-López and J. Taylor. (2017). Effect of Unmodulated 5-kHz Alternating Currents Versus Transcutaneous Electrical Nerve Stimulation on Mechanical and Thermal Pain, Tactile Threshold, and Peripheral Nerve Conduction: A Double-Blind, Placebo-Controlled Crossover Trial. 10.1016/j.apmr.2016.11.020
N. Wang and C. Hui-Chan. (2003). Effects of acupoints TENS on heat pain threshold in normal subjects. https://pubmed.ncbi.nlm.nih.gov/14687475/
H. Ashton, I. Ebenezer, J. F. Golding and J. W. Thompson. (1984). Effects of acupuncture and transcutaneous electrical nerve stimulation on cold-induced pain in normal subjects. 10.1016/0022-3999(84)90052-7
M. De Tommaso, P. Fiore, A. Camporeale, M. Guido, G. Libro, L. Losito, M. Megna, F. Puca and G.Megna. (2003). High and low frequency transcutaneous electrical nerve stimulation inhibits nociceptive responses induced by CO2 laser stimulation in humans. 10.1016/S0304-3940(03)00219-2
J. D. Telles, M. A. G. Schiavon, A. C. S. Costa, P. Rampazo É and R. E. Liebano. (2021). Hypoalgesic Effects of Transcutaneous Electrical Nerve Stimulation Combined With Joint Manipulation: A Randomized Clinical Trial. 10.1016/j.jmpt.2020.09.004
M. A. Menezes, T. A. B. Pereira, L. M. Tavares, B. T. Q. Leite, A. G. R. Neto, L. M. S. Chaves, L. V. Lima, M. E. DaSilva-Grigolleto and J. M. DeSantana. (2018). Immediate effects of transcutaneous electrical nerve stimulation (TENS) administered during resistance exercise on pain intensity and physical performance of healthy subjects: a randomized clinical trial. 10.1007/s00421-018-3919-7
P. M. Minder, J. G. Noble, J. Alves-Guerreiro, I. D. Hill, A. S. Lowe, D. M. Walsh and G. D.Baxter. (2002). Interferential therapy: lack of effect upon experimentally induced delayed onset muscle soreness. 10.1046/j.1475-097x.2002.00441.x
Aarskog, M. I. Johnson, J. H. Demmink, A. Lofthus, V. Iversen, R. Lopes-Martins, J. Joensen and J. M.Bjordal. (2007). Is mechanical pain threshold after transcutaneous electrical nerve stimulation (TENS) increased locally and unilaterally? A randomized placebo-controlled trial in healthy subjects. 10.1002/pri.384
Yue Zhang a, Yin Jiang b, Christopher B. Glielmi c, Longchuan Li c, Xiaoping Hu c, Xiaoying Wang d, Jisheng Han b, Jue Zhang a 1, Cailian Cui b 1, Jing Fang . (2013). Long-duration transcutaneous electric acupoint stimulation alters small-world brain functional networks. 10.1016/j.mri.2013.01.006
N. E. Foster, F. Baxter, D. M. Walsh, G. D. Baxter and J. M. Allen. (1996). Manipulation of transcutaneous electrical nerve stimulation variables has no effect on two models of experimental pain in humans. 10.109700002508-199612000-00009
P. Rampazo da Silva É, V. R. Silva, A. S. Bernardes, F. Matuzawa and R. E. Liebano. (2021). Segmental and extrasegmental hypoalgesic effects of low-frequency pulsed current and modulated kilohertz-frequency currents in healthy subjects: randomized clinical trial. 10.1080/09593985.2019.1650857
Mark I Johnson PhD BSc 1, Helen Wilson BSc. (1997). The Analgesic Effects of Different Swing Patterns of Interferential Currents on Cold-induced Pain. 10.1016/S0031-9406(05)65631-3
A. F. L. Cramp, C. Gilsenan, A. S. Lowe and D. M. Walsh. (2000). The effect of high- and low-frequency transcutaneous electrical nerve stimulation upon cutaneous blood flow and skin temperature in healthy subjects. 10.1046/j.1365-2281.2000.00240.x
J. Alves-Guerreiro, J. G. Noble, A. S. Lowe and D. M. Walsh. (2001). The effect of three electrotherapeutic modalities upon peripheral nerve conduction and mechanical pain threshold. 10.1046/j.1365-2281.2001.00374.x
Dean, D. Bowsher and M. I. Johnson. (2006). The effects of unilateral transcutaneous electrical nerve stimulation of the median nerve on bilateral somatosensory thresholds. 10.1111/j.1475-097X.2006.00689.x
Y. Kim, J. Kim, J. K. Shim, D. W. Suh and B. Yoon. (2014). The hypoalgesic effect of remote tactile sensory modulation on the mechanical sensitivity of trigger points: A randomized controlled study. 10.3233/NRE-141156
R. C. Venancio, S. Pelegrini, D. Q. Gomes, E. Y. Nakano and R. E. Liebano. (2013). Effects of carrier frequency of interferential current on pressure pain threshold and sensory comfort in humans. 10.1016/j.apmr.2012.08.204
G. L. Cheing and W. W. Chan. (2009). Influence of choice of electrical stimulation site on peripheral neurophysiological and hypoalgesic effects. 10.2340/16501977-0350
Johnson, M. I., & Tabasam, G. . (2003). An investigation into the analgesic effects of different frequencies of the amplitude-modulated wave of interferential current therapy on cold-induced pain in normal subjects. 10.1016/S0003-9993(03)00151-5
M. I. Johnson and G. Tabasam. (2003). An investigation into the analgesic effects of interferential currents and transcutaneous electrical nerve stimulation on experimentally induced ischemic pain in otherwise pain-free volunteers. 10.1093/ptj/83.3.208
L. Brown, G. Tabasam, J. M. Bjordal and M. I. Johnson. (2007). An investigation into the effect of electrode placement of transcutaneous electrical nerve stimulation (TENS) on experimentally induced ischemic pain in healthy human participants. 10.1097/AJP.0b013e31814b86a9
M. I. Johnson, C. H. Ashton, D. R. Bousfield and J. W. Thompson. (1991). Analgesic effects of different pulse patterns of transcutaneous electrical nerve stimulation on cold-induced pain in normal subjects. 10.10160022-3999(91)90086-4
H. Ashton, J. F. Golding, V. R. Marsh and J. W. Thompson. (1984). Effects of transcutaneous electrical nerve stimulation and aspirin on late somatosensory evoked potentials in normal subjects. 10.10160304-3959(84)90050-2
M. Buonocore and N. Camuzzini. (2007). Increase of the heat pain threshold during and after high-frequency transcutaneous peripheral nerve stimulation in a group of normal subjects. https://pubmed.ncbi.nlm.nih.gov/17021587/
A. M. Almuklass, R. A. Capobianco, D. F. Feeney, E. Alvarez and R. M. Enoka. (2020). Sensory nerve stimulation causes an immediate improvement in motor function of persons with multiple sclerosis: A pilot study. 10.1016j.msard.2019.101508
R. E. Liebano, B. Rakel, C. G. T. Vance, D. M. Walsh and K. A. Sluka. (2011). An investigation of the development of analgesic tolerance to TENS in humans. 10.1016/j.pain.2010.10.040
M. D. Dounavi, L. S. Chesterton and J. Sim. (2012). Effects of interferential therapy parameter combinations upon experimentally induced pain in pain-free participants: a randomized controlled trial. 10.2522/ptj.20110104
C. Grillon, J. P. Baas, S. Lissek, K. Smith and J. Milstein . (2004). Anxious Responses to Predictable and Unpredictable Aversive Events . 10.1037/0735-7044.118.5.916
F. Kalbe and L. Schwabe . (2020). Beyond arousal: Prediction error related to aversive events promotes episodic memory formation . 10.1037/xlm0000728
A. H. K. Wong and P. F. Lovibond . (2021). Breakfast or bakery? The role of categorical ambiguity in overgeneralization of learned fear in trait anxiety . 10.1037/emo0000739;10.1037/emo0000739.supp(Supplemental)
G. Coppola, D. Di Lenola, C. Abagnale, F. Ferrandes, G. Sebastianelli, F. Casillo, C. Di Lorenzo, M. Serrao, M.Evangelista, J. Schoenen and F. Pierelli. (2020). Short-latency afferent inhibition and somato-sensory evoked potentials during the migraine cycle: Surrogate markers of a cycling cholinergic thalamo-cortical drive?. 10.1186s10194-020-01104-7
G. Coppola, F. Cortese, M. Bracaglia, C. Di Lorenzo, M. Serrao, D. Magis and F. Pierelli. (2020). The function of the lateral inhibitory mechanisms in the somatosensory cortex is normal in patients with chronic migraine. 10.1016j.clinph.2020.01.009
D. Sato, K. Yamashiro, H. Onishi, Y. Shimoyama, T. Yoshida and A. Maruyama. (2012). The effect of water immersion on short-latency somatosensory evoked potentials in human. 10.1186/1471-2202-13-13
S. Springer, Z. Kozol and Z. Reznic. (2018). Ulnar Nerve Conduction Block Using Surface Kilohertz Frequency Alternating Current: A Feasibility Study. 10.1111/aor.13119
J. Fiess, B. Rockstroh, R. Schmidt and A. Steffen. (2015). Emotion regulation and functional neurological symptoms: Does emotion processing convert into sensorimotor activity?. 10.1016/j.jpsychores.2015.10.009
C. S. Yuan, A. S. Attele, L. Dey, J. P. Lynch and X. Guan. (2002). Transcutaneous electrical acupoint stimulation potentiates analgesic effect of morphine. 10.1177/009127002401102812
H. Mochizuki, K. Inui, H. C. Tanabe, L. F. Akiyama, N. Otsuru, K. Yamashiro, A. Sasaki, H. Nakata, N. Sadato and R. Kakigi. (2009). Time course of activity in itch-related brain regions: A combined MEG-fMRI study. 10.1152jn.00460.2009
M. P. Veldman, N. M. Maurits, I. Zijdewind, N. A. Maffiuletti, S. van Middelkoop, J. C. Mizelle and T.Hortobágyi. (2018). Somatosensory electrical stimulation improves skill acquisition, consolidation, and transfer by increasing sensorimotor activity and connectivity. 10.1152/jn.00860.2017
C. C. Chen, M. I. Johnson, S. McDonough and F. Cramp. (2007). The effect of transcutaneous electrical nerve stimulation on local and distal cutaneous blood flow following a prolonged heat stimulus in healthy subjects. 10.1111/j.1475-097X.2007.00731.x
M. C. Stoeckel, B. Pollok, A. Schnitzler and R. J. Seitz. (2007). Studying the human somatosensory hand area: A new way to compare fMRI and MEG. 10.1016/j.jneumeth.2007.05.016
P. Svensson, T. S. Miles, D. McKay and M. C. Ridding. (2003). Suppression of motor evoked potentials in a hand muscle following prolonged painful stimulation. 10.1016/S1090-3801(02)00050-2
S. Kangas, E. Vuoriainen, X. Li, P. Lyyra and P. Astikainen . (2022). Somatosensory deviance detection ERPs and their relationship to analogous auditory ERPs and interoceptive accuracy . 10.1027/0269-8803/a000288;10.1027/0269-8803/a000288(Supplemental)
S. J. Summers, S. M. Schabrun, W. Marinovic and L. S. Chipchase. (2017). Peripheral electrical stimulation increases corticomotor excitability and enhances the rate of visuomotor adaptation. 10.1016/j.bbr.2017.01.016
D. Lelic, M. Valeriani, I. W. Fischer, A. Dahan and A. M. Drewes. (2017). Venlafaxine and oxycodone have different effects on spinal and supraspinal activity in man: a somatosensory evoked potential study. 10.1111/bcp.13177
P. J. Theuvenet, B. W. Van Dijk, M. J. Peters, J. M. Van Ree, F. L. Lopes Da Silva and A. C. N.Chen. (2005). Whole-head MEG analysis of cortical spatial organization from unilateral stimulation of median nerve in both hands: No complete hemispheric homology. 10.1016/j.neuroimage.2005.06.010
F. X. de Brito, C. Luz-Santos, J. R. Camatti, R. J. de Souza da Fonseca, G. Suzarth, L. M. C. Moraes, M. L. da Silva, J. R. T. da Silva, R. Vercelino, K. N. Sá, Y. Zana and A. F. Baptista. (2022). Electroacupuncture modulates cortical excitability in a manner dependent on the parameters used. 10.1177/09645284211057560
S. Abdulwahab and M. Sabbahi. (2000). Neck retractions, cervical root decompression, and radicular pain. 10.2519/jospt.2000.30.1.4
B. C. Clark, L. C. Issac, J. L. Lane, L. A. Damron and R. L. Hoffman. (2008). Neuromuscular plasticity during and following 3 wk of human forearm cast immobilization. 10.1152/japplphysiol.90530.2008
T. Raij, J. Karhu, D. Kičić, P. Lioumis, P. Julkunen, F. H. Lin, J. Ahveninen, R. J. Ilmoniemi, J. P. Mäkelä, M. Hämäläinen, B. R. Rosen and J. W. Belliveau. (2008). Parallel input makes the brain run faster. 10.1016j.neuroimage.2008.01.055
M. Kanda, T. Nagamine, A. Ikeda, S. Ohara, T. Kunieda, N. Fujiwara, S. Yazawa, N. Sawamoto, R. Matsumoto, W. Taki and H. Shibasaki. (2000). Primary somatosensory cortex is actively involved in pain processing in human. 10.1016/S0006-8993(99)02274-X
R. R. Guirro, E. C. Guirro and N. T. de Sousa. (2015). Sensory and motor thresholds of transcutaneous electrical stimulation are influenced by gender and age. 10.1016/j.pmrj.2014.07.004
A. C. N. Chen, P. J. Theuvenet, J. C. De Munck, M. J. Peters, J. M. Van Ree and F. L. L. Da Silva. (2012). Sensory handedness is not reflected in cortical responses after basic nerve stimulation: A MEG study. 10.1007s10548-011-0209-7
H. Tokimura, V. Di Lazzaro, Y. Tokimura, A. Oliviero, P. Profice, A. Insola, P. Mazzone, P. Tonali and J. C. Rothwell. (2000). Short latency inhibition of human hand motor cortex by somatosensory input from the hand. 10.1111/j.1469-7793.2000.t01-1-00503.x
P. Manganotti, E. Formaggio, S. F. Storti, M. Avesani, M. Acler, F. Sala, S. Magon, G. Zoccatelli, F. Pizzini, F. Alessandrini, A. Fiaschi and A. Beltramello. (2009). Steady-state activation in somatosensory cortex after changes in stimulus rate during median nerve stimulation. 10.1016/j.mri.2009.05.009
A. Cervera, M. Veciana and J. Valls-Solé. (2002). Sympathetic sudomotor skin responses induced by laser stimuli in normal human subjects. 10.1016/S0304-3940(02)01117-5
A. L. Wang, A. Mouraux, M. Liang and G. D. Iannetti. (2008). The enhancement of the N1 wave elicited by sensory stimuli presented at very short inter-stimulus intervals is a general feature across sensory system. 10.1371journal.pone.0003929
D. Mani, D. F. Feeney and R. M. Enoka. (2019). The modulation of force steadiness by electrical nerve stimulation applied to the wrist extensors differs for young and older adults. 10.1007/s00421-018-4025-6
M. B. E. Eriksson, I. Rosén and B. Sjölund. (1985). Thermal sensitivity in healthy subjects is decreased by a central mechanism after TNS. 10.1016/0304-3959(85)90023-5
P. Hiedl, A. Struppler and M. Gessler. (1979). Tns-evoked long loop effects. 10.1159/000102359
V. Kunde and R. D. Treede. (1993). Topography of middle-latency somatosensory evoked potentials following painful laser stimuli and non-painful electrical stimuli. 10.1016/0168-5597(93)90052-Q
K. Hennings, K. S. Frahm, L. Petrini, O. K. Andersen, L. Arendt-Nielsen and C. D. Mørch. (2017). Membrane properties in small cutaneous nerve fibers in humans. 10.1002/mus.25234
A. Steyaert, C. Lenoir, P. Lavand'homme, E. N. van den Broeke and A. Mouraux. (2022). Multichannel transcranial direct current stimulation over the left dorsolateral prefrontal cortex may modulate the induction of secondary hyperalgesia, a double-blinded cross-over study in healthy volunteers. 10.1371/journal.pone.0270047
R. Park, M. S. Wallace and G. Schulteis. (2001). Relative sensitivity to alfentanil and reliability of current perception threshold vs von Frey tactile stimulation and thermal sensory testing. 10.1046/j.1529-8027.2001.01025.x
B. C. H. Tsui, T. J. Shakespeare, D. H. Leung, J. H. Tsui and G. N. Corry. (2013). Reproducibility of current perception threshold with the Neurometer® vs the Stimpod NMS450 peripheral nerve stimulator in healthy volunteers: An observational study. 10.1007/s12630-013-9965-z
M. Rosa Esteve and L. Camacho. (2008). Anxiety sensitivity, body vigilance and fear of pain. 10.1016j.brat.2008.02.012
T. Nielson Hoberg, S. Frahm, K. Hennings, L. Arendt-Nielsen and C. Dahl Mørch. (2019). Assessing the modulation of cutaneous sensory fiber excitability using a fast perception threshold tracking technique. 10.1002/mus.26520
M. Krzanowska, K. Muszer, K. Chabowski and A. Reich. (2015). Assessment of the sensory threshold in patients with atopic dermatitis and psoriasis. 10.5114/pdia.2014.44013
M. Kafri, N. Zaltsberg and R. Dickstein. (2015). EMG activity of finger flexor muscles and grip force following low-dose transcutaneous electrical nerve stimulation in healthy adult subjects. 10.3109/08990220.2014.937413
S. Nakatani-Enomoto, M. Yamazaki, Y. Kamimura, M. Abe, K. Asano, H. Enomoto, K. Wake, S. Watanabe and Y. Ugawa. (2019). Frequency-dependent current perception threshold in healthy Japanese adults. 10.1002bem.22175

K. J. Meador, P. G. Ray, L. Day, H. Ghelani and D. W. Loring. (1998). Physiology of somatosensory perception: Cerebral lateralization and extinction. 10.1212/WNL.51.3.721

U. Yilmaz, M. A. Ciol, R. E. Berger and C. C. Yang. (2010). Sensory perception thresholds in men with chronic pelvic pain syndrome. 10.1016/j.urology.2009.08.013

J. S. Feine, C. E. Chapman, J. P. Lund, G. H. Duncan and M. C. Bushnell. (1990). The perception of painful and nonpainful stimuli during voluntary motor activity in man. 10.3109/08990229009144702

B. Geng, K. Yoshida and W. Jensen. (2011). Impacts of selected stimulation patterns on the perception threshold in electrocutaneous stimulation. 10.1186/1743-0003-8-9

W. Gu, W. Jiang, J. He, S. Liu and Z. Wang. (2015). Blockade of the brachial plexus abolishes activation of specific brain regions by electroacupuncture at LI4: A functional MRI study. 10.1136/acupmed-2015-010901

Y. Liu, P. Xi, B. Li, M. Zhang, H. Liu, R. Tang, S. Xin, Q. Huang, J. He, Z. Liu, Z. Yuan and Y. Lang. (2022). Effect of neuromorphic transcutaneous electrical nerve stimulation (nTENS) of cortical functional networks on tactile perceptions: An event-related electroencephalogram study. 10.1088/1741-2552/ac5bf6

M. Fiorio, M. Emadi Andani, S. Recchia and M. Tinazzi. (2018). The somatosensory temporal discrimination threshold changes after a placebo procedure. 10.1007/s00221-018-5357-5

N. Otsuru, A. Hashizume, D. Nakamura, Y. Endo, K. Inui, R. Kakigi and L. Yuge. (2014). Sensory incongruence leading to hand disownership modulates somatosensory cortical processing. 10.1016/j.cortex.2014.05.005

S. Franco, F. S. Paulitsch, A. P. C. Pereira, A. O. Teixeira, C. N. Martins, A. M. V. Silva, R. D. M. Plentz, M. C.Irigoyen and L. U. Signori. (2014). Effects of different frequencies of transcutaneous electrical nerve stimulation on venous vascular reactivity. 10.1590/1414-431X20143767

J. Bloeckl, S. Raps, M. Weineck, R. Kob, T. Bertsch, W. Kemmler and D. Schoene. (2022). Feasibility and Safety of Whole-Body Electromyostimulation in Frail Older People—A Pilot Trial. 10.3389/fphys.2022.856681

M. Grönroos, H. Naukkarinen and A. Pertovaara. (1994). Capsaicin-induced central facilitation of a sympathetic vasoconstrictor response to painful stimulation in humans. 10.1016/0304-3940(94)90788-9

Z. Hanzlíková, M. Kofler, M. Slovák, G. Věchetová, A. Fečíková, D. Kemlink, T. Sieger, E. Růžička, J. Valls-Solé, M. J. Edwards and T. Serranová. (2019). Prepulse inhibition of the blink reflex is abnormal in functional movement disorders. 10.1002/mds.27706

M. Lotze, A. M. Ladda, S. Roschka, T. Platz and H. R. Dinse. (2017). Priming Hand Motor Training with Repetitive Stimulation of the Fingertips; Performance Gain and Functional Imaging of Training Effects. 10.1016j.brs.2016.10.004

A. B. McCambridge, J. W. Stinear, S. Peek and W. D. Byblow. (2017). Propriospinal cutaneous-induced EMG suppression is unaltered by anodal tDCS of healthy motor cortex. 10.1016/j.clinph.2017.06.035

A. A. Zarei, A. F. Jadidi, E. R. Lontis and W. Jensen. (2021). Short-term suppression of somatosensory evoked potentials and perceived sensations in healthy subjects following tens. 10.1109/TBME.2021.3051307

P. D. Drummond. (2008). Depletion of noradrenaline inhibits electrically-evoked pain in the skin of the human forearm. 10.1016/j.ejpain.2007.05.001

L. S. Chipchase, S. M. Schabrun and P. W. Hodges. (2011). Corticospinal excitability is dependent on the parameters of peripheral electric stimulation: A preliminary study. 10.1016/j.apmr.2011.01.011

T. R. Han, D. Y. Kim, S. J. Lim and K. J. Lee. (2007). The control of parameters within the therapeutic range in neuromuscular electrical stimulation. 10.1080/00207450500534134

C. V. Turco, R. S. Rehsi, M. B. Locke and A. J. Nelson. (2021). Biological sex differences in afferent-mediated inhibition of motor responses evoked by TMS. 10.1016/j.brainres.2021.147657

Lenz, M.; H√∂ffken, O.; Stude, P.; Lissek, S.; Schwenkreis, P.; Reinersmann, A.; Frettl√∂h, J.; Richter, H.; Tegenthoff, M.; Maier, C. (2011). Bilateral somatosensory cortex disinhibition in complex regional pain syndrome type I. 10.1212/WNL.0b013e31822e1436

S. K. Yildiz, N. Yildiz, B. Korkmaz, B. Altunrende, A. R. Gezici and S. Alkoy. (2008). Sympathetic skin responses from frontal region in migraine headache: A pilot study. 10.1111/j.1468-2982.2008.01574.x

A. H. Poulsen, B. Van Den Berg, F. Arguissain, J. Tigerholm, J. R. Buitenweg, O. K. Andersen and C. D. M rch. (2022). Novel surface electrode design for preferential activation of cutaneous nociceptors. https://iopscience.iop.org/article/10.1088/1741-2552/ac4950/pdf?casa_token=4nXSKpPTVrQAAAAA:Du68dNQkEBnoJYrkukI5-f6Rs-bZEjMZNp5p4UWjPhGhK2zzpEcisa47WMjgUkPRGubbW1Gd

G. L. Cheing and C. W. Hui-Chan. (2003). Analgesic effects of transcutaneous electrical nerve stimulation and interferential currents on heat pain in healthy subjects. 10.1080/16501970306101

C. F. Sambo, M. Liang, G. Cruccu and G. D. Iannetti. (2012). Defensive peripersonal space: The blink reflex evoked by hand stimulation is increased when the hand is near the face. 10.1152/jn.00731.2011

N. Ravichandran, M. Y. Teo, K. Aw and A. McDaid. (2020). Design of Transcutaneous Stimulation Electrodes for Wearable Neuroprostheses. 10.1109/TNSRE.2020.2994900

H. Franssen, D. F. Stegeman, J. Moleman and R. P. Schoobaar. (1992). Dipole modelling of median nerve SEPs in normal subjects and patients with small subcortical infarcts. 10.1016/0168-5597(92)90027-9

S. Sugiyama, N. Takeuchi, K. Inui, M. Nishihara and T. Shioiri. (2018). Effect of acceleration of auditory inputs on the primary somatosensory cortex in humans. 10.1038/s41598-018-31319-3

M. Boakye, B. R. Krauss, S. C. Huckins, L. Zhang, N. M. Szeverenyi and C. J. Hodge Jr. (2002). Effects of hyperoxia on human sensorimotor cortex activity produced by electrical stimulation of the median nerve: A functional magnetic resonance imaging study. 10.1016/S0304-3940(01)02406-5

E. N. Van Den Broeke and A. Mouraux. (2014). Enhanced brain responses to C-fiber input in the area of secondary hyperalgesia induced by high-frequency electrical stimulation of the skin. 10.1152/jn.00342.2014

P. Srisa-an, L. Lei and I. M. Tarkka. (1996). Middle latency somatosensory evoked potentials: Noninvasive source analysis. 10.1097/00004691-199603000-00006

R. D. Treede and V. Kunde. (1995). Middle-latency somatosensory evoked potentials after stimulation of the radial and median nerves: Component structure and scalp topography. 10.1097/00004691-199505010-00007

J. P. Bartels, A. I. M. van Laarhoven, M. Stroo, K. Hijne, K. J. Peerdeman, A. R. T. Donders, P. C. M. van de Kerkhofand A. W. M. Evers. (2017). Minimizing nocebo effects by conditioning with verbal suggestion: A randomized clinical trial in healthy humans. 10.1371/journal.pone.0182959

D. J. P. Bartels, A. I. M. Van Laarhoven, P. C. M. Van de Kerkhof and A. W. M. Evers. (2018). Nocebo effects and scratching behaviour on itch. 10.2340/00015555-2979

S. Maris, M. Brands, D. Lenskens, G. Braeken, S. Kemnitz, H. Vanhove, M. Mc Laughlin, R. Meesen, B. Brône and B. Stessel. (2022). Transcutaneous electrical nerve inhibition using medium frequency alternating current. 10.1038s41598-022-18974-3

A. C. Merlocco, K. L. Redington, T. Disenhouse, S. C. Strantzas, R. Gladstone, C. Wei, M. B. Tropak, C. Manlhiot, J. Li and A. N. Redington. (2014). Transcutaneous electrical nerve stimulation as a novel method of remote preconditioning: In vitro validation in an animal model and first human observations. 10.1007s00395-014-0406-0

K. Ishibashi, D. Ishii, S. Yamamoto, A. Noguchi, K. Tanamachi and Y. Kohno. (2021). Opposite modulations of corticospinal excitability by intermittent and continuous peripheral electrical stimulation in healthy subjects. 10.1016/j.neulet.2020.135467

J. H. Blok, A. Ruitenberg, E. M. Maathuis and G. H. Visser. (2007). The electrophysiological muscle scan. 10.1002mus.20838

T. Götz, R. Huonker, O. W. Witte and J. Haueisen. (2014). Thalamocortical impulse propagation and information transfer in EEG and MEG. 10.1097/WNP.0000000000000048

J. F. Riley and F. M. Levine. (1988). Counterstimulation and pain perception: effects of electrocutaneous vs. auditory stimulation upon cold pressor pain. 10.1016/0304-3959(88)90135-2

M. I. Johnson, C. H. Ashton, D. R. Bousfield and J. W. Thompson. (1989). Analgesic effects of different frequencies of transcutaneous electrical nerve stimulation on cold-induced pain in normal subjects. 10.10160304-3959(89)90010-9

Vo, Lechi; Drummond, Peter D. (2013). Analgesia to pressure,Äďpain develops in the ipsilateral forehead after high- and low-frequency electrical stimulation of the forearm. 10.1007/s00221-013-3776-x

A. R. Ward, V. J. Robertson and R. J. Makowski. (2002). Optimal frequencies for electric stimulation using medium-frequency alternating current. 10.1053/apmr.2002.33116

T. Kida, T. Wasaka, H. Nakata, K. Akatsuka and R. Kakigi. (2006). Active attention modulates passive attention-related neural responses to sudden somatosensory input against a silent background. 10.1007s00221-006-0578-4

T. Lundeberg, S. Eriksson, S. Lundeberg and M. Thomas. (1989). Acupuncture and sensory thresholds. 10.1142s0192415x89000176

K. C. Tong, S. K. Lo and G. L. Cheing. (2007). Alternating frequencies of transcutaneous electric nerve stimulation: does it produce greater analgesic effects on mechanical and thermal pain thresholds?. 10.1016j.apmr.2007.07.017

Gatzounis, Rena; Schrooten, Martien G. S.; Crombez, Geert; Vlaeyen, Johan W. S. (2018). Activity interruptions by pain impair activity resumption, but not more than activity interruptions by other stimuli: An experimental investigation. 10.1097/j.pain.0000000000001079

Willer, J. C., Roby, A., Boulu, P., & Boureau, F. (1982). Comparative effects of electroacupuncture and transcutaneous nerve stimulation on the human blink reflex. 10.1016/0304-3959(82)90133-6

D. Boërio, J. Y. Hogrel, A. Créange and J. P. Lefaucheur. (2005). A reappraisal of various methods for measuring motor nerve refractory period in humans. 10.1016/j.clinph.2004.11.018

B. Rakel, N. Cooper, H. J. Adams, B. R. Messer, L. A. Frey Law, D. R. Dannen, C. A. Miller, A. C. Polehna, R. C. Ruggle, C. G. Vance, D. M. Walsh and K. A. Sluka. (2010). A new transient sham TENS device allows for investigator blinding while delivering a true placebo treatment. 10.1016/j.jpain.2009.07.007

Christoph Benke, Manuela G. Alius, Alfons O. Hamm, Christiane A. Pané-Farré,. (2023). Defensive Mobilization During Anticipation of Symptom Provocation: Association With Panic Pathology,. 10.1016/j.bpsc.2021.11.005

Błażejewski G., Witkoś J., Hartman-Petrycka M. (2024). Changes in sensitivity and hedonic rating to transcutaneous electrical nerve stimulation following COVID-19. 10.1038/s41598-024-51596-5

Borbjerg, M. K., Antonsson, E., Røikjer, J., Ejskjaer, N., & Mørch, C. D. (2023). The stability of perception threshold tracking for long session evaluation of Aβ- and Aδ-fiber function. 10.1002/mus.27967

Drusko, A., Baumeister, D., McPhee Christensen, M., Kold, S., Fisher, V. L., Treede, R. D., .. & Tesarz, J. (2023). A novel computational approach to pain perception modelling within a Bayesian framework using quantitative sensory testing. 10.1038/s41598-023-29758-8

Witkoś, J., Hartman-Petrycka, M., & Błażejewski, G. (2023). The Effects of Sex, Women's Body Composition and Monthly Cycle Phases on the Sensory Threshold of Upper Limb to Transcutaneous Electrical Nerve Stimulation in Healthy Subjects. 10.3390/app13148365

No healthy adult participants

S. Numajiri, H. Sakurai, K. Sugibayashi, Y. Morimoto, H. Omiya, H. Takenaka and N. Akiyama. (1993). Comparison of depolarizing and direct current systems on iontophoretic enhancement of transport of sodium benzoate through human and hairless rat skin. 10.1111/j.2042-7158.1993.tb05662.x

W. W. Leung, A. Y. Jones, S. S. Ng, C. Y. Wong and J. F. Lee. (2013). Acupuncture transcutaneous electrical nerve stimulation reduces discomfort associated with barostat-induced rectal distension: a randomized-controlled study. 10.3748/wjg.v19.i3.381

Kathleen Watson Lin 1, Tonita E Wroolie 1, Thalia Robakis 1, Natalie L Rasgon. (2015). Adjuvant pioglitazone for unremitted depression: Clinical correlates of treatment response. 10.1016/j.pscychresns.2015.10.013

N. Saito, R. Shima, C. T. Yen, R. C. Yang, E. Ito and T. Yoshioka. (2019). Adhesive pyramidal thorn patches provide pain relief to athletes. 10.1002/kjm2.12044

Seegers, J. C., Lottering, M. L., Joubert, A. M., Joubert, F., Koorts, A., Engelbrecht, C. A., & van Papendorp, D. H. (2002). A pulsed DC electric field affects P2-purinergic receptor functions by altering the ATP levels in in vitro and in vivo systems. 10.1054/mehy.2001.1506

K.-Y. Lee, Y.-J. Lee and S.-H. Koh. (2009). Usefulness of the median terminal latency ratio in the diagnosis of carpal tunnel syndrome. 10.1016/j.clinph.2008.12.041

J. Lorenz, K. Kunze and B. Bromm. (1998). Differentiation of conversive sensory loss and malingering by P300 in a modified oddball task. 10.1097/00001756-199801260-00003

R. Schmidt, I. P. Kleggetveit, B. Namer, T. Helås, O. Obreja, M. Schmelz and E. Jørum. (2012). Double spikes to single electrical stimulation correlates to spontaneous activity of nociceptors in painful neuropathy patients. 10.1016/j.pain.2011.10.041

H. Suh. (2009). Effect of 3-electrode electrical stimulation on current delivery and healing in chronic wounds. https://pubmed.ncbi.nlm.nih.gov/19821762/

Y. J. Cho, D. E. Jung, K. Nam, J. Bae, S. Lee and Y. Jeon. (2022). Effects of transcutaneous electrical nerve stimulation on myocardial protection in patients undergoing aortic valve replacement: a randomized clinical trial. 10.1186s12871-022-01611-x

Schliessbach J, Siegenthaler A, Bütikofer L, Vuilleumier P, Jüni P, Stamer U, Arendt-Nielsen L, Curatolo M. (2018). Predicting drug efficacy in chronic low back pain by quantitative sensory tests. 10.1002/ejp.1183

S. Miyoshi, H. LÜDers, M. Kato and Y. Kuroiwa. (1971). The Somatosensory Evoked Potential in Patients with Cerebrovascular Diseases. 10.1111/j.1440-1819.1971.tb01484.x

P. E. Crago. (2019). Neuromodulation by combined sensory and motor stimulation in the peripheral nerve: Tendon organ afferent activity. 10.1088/1741-2552/aaeaa9

A. I. M. van Laarhoven, F. W. Kraaimaat, O. H. Wilder-Smith, P. C. M. van de Kerkhof, H. Cats, P. L. C. M. van Riel and A. W. M. Evers. (2007). Generalized and symptom-specific sensitization of chronic itch and pain. 10.1111j.1468-3083.2007.02215.x

C. N. Sang, M. P. Hostetter, R. H. Gracely, A. S. Chappell, D. D. Schoepp, G. Lee, S. Whitcup, R. Caruso and M. B. Max. (1998). AMPA/kainate antagonist LY293558 reduces capsaicin-evoked hyperalgesia but not pain in normal skin in humans. 10.1097/00000542-199811000-00005

C. Skarke, J. Darimont, H. Schmidt, G. Geisslinger and J. Lötsch. (2003). Analgesic effects of morphine and morphine-6-glucuronide in a transcutaneous electrical pain model in healthy volunteers. 10.1067/mcp.2003.5

E. Olofsen, R. Romberg, H. Bijl, R. Mooren, F. Engbers, B. Kest and A. Dahan. (2005). Alfentanil and placebo analgesia: no sex differences detected in models of experimental pain. 10.1097/00000542-200507000-00020

A. B. Gorelick, S. S. Koshy, F. G. Hooper, T. C. Bennett, W. D. Chey and W. L. Hasler. (1998). Differential effects of amitriptyline on perception of somatic and visceral stimulation in healthy humans. 10.1152ajpgi.1998.275.3.G460

Richard J. Haier 1, Chandra Reynolds 1, Elizabeth Prager 1, Steven Cox 2, Monte S. Buchsbaum 1. (1991). Flurbiprofen, caffeine and analgesia: Interaction with introversion/extraversion. 10.1016/0191-8869(91)90211-S

J. E. Stambaugh, Jr., I. W. Wainer and D. M. Hemphill. (1976). Therapeutic equivalency of two lidocaine preparations. https://pubmed.ncbi.nlm.nih.gov/998645/

Voller, T. Sycha, B. Gustorff, L. Schmetterer, S. Lehr, H. G. Eichler, E. Auff and P. Schnider. (2003). A randomized, double-blind, placebo controlled study on analgesic effects of botulinum toxin A. 10.121201.wnl.0000086374.92906.6a

M. G. Craske, K. B. Wolitzky-Taylor, S. Mineka, R. Zinbarg, A. M. Waters, S. Vrshek-Schallhorn, A. Epstein, B. Naliboff and E. Ornitz . (2012). Elevated responding to safe conditions as a specific risk factor for anxiety versus depressive disorders: Evidence from a longitudinal investigation . 10.1037/a0025738

A. Chien, E. Eliav and M. Sterling. (2008). Hypoesthesia occurs in acute whiplash irrespective of pain and disability levels and the presence of sensory hypersensitivity. 10.1097/AJP.0b013e3181773b95

Rejected after narrowing inclusion criteria

M. A. Tucker, M. F. Andrew, S. J. Ogle and J. G. Davison. (1989). Age-associated change in pain threshold measured by transcutaneous neuronal electrical stimulation. 10.1093/ageing/18.4.241

I. Nyklíček and A. Vingerhoets. (2000). Alexithymia is associated with low tolerance to experimental painful stimulation. 10.1016/s0304-3959(99)00295-x

Hughes, Nicola; Bennett, Michael I.; Johnson, Mark I.. (2013). An investigation into the magnitude of the current window and perception of transcutaneous electrical nerve stimulation (TENS) sensation at various frequencies and body sites in healthy human participants. 10.1097/AJP.0b013e3182579919

D. B. Pfau, T. Klein, D. Putzer, E. M. Pogatzki-Zahn, R. D. Treede and W. Magerl. (2011). Analysis of hyperalgesia time courses in humans after painful electrical high-frequency stimulation identifies a possible transition from early to late LTP-like pain plasticity. 10.1016/j.pain.2011.02.037

J. L. K. Kramer, A. J. Moss, P. Taylor and A. Curt. (2008). Assessment of posterior spinal cord function with electrical perception threshold in spinal cord injury. 10.1089/neu.2007.0503

Claes, Nathalie; Crombez, Geert; Meulders, Ann; Vlaeyen, Johan W. S.. (2016). Between the devil and the deep blue sea: Avoidance-avoidance competition increases pain-related fear and slows decision-making. 10.1016/j.jpain.2015.12.005

R. Issa, M. Julien, E. Décary, O. Verdonck, L. P. Fortier, P. Drolet and P. Richebé. (2017). Evaluation of the analgesia nociception index (ANI) in healthy awake volunteers. 10.1007/s12630-017-0887-z

Chen, J. Zhang, P. Wang, J. Guo, M. Zhou and L. He. (2015). Functional Alterations of Pain Processing Pathway in Migraine Patients with Cutaneous Allodynia. 10.1111/pme.12690

E. N. van den Broeke, C. Lenoir and A. Mouraux. (2016). Secondary hyperalgesia is mediated by heat-insensitive A-fibre nociceptors. 10.1113/JP272599

M. W. Hartmannsgruber, C. P. Swamidoss, A. Budde, S. Qadir, S. J. Brull and D. G. Silverman. (1999). A method for overcoming the ceiling effect of bounded pain scales. 10.1023/a:1009961222017

M. Grönroos and A. Pertovaara. (1994). A selective suppression of human pain sensitivity by carbon dioxide: central mechanisms implicated. 10.1007/bf00599245

R. Kenntner-Mabiala and P. Pauli. (2005). Affective modulation of brain potentials to painful and nonpainful stimuli. 10.1111/j.1469-8986.2005.00310.x

J. Zaman, W. Vanpaemel, C. Aelbrecht, F. Tuerlinckx and J. W. S. Vlaeyen. (2017). Biased pain reports through vicarious information: A computational approach to investigate the role of uncertainty. 10.1016j.cognition.2017.07.009

W. Blersch, W. J. Schulte-Mattler, S. Przywara, A. May, H. Bigalke and K. Wohlfarth. (2002). Botulinum toxin A and the cutaneous nociception in humans: A prospective, double-blind, placebo-controlled, randomized study. 10.1016S0022-510X(02)00313-1

F. T. Nickel, S. Ott, S. Möhringer, M. Saake, A. Dörfler, F. Seifert and C. Maihöfner. (2014). Brain correlates of short-term habituation to repetitive electrical noxious stimulation. 10.1002/j.1532-2149.2013.00339.x

Naglatzki, R. P.; Schlamann, M.; Gasser, T.; Ladd, M. E.; Sure, U.; Forsting, M.; Gizewski, E. R.. (2012). Cerebral somatic pain modulation during autogenic training in fMRI. 10.1002/j.1532-2149.2012.00138.x

S. Angst, B. Ramaswamy, M. F. Davies and M. Maze. (2004). Comparative analgesic and mental effects of increasing plasma concentrations of dexmedetomidine and alfentanil in humans. 10.109700000542-200409000-00024

C. Maihöfner, F. Jesberger, F. Seifert and M. Kaltenhäuser. (2010). Cortical processing of mechanical hyperalgesia: A meg study. 10.1016/j.ejpain.2009.02.007

C. Lucantoni, S. Marinelli, A. Refe and F. Tomassini. (1997). Course of pain sensitivity in aging: Pathogenetical aspects of silent cardiopathy. 10.1016/S0167-4943(96)00765-0

P. Pauli, P. Montoya and G.-E. Martz . (1996). Covariation bias in panic-prone individuals . 10.10370021-843X.105.4.658

Y. Maruyama, A. Kawano, S. Okamoto, T. Ando, Y. Ishitobi, Y. Tanaka, A. Inoue, J. Imanaga, M. Kanehisa, H. Higuma, T. Ninomiya, J. Tsuru, H. Hanada and J. Akiyoshi. (2012). Differences in salivary alpha-amylase and cortisol responsiveness following exposure to electrical stimulation versus the trier social stress tests. 10.1371journal.pone.0039375

Vaseghi, M. Zoghi and S. Jaberzadeh. (2015). Differential effects of cathodal transcranial direct current stimulation of prefrontal, motor and somatosensory cortices on cortical excitability and pain perception - a double-blind randomised sham-controlled study. 10.1111/ejn.13043

A. Ter Minassian, E. Ricalens, S. Humbert, F. Duc, C. Aubé and L. Beydon. (2013). Dissociating anticipation from perception: Acute pain activates default mode network. 10.1002/hbm.22062

J. H. G. Blom, C. H. Wiering and R. H. J. Van der Lubbe . (2012). Distraction reduces both early and late electrocutaneous stimulus evoked potentials . 10.1027/0269-8803/a000079

A. I. M. Van Laarhoven, S. Van Damme, A. P. M. Lavrijsen, D. M. Van Ryckeghem, G. Crombez and A. W. M.Evers. (2017). Do tonic itch and pain stimuli draw attention towards their location?. 10.1155/2017/2031627

Y. Laufer and M. Elboim. (2008). Effect of burst frequency and duration of kilohertz-frequency alternating currents and of low-frequency pulsed currents on strength of contraction, muscle fatigue, and perceived discomfort. 10.2522/ptj.20080001

D. U. Jette. (1986). Effect of different forms of transcutaneous electrical nerve stimulation on experimental pain. 10.1093/ptj/66.2.187

M. Muthalib, R. Re, L. Zucchelli, S. Perrey, D. Contini, M. Caffini, L. Spinelli, G. Kerr, V. Quaresima, M. Ferrari and A. Torricelli. (2015). Effects of increasing neuromuscular electrical stimulation current intensity on cortical sensorimotor network activation: A time domain fNIRS study. 10.1371/journal.pone.0131951

J. A. Balogun. (1991). Effects of ramp time on sensory, motor and tolerance thresholds during exogenous electrical stimulation. https://pubmed.ncbi.nlm.nih.gov/1806728/

M. Teepker, M. Kunz, M. Peters, B. Kundermann, K. Schepelmann and S. Lautenbacher. (2014). Endogenous pain inhibition during menstrual cycle in migraine. 10.1002/j.1532-2149.2013.00444.x

Fujii-Abe, M. Umino, H. Fukayama and H. Kawahara. (2016). Enhancement of Analgesic Effect by Combination of Non-Noxious Stimulation and Noxious Stimulation in Humans. 10.1111/papr.12265

D. Matre, L. Hu, L. A. Viken, I. B. Hjelle, M. Wigemyr, S. Knardahl, T. Sand and K. B. Nilsen. (2015). Experimental sleep restriction facilitates pain and electrically induced cortical responses. 10.5665/sleep.5058

W. Xia, C. D. Mørch and O. K. Andersen. (2016). Exploration of the conditioning electrical stimulation frequencies for induction of long-term potentiation-like pain amplification in humans. 10.1007/s00221-016-4653-1

D. J. Crockett, K. M. Prkachin and K. D. Craig. (1977). Factors of the langugage of pain in patient and volunteer groups. 10.1016/0304-3959(77)90131-2

M. Kunz, K. E. Scholl, U. Schu and S. Lautenbacher. (2006). GABAergic modulation of diffuse noxious inhibitory controls (DNIC): a test by use of lorazepam. 10.1007/s00221-006-0558-8

A. Inoue, H. Oshita, Y. Maruyama, Y. Tanaka, Y. Ishitobi, A. Kawano, R. Ikeda, T. Ando, S. Aizawa, K. Masuda, H. Higuma, M. Kanehisa, T. Ninomiya and J. Akiyoshi. (2015). Gender determines cortisol and alpha-amylase responses to acute physical and psychosocial stress in patients with borderline personality disorder. 10.1016j.psychres.2015.04.008

I. Lund, T. Lundeberg, J. Kowalski and E. Svensson. (2005). Gender differences in electrical pain threshold responses to transcutaneous electrical nerve stimulation (TENS). 10.1016/j.neulet.2004.10.068

B. Geng, S. A. Paramanathan, K. F. Pedersen, M. V. Lauridsen, J. Gade, R. Lontis and W. Jensen. (2015). Gender effect on discrimination of location and frequency in surface electrical stimulation. 10.1109/embc.2015.7318795

K. Jung, L. E. Larsen, S. Rottmann and J. Ellrich. (2011). Heterotopic low-frequency stimulation induces nociceptive LTD within the same central receptive field in man. 10.1007/s00221-011-2718-8

A. I. M. van Laarhoven, F. W. Kraaimaat, O. H. Wilder-Smith, P. C. M. van de Kerkhof and A. W. M. Evers. (2010). Heterotopic pruritic conditioning and itch - Analogous to DNIC in pain?. 10.1016j.pain.2010.02.026

L. Vo and P. D. Drummond. (2013). High frequency electrical stimulation concurrently induces central sensitization and ipsilateral inhibitory pain modulation. 10.1002/j.1532-2149.2012.00208.x

E. N. van den Broeke and A. Mouraux. (2014). High-frequency electrical stimulation of the human skin induces heterotopical mechanical hyperalgesia, heat hyperalgesia, and enhanced responses to nonnociceptive vibrotactile input. 10.1152/jn.00651.2013

B. Vaseghi, M. Zoghi and S. Jaberzadeh. (2015). How does anodal transcranial direct current stimulation of the pain neuromatrix affect brain excitability and pain perception? A randomised, double-blind, sham-control study. 10.1371/journal.pone.0118340

Canaipa, A. Castro-Caldas, J. M. Moreira, F. Pimentel-Santos, J. C. Branco and R. Treister. (2017). Impaired Pain Modulation in Fibromyalgia Patients in Response to Social Distress Manipulation. 10.1097AJP.0000000000000447

A. Pielsticker, G. Haag, M. Zaudig and S. Lautenbacher. (2005). Impairment of pain inhibition in chronic tension-type headache. 10.1016/j.pain.2005.08.019

Watanabe, P. Svensson and L. Arendt-Nielsen. (1999). Influence of segmental and extra-segmental conditioning stimuli on cortical potentials evoked by painful electrical stimulation. 10.1080/08990229970492

H. M. Strassburg, J. U. Krainick and U. Thoden. (1977). Influence of transcutaneous nerve stimulation (TNS) on acute pain. 10.1007/BF00316311

A. R. Ward and S. Lucas-Toumbourou. (2007). Lowering of Sensory, Motor, and Pain-Tolerance Thresholds With Burst Duration Using Kilohertz-Frequency Alternating Current Electric Stimulation. 10.1016/j.apmr.2007.04.009

A. R. Ward and W. L. H. Chuen. (2009). Lowering of Sensory, Motor, and Pain-Tolerance Thresholds With Burst Duration Using Kilohertz-Frequency Alternating Current Electric Stimulation: Part II. 10.1016/j.apmr.2009.02.022

C. Babiloni, F. Babiloni, F. Carducci, F. Cincotti, F. Rosciarelli, P. M. Rossini, L. Arendt-Nielsen and A. C. N. Chen. (2001). Mapping of early and late human somatosensory evoked brain potentials to phasic galvanic painful stimulation. 10.1002/1097-0193(200103)12:3<168::AID-HBM1013>3.0.CO;2-O

S. Lang, T. Klein, W. Magerl and R.-D. Treede. (2007). Modality-specific sensory changes in humans after the induction of long-term potentiation (LTP) in cutaneous nociceptive pathways. 10.1016/j.pain.2006.09.026

P. A. Roche, K. Gijsbers, J. J. Belch and C. D. Forbes. (1984). Modification of induced ischaemic pain by transcutaneous electrical nerve stimulation. 10.1016/0304-3959(84)90809-1

H. H. Andersen, A. I. M. van Laarhoven, J. Elberling and L. Arendt-Nielsen. (2017). Modulation of itch by conditioning itch and pain stimulation in healthy humans. 10.1016/j.jpain.2017.07.002

J. Schoenen, W. Wang and P. Gerard. (1994). Modulation of temporalis muscle exteroceptive suppression by limb stimuli in normal man. 10.1016/0006-8993(94)90970-9

A. Meulders, A. Rousseau and J. W. S. Vlaeyen. (2015). Motor intention as a trigger for fear of movement-related pain: An experimental cross-US reinstatement study. 10.5127/jep.043614

S. Lautenbacher, G. B. Rollman and G. A. McCain. (1994). Multi-method assessment of experimental and clinical pain in patients with fibromyalgia. 10.1016/0304-3959(94)90046-9

M. Ozawa, K. Tsuchiyama, R. Gomi, F. Kurosaki, Y. Kawamoto and S. Aiba. (2006). Neuroselective transcutaneous electric stimulation reveals body area-specific differences in itch perception. 10.1016/j.jaad.2006.08.032

M. Ozawa, K. Tsuchiyama, R. Gomi, F. Kurosaki, Y. Kawamoto and S. Aiba. (2009). Neuroselective transcutaneous electrical stimulation reveals neuronal sensitization in atopic dermatitis. 10.1016/j.jaad.2008.11.900

R. Rukwied, A. Mayer, O. Kluschina, O. Obreja, M. Schley and M. Schmelz. (2010). NGF induces non-inflammatory localized and lasting mechanical and thermal hypersensitivity in human skin. 10.1016/j.pain.2009.11.022

Abad-Alegria, C. Pomarön, C. Aznar, C. Munoz and S. Adelantado. (2001). Objective assessment of the sympatholytic action of the nei-kuan acupoint. 10.1142/S0192415X0100023X

C. J. Patrick, K. D. Craig and K. M. Prkachin . (1986). Observer judgments of acute pain: Facial action determinants . 10.1037/0022-3514.50.6.1291

S. Lautenbacher, M. Prager and G. B. Rollman. (2007). Pain additivity, diffuse noxious inhibitory controls, and attention: a functional measurement analysis. 10.1080/08990220701637638

M. A. Giamberardino, K. J. Berkley, S. Iezzi, P. De Bigontina and L. Vecchiet. (1997). Pain threshold variations in somatic wall tissues as a function of menstrual cycle, segmental site and tissue depth in non-dysmenorrheic women, dysmenorrheic women and men. 10.1016/S0304-3959(97)03362-9

P. Schestatsky, L. Dall-Agnol, L. Gheller, L. C. Stefani, P. R. S. Sanches, I. C. de Souza, I. L. Torres and W.Caumo. (2013). Pain-autonomic interaction after work-induced sleep restriction. 10.1111/ene.12011

N. Claes, G. Crombez and J. W. S. Vlaeyen. (2015). Pain-avoidance versus reward-seeking: An experimental investigation. 10.1097/j.pain.0000000000000116

T. Klein, W. Magerl and R.-D. Treede. (2006). Perceptual Correlate of Nociceptive Long-Term Potentiation (LTP) in Humans Shares the Time Course of Early-LTP. 10.1152/jn.00755.2006

S. Delli Pizzi, D. Mantini, A. Ferretti, M. Caulo, I. Salerio, G. L. Romani, C. Del Gratta and A.Tartaro. (2010). Pharmacological functional MRI assessment of the effect of ibuprofen-arginine in painful conditions. 10.1177/039463201002300329

S. Lautenbacher and G. B. Rollman. (1997). Possible deficiencies of pain modulation in fibromyalgia. 10.109700002508-199709000-00003

D. M. Torta, M. K. Tatu, D. Cotroneo, A. Alamia, A. Folegatti and J. Trojan. (2016). Prism adaptation contrasts perceptual habituation for repetitive somatosensory stimuli. 10.1016/j.actpsy.2016.01.009

P. H. Vuilleumier, F. G. Arguissain, J. A. Biurrun Manresa, A. Y. Neziri, A. C. Nirkko, O. K. Andersen, L. Arendt-Nielsen and M. Curatolo. (2017). Psychophysical and electrophysiological evidence for enhanced pain facilitation and unaltered pain inhibition in acute low back pain patients. 10.1016/j.jpain.2017.05.008

N. Hansen, T. Klein, W. Magerl and R. D. Treede. (2007). Psychophysical evidence for long-term potentiation of C-fiber and Aδ-fiber pathways in humans by analysis of pain descriptors. 10.1152/jn.01125.2006

D. Jamner and H. Leigh. (1999). Repressive/defensive coping, endogenous opioids and health: how a life so perfect can make you sick. 10.1016/s0165-1781(98)00134-6

S. Volders, A. Meulders, S. De Peuter, B. Vervliet and J. W. S. Vlaeyen. (2012). Safety behavior can hamper the extinction of fear of movement-related pain: An experimental investigation in healthy participants. 10.1016j.brat.2012.06.004

A. Kawano, Y. Tanaka, Y. Ishitobi, Y. Maruyama, T. Ando, A. Inoue, S. Okamoto, J. Imanaga, M. Kanehisa, H. Higuma, T. Ninomiya, J. Tsuru and J. Akiyoshi. (2013). Salivary alpha-amylase and cortisol responsiveness following electrical stimulation stress in obsessive-compulsive disorder patients. 10.1016/j.psychres.2012.11.010

L. S. Chesterton, P. Barlas, N. E. Foster, T. Lundeberg, C. C. Wright and G. D. Baxter. (2002). Sensory stimulation (TENS): effects of parameter manipulation on mechanical pain thresholds in healthy human subjects. 10.1016s0304-3959(02)00118-5

A. R. Ward and V. J. Robertson. (1998). Sensory, motor, and pain thresholds for stimulation with medium frequency alternating current. 10.1016/s0003-9993(98)90006-5

R. L. Quiton and J. D. Greenspan. (2007). Sex differences in endogenous pain modulation by distracting and painful conditioning stimulation. 10.1016/j.pain.2007.09.001

A. B. Eder, D. Dignath, T. M. Erle and J. Wiemer . (2017). Shocking action: Facilitative effects of punishing electric shocks on action control . 10.1037/xge0000332

K. D. Craig and S. Coren. (1975). Signal detection analyses of social modelling influences on pain expressions. 10.1016/0022-3999(75)90057-4

M. Kucera, O. Horácek, J. Kálal, P. Kolár, P. Korbelar and Z. Polesná. (2003). Synergetic analgesic effect of the combination of arnica and hydroxyethyl salicylate in ethanolic solution following cutaneous application by transcutaneous electrostimulation. 10.1055/s-0031-1299840

W. Xia, C. D. Mørch and O. K. Andersen. (2016). Test-retest reliability of 10 Hz conditioning electrical stimulation inducing long-term potentiation (LTP)-like pain amplification in humans. 10.1371/journal.pone.0161117

M. den Hollander, A. Meulders, M. Jakobs and J. W. Vlaeyen. (2015). The effect of threat information on acquisition, extinction, and reinstatement of experimentally conditioned fear of movement-related pain. 10.1111/pme.12836
F. Zeidan, N. S. Gordon, J. Merchant and P. Goolkasian. (2010). The Effects of Brief Mindfulness Meditation Training on Experimentally Induced Pain. 10.1016/j.jpain.2009.07.015
T. Klein, S. Stahn, W. Magerl and R. D. Treede. (2008). The role of heterosynaptic facilitation in long-term potentiation (LTP) of human pain sensation. 10.1016/j.pain.2008.06.001
S. Álvarez-Blanco, L. Leon and J. Valls-Solé. (2009). The startle reaction to somatosensory inputs: Different response pattern to stimuli of upper and lower limbs. 10.1007/s00221-009-1784-7
P. Durga, S. R. Wudaru, S. K. R. Khambam, S. J. Chandra and G. Ramachandran. (2016). Validation of simple and inexpensive algometry using sphygmomanometer cuff and neuromuscular junction monitor with standardized laboratory algometer. 10.4103/0970-9185.173390
R. Lecybyl, J. Acosta, J. Ghoshdastidar, K. Stringfellow and M. Hanna. (2010). Validation, reproducibility and safety of transdermal electrical stimulation in chronic pain patients and healthy volunteers. 10.1186/1471-2377-10-5
A. R. Ward and V. J. Robertson. (1998). Variation in torque production with frequency using medium frequency alternating current. 10.1016/s0003-9993(98)90234-9
A. R. Ward, W. G. Oliver and D. Buccella. (2006). Wrist extensor torque production and discomfort associated with low-frequency and burst-modulated kilohertz-frequency currents. 10.2522/ptj.20050300
J. Lötsch, V. Dimova, A. Ultsch, I. Lieb, M. Zimmermann, G. Geisslinger and B. G. Oertel. (2016). A small yet comprehensive subset of human experimental pain models emerging from correlation analysis with a clinical quantitative sensory testing protocol in healthy subjects. 10.1002/ejp.803
O. Höffken, S. Özgül Ö, E. K. Enax-Krumova, M. Tegenthoff and C. Maier. (2017). Evoked potentials after painful cutaneous electrical stimulation depict pain relief during a conditioned pain modulation. 10.1186s12883-017-0946-7
C. Staahl, L. L. Christrup, S. D. Andersen, L. Arendt-Nielsen and A. M. Drewes. (2006). A comparative study of oxycodone and morphine in a multi-modal, tissue-differentiated experimental pain model. 10.1016j.pain.2006.02.006
Z. Katsarava, I. Ayzenberg, F. Sack, V. Limmroth, H. C. Diener and H. Kaube. (2006). A novel method of eliciting pain-related potentials by transcutaneous electrical stimulation. 10.1111/j.1526-4610.2006.00446.x
C. Christmann, C. Koeppe, D. F. Braus, M. Ruf and H. Flor. (2007). A simultaneous EEG-fMRI study of painful electric stimulation. 10.1016/j.neuroimage.2006.11.006
E. Ralevski, A. Perrino, G. Acampora, J. Koretski, D. Limoncelli and I. Petrakis. (2010). Analgesic effects of ethanol are influenced by family history of alcoholism and neuroticism. 10.1111/j.1530-0277.2010.01228.x
N. Geschwind, M. Meulders, M. L. Peters, J. W. Vlaeyen and A. Meulders. (2015). Can experimentally induced positive affect attenuate generalization of fear of movement-related pain?. 10.1016/j.jpain.2014.12.003
R. Klinger, S. Soost, H. Flor and M. Worm. (2007). Classical conditioning and expectancy in placebo hypoalgesia: a randomized controlled study in patients with atopic dermatitis and persons with healthy skin. 10.1016j.pain.2006.08.025
P. Bąbel, E. A. Bajcar, W. Adamczyk, P. Kicman, N. Lisińska, K. Świder and L. Colloca. (2017). Classical conditioning without verbal suggestions elicits placebo analgesia and nocebo hyperalgesia. 10.1371/journal.pone.0181856
A. Hahn, G. S. Kranz, E.-M. Seidel, R. Sladky, C. Kraus, M. Küblböck, D. M. Pfabigan, A. Hummer, A. Grahl, S. Ganger, C. Windischberger, C. Lamm and R. Lanzenberger. (2013). Comparing neural response to painful electrical stimulation with functional MRI at 3 and 7T. 10.1016/j.neuroimage.2013.06.010
W. Freund, G. Stuber, A. P. Wunderlich and B. Schmitz. (2007). Cortical correlates of perception and suppression of electrically induced pain. 10.1080/08990220701723636
L. Wang, A. C. N. Chen and L. Arendt-Nielsen. (2006). Cortical plasticity: Effect of high and low intensity conditioning electrical stimulations (100 Hz) on SEPs to painful finger stimulation. 10.1016/j.clinph.2006.01.007
M. Koltzenburg, R. Pokorny, U. E. Gasser and U. Richarz. (2006). Differential sensitivity of three experimental pain models in detecting the analgesic effects of transdermal fentanyl and buprenorphine. 10.1016j.pain.2006.06.028
H. Schulte, A. Sollevi and M. Segerdahl. (2005). Dose-dependent effects of morphine on experimentally induced cutaneous pain in healthy volunteers. 10.1016/j.pain.2005.05.005
F. D'Agata, A. Cicerale, A. Mingolla, P. Caroppo, L. Orsi, P. Mortara, W. Troni and L. Pinessi. (2015). Double-cone coil TMS stimulation of the medial cortex inhibits central pain habituation. 10.1371/journal.pone.0128765
M. Shimojo, P. Svensson, L. Arendt-Nielsen and A. C. N. Chen. (2000). Dynamic brain topography of somatosensory evoked potentials and equivalent dipoles in response to graded painful skin and muscle stimulation. 10.1023A:1007834319135
V. Ružić, D. Ivanec and K. Modić Stanke. (2017). Effect of expectation on pain assessment of lower- and higher-intensity stimuli. 10.1016/j.sjpain.2016.09.013
A. Castro, P. Amorim, C. S. Nunes and F. G. de Almeida. (2015). Effect of propofol and remifentanil on a somatosensory evoked potential indicator of pain perception intensity in volunteers. 10.1007s10877-014-9632-0
T. Andresen, C. Staahl, A. Oksche, H. Mansikka, L. Arendt-Nielsen and A. M. Drewes. (2011). Effect of transdermal opioids in experimentally induced superficial, deep and hyperalgesic pain. 10.1111/j.1476-5381.2010.01180.x
G. Landmann, L. Stockinger, C. Lustenberger, M. Schmelz and R. Rukwied. (2016). Effects of Current Density on Nociceptor Activation Upon Electrical Stimulation in Humans. 10.1111/papr.12339
F. T. Nickel, S. Ott, S. Möhringer, T. Münster, S. Rieß, J. Filitz, W. Koppert and C. Maihöfner. (2016). Effects of Different Anesthetics on Pain Processing in an Experimental Human Pain Model. 10.1111/papr.12326
O. Vassend and S. Knardahl. (2005). Effects of repeated electrocutaneous pain stimulation on facial blood flow. 10.1016/j.biopsycho.2004.04.004
H. Kang, A. Nakae, H. Ito, P. Vitayaburananont, T. Minamoto, T. Ikeda, M. Osaka, T. Mashimo, Y. Fujino and S. Hagihira. (2017). Effects of sedation on subjective perception of pain intensity and autonomic nervous responses to pain: A preliminary study. 10.1371/journal.pone.0183635
K. Jung, D. Lelic, S. Rottmann, A. M. Drewes, L. Petrini and J. Ellrich. (2012). Electrical low-frequency stimulation induces central neuroplastic changes of pain processing in man. 10.1016/j.ejpain.2011.08.006
S. Rottmann, K. Jung and J. Ellrich. (2008). Electrical low-frequency stimulation induces homotopic long-term depression of nociception and pain from hand in man. 10.1016/j.clinph.2008.02.022
M. Wilkinson, D. McIntyre and L. Edwards. (2013). Electrocutaneous pain thresholds are higher during systole than diastole. 10.1016/j.biopsycho.2013.05.002
A. C. Perrino, Jr., E. Ralevski, G. Acampora, J. Edgecombe, D. Limoncelli and I. L. Petrakis. (2008). Ethanol and pain sensitivity: Effects in healthy subjects using an acute pain paradigm. 10.1111/j.1530-0277.2008.00653.x
P. H. Vuilleumier, M. Besson, J. Desmeules, L. Arendt-Nielsen and M. Curatolo. (2013). Evaluation of anti-hyperalgesic and analgesic effects of two benzodiazepines in human experimental pain: a randomized placebo-controlled study. 10.1371/journal.pone.0043896
M. de Brito Barbosa, E. C. de Oliveira Guirro and F. R. Nunes. (2013). Evaluation of sensitivity, motor and pain thresholds across the menstrual cycle through mediumfrequency transcutaneous electrical nerve stimulation. 10.6061/clinics/2013(07)03
R. Canaipa, R. Treister, M. Lang, J. M. Moreira and A. C. Caldas. (2016). Feeling hurt: Pain sensitivity is correlated with and modulated by social distress. 10.1097/AJP.0000000000000220
Y. Oshiro, N. Fuijita, H. Tanaka, N. Hirabuki, H. Nakamura and I. Yoshiya. (1998). Functional mapping of pain-related activation with echo-planar MRI: significance of the SII-insular region. 10.109700001756-199807130-00026
W. Freund, A. P. Wunderlich, G. Stuber, B. Landwehrmeyer and R. Klug. (2010). Graded cutaneous electrical vs thermal stimulation in humans shows different insular and cingulate cortex activation. 10.310908990220903516593
G. Crombez, C. Eccleston, F. Baeyens and P. Eelen. (1997). Habituation and the interference of pain with task performance. 10.1016/s0304-3959(96)03304-0
S. Li, T. Hu, M. A. Beran and S. Li. (2014). Habituation to experimentally induced electrical pain during voluntary-breathing controlled electrical stimulation (BreEStim). 10.1371/journal.pone.0104729
F. Fardo, C. Spironelli and A. Angrilli. (2013). Horizontal body position reduces cortical pain-related processing: evidence from late ERPs. 10.1371/journal.pone.0081964
K. Świder, E. Wronka, J. M. Oosterman, C. M. van Rijn and M. L. A. Jongsma. (2017). Influence of transient spatial attention on the P3 component and perception of painful and non-painful electric stimuli in crossed and uncrossed hands positions. 10.1371/journal.pone.0182616
F. T. Nickel, R. DeCol, S. Jud, P. A. Fasching, F. Seifert and C. Maihöfner. (2011). Inhibition of hyperalgesia by conditioning electrical stimulation in a human pain model. 10.1016/j.pain.2011.02.005
N. Takakura, H. Yajima and I. Homma. (2004). Inhibitory effect of pain-eliciting transcutaneous electrical stimulation on vibration-induced finger flexion reflex in the human upper limb. 10.2170/jjphysiol.54.243
A. P. Tucker, Y. I. Kim, R. Nadeson and C. S. Goodchild. (2005). Investigation of the potentiation of the analgesic effects of fentanyl by ketamine in humans: A double-blinded, randomised, placebo controlled, crossover study of experimental pain[ISRCTN83088383]. 10.1186/1471-2253-5-2
W. Karlson, N. A. Hamilton and M. A. Rapoff. (2014). Massage on experimental pain in healthy females: A randomized controlled trial. 10.1177/1359105312471572
P. S. Boggio, S. Zaghi, M. Lopes and F. Fregni. (2008). Modulatory effects of anodal transcranial direct current stimulation on perception and pain thresholds in healthy volunteers. 10.1111/j.1468-1331.2008.02270.x
M. N. Janal, W. C. Clark and J. D. Carroll. (1991). Multidimensional scaling of painful and innocuous electrocutaneous stimuli: Reliability and individual differences. 10.3758/BF03212212
M. Rance, M. Ruttorf, F. Nees, L. R. Schad and H. Flor. (2014). Neurofeedback of the difference in activation of the anterior cingulate cortex and posterior insular cortex: Two functionally connected areas in the processing of pain. 10.3389/fnbeh.2014.00357
N. Janal, M. Glusman, J. P. Kuhl and W. C. Clark. (1994). On the absence of correlation between responses to noxious heat, cold, electrical and ischemic stimulation. 10.1016/0304-3959(94)90135-x
J. W. Ibinson, K. M. Vogt, K. B. Taylor, S. B. Dua, C. J. Becker, M. Loggia and A. D. Wasan. (2015). Optimizing and interpreting insular functional connectivity maps obtained during acute experimental pain: The effects of global signal and task paradigm regression. 10.1089/brain.2015.0354
F. Godinho, M. Frot, C. Perchet, M. Magnin and L. Garcia-Larrea. (2008). Pain influences hedonic assessment of visual inputs. 10.1111/j.1460-9568.2008.06196.x
Hu, S. Li and S. Li. (2015). Pain modulation effect of breathing-controlled electrical stimulation (BreEStim) is not likely to be mediated by deep and fast voluntary breathing. 10.1038/srep14228
C. Günther, A. R. Schandl, J. Berhardsson, A. Bjärtå, M. Wållgren, Ö. Sundin, J. Alvarsson, M. Bottai, C. R.Martling and P. V. Sackey. (2016). Pain rather than induced emotions and ICU sound increases skin conductance variability in healthy volunteers. 10.1111/aas.12751
C. H. Röder, M. Michal, G. Overbeck, V. G. van de Ven and D. E. J. Linden. (2007). Pain Response in Depersonalization: A Functional Imaging Study Using Hypnosis in Healthy Subjects. 10.1159/000097970
J. P. Lefaucheur, R. Ahdab, S. S. Ayache, I. Lefaucheur-Ménard, D. Rouie, D. Tebbal, D. O. Neves and D. Ciampi de Andrade. (2012). Pain-related evoked potentials: a comparative study between electrical stimulation using a concentric planar electrode and laser stimulation using a CO2 laser. 10.1016/j.neucli.2011.12.003
S. T. Au Yeung, B. Colagiuri, P. F. Lovibond and L. Colloca. (2014). Partial reinforcement, extinction, and placebo analgesia. 10.1016/j.pain.2014.02.022
R. V. Juul, D. J. Foster, R. N. Upton, T. Andresen, C. Graversen, A. M. Drewes, L. L. Christrup and M.Kreilgaard. (2014). Pharmacodynamic modelling of placebo and buprenorphine effects on event-related potentials in experimental pain. 10.1111/bcpt.12217
E. N. van den Broeke, B. de Vries, J. Lambert, D. M. Torta and A. Mouraux. (2017). Phase-locked and non-phase-locked EEG responses to pinprick stimulation before and after experimentally-induced secondary hyperalgesia. 10.1016/j.clinph.2017.05.006
N. Sitaram, M. S. Buchsbaum and J. C. Gillin. (1977). Physostigmine analgesia and somatosensory evoked responses in man. 10.1016/0014-2999(77)90295-3
T. J. Neddermeyer, K. Flühr and J. Lötsch. (2008). Principle components analysis of pain thresholds to thermal, electrical, and mechanical stimuli suggests a predominant common source of variance. 10.1016j.pain.2007.12.015
M. Rance, M. Ruttorf, F. Nees, L. R. Schad and H. Flor. (2014). Real time fMRI feedback of the anterior cingulate and posterior insular cortex in the processing of pain. 10.1002/hbm.22585
A. J. van Wijk, F. Lobbezoo and J. Hoogstraten. (2013). Reliability and validity of a continuous pain registration procedure. 10.1002/j.1532-2149.2012.00194.x
A. I. M. Van Laarhoven, F. W. Kraaimaat, O. H. Wilder-Smith and A. W. M. Evers. (2010). Role of attentional focus on bodily sensations in sensitivity to itch and pain. 10.2340/00015555-0743
E. M. van der Heide, J. R. Buitenweg, E. Marani and W. L. Rutten. (2009). Single pulse and pulse train modulation of cutaneous electrical stimulation: a comparison of methods. 10.1097/WNP.0b013e3181942cd2
E. Reid, D. Harvie, R. Miegel, C. Spence and G. L. Moseley. (2015). Spatial summation of pain in humans investigated using transcutaneous electrical stimulation. 10.1016/j.jpain.2014.10.001
Gatzounis, R., Schrooten, M. G. S., Crombez, G., Vancleef, L. M. G., & Vlaeyen, J. W. S.. (2017). Taking a break in response to pain. An experimental investigation of the effects of interruptions by pain on subsequent activity resumption. 10.1016/j.sjpain.2017.02.008
A. Meulders, D. Vansteenwegen and J. W. S. Vlaeyen. (2011). The acquisition of fear of movement-related pain and associative learning: A novel pain-relevant human fear conditioning paradigm. 10.1016/j.pain.2011.05.015
M. I. Johnson, V. K. Hajela, C. H. Ashton and J. W. Thompson. (1991). The effects of auricular transcutaneous electrical nerve stimulation (TENS) on experimental pain threshold and autonomic function in healthy subjects. 10.1016/0304-3959(91)90116-F
L. M. G. Vancleef and M. L. Peters. (2011). The influence of perceived control and self-efficacy on the sensory evaluation of experimentally induced pain. 10.1016/j.jbtep.2011.05.006
L. Colloca, M. Sigaudo and F. Benedetti. (2008). The role of learning in nocebo and placebo effects. 10.1016j.pain.2008.02.006
D. S. Harvie, A. Meulders, V. J. Madden, S. L. Hillier, D. K. Peto, R. Brinkworth and G. L. Moseley. (2016). When touch predicts pain: Predictive tactile cues modulate perceived intensity of painful stimulation independent of expectancy. 10.1016/j.sjpain.2015.09.007
A. Meulders, D. Vansteenwegen and J. W. S. Vlaeyen. (2012). Women, but not men, report increasingly more pain during repeated (un)predictable painful electrocutaneous stimulation: Evidence for mediation by fear of pain. 10.1016/j.pain.2012.02.005
M. P. Bennett, A. Meulders, F. Baeyens and J. W. S. Vlaeyen. (2015). Words putting pain in motion: The generalization of pain-related fear within an artificial stimulus category. 10.3389/fpsyg.2015.00520

No full text available/text is not article/duplicate/text in a language other than English

Seno, S., Kato, S., Kobayashi, H., Watanabe, A., & Shimazu, H. (2011). Evaluation of sex and age differences in the perception threshold of body surface against electrical stimulation-measurement of perception threshold to determine the possibility of diabetic neuropathy diagnoses. Japanese Soc Med Biol Eng, 49(1), 163-169.
Roytman, T. M., Kitagawa, K. M., & Yamamoto, L. G. (2006). Minimum perceived VAS differences at an interval of 7 days. The Pain Clinic, 18(2), 119-121.
Doğuluer, M., Gurses, H. N., Demir, R., & Ozyilmaz, S. (2003). Electrical pain threshold in patients with symptomatic and asymptomatic ischemic heart disease and healthy subjects. The Pain Clinic, 15(4), 381-384.
Kinnunen, M., & Alasaarela, E. (2004). Registering the response of tissues exposed to an interferential electric current stimulation. Acupuncture & Electro-Therapeutics Research, 29(3-4), 213-226.
Stacher, G., Bauer, P., Ehn, I., & Schreiber, E. (1979). Effects of tolmetin, paracetamol, and of two combinations of tolmetin and paracetamol as compared to placebo on experimentally induced pain. A double blind study. International Journal of Clinical Pharmacology and Biopharmacy, 17(6), 250-255.
Dragani, L., Giamberardino, M. A., Obletter, G., & Vecchiet, L. (1988). Effects of diflunisal on cutaneous sensory and pain thresholds. Clinical Therapeutics, 10(4), 388-391.
Poulsen, A. H., van den Berg, B., Arguissain, F., Tigerholm, J., Buitenweg, J. R., Andersen, O. K., & Mørch, C. D. (2022). Novel surface electrode design for preferential activation of cutaneous nociceptors. Journal of neural engineering, 19(1), 016010.
S. I. Seno, S. Kato, H. Kobayashi, A. Watanabe and H. Shimazu. (2011). Evaluation of sex and age differences in the perception threshold of body surface against electrical stimulation-measurement of perception threshold to determine the possibility of diabetic neuropathy diagnoses. https://www.scopus.com/inwardrecord.uri?eid=2-s2.0-83455231422&partnerID=40&md5=10a505896c68574338456cbb2667725c
Brown, Mary Cleveland; Schaffer, Randy L.; Donaldson, Gary; Shen, Danny D. (1995). A pharamacokinetic approach to resolving spinal and systemic contributions to epidural alfentanil analgesia and side-effects. 10.1016/0304-3959(95)00008-G
G. Jess, E. M. Pogatzki-Zahn, P. K. Zahn and C. H. Meyer-Frießem. (2016). Monitoring heart rate variability to assess experimentally induced pain using the analgesia nociception index: A randomised volunteer study. 10.1097eja.0000000000000304
M. Richter, C. Schroeter, T. Puensch, T. Straube, H. Hecht, A. Ritter, W. H. R. Miltner and T.Weiss. (2014). Pain-related and negative semantic priming enhances perceived pain intensity. 10.1155/2014425321
S. B. McDonald, S. S. Liu, D. J. Kopacz and C. A. Stephenson. (1999). Hyperbaric spinal ropivacaine: a comparison to bupivacaine in volunteers. 10.1097/00000542-199904000-00007
E. Scelzo, G. Giannicola, M. Rosa, M. Ciocca, G. Ardolino, F. Cogiamanian, R. Ferrucci, M. Fumagalli, F. Mameli, S. Barbieri and A. Priori. (2011). Increased short latency afferent inhibition after anodal transcranial direct current stimulation. 10.1016/j.neulet.2011.05.007
E. Kosek and A. Januszewska. (2008). Mechanisms of pain referral in patients with whiplash associated disorder. 10.1016/j.ejpain.2007.10.006
Kanda M, Fujiwara N, Xu X, Shindo K, Nagamine T, Ikeda A, Shibasaki H. (1996). Pain-related and cognitive components of somatosensory evoked potentials following CO2 laser stimulation in man. 10.1016/0013-4694(95)00190-5
Y. Kitamura, R. Kakigi, M. Hoshiyama, S. Koyama, M. Shimojo and S. Watanabe. (1995). Pain-related somatosensory evoked magnetic fields. 10.1016/0013-4694(95)00139-5
Z. Gunendi, M. Polat, D. Vuralli and B. Cengiz. (2019). Somatosensory temporal discrimination is impaired in fibromyalgia. 10.1016/j.jocn.2018.10.067
A. F. Gonter and D. J. Kopacz. (2005). Spinal 2-chloroprocaine: a comparison with procaine in volunteers. 10.121301.Ane.0000143380.36298.4a
J. R. Yoos and D. J. Kopacz. (2005). Spinal 2-chloroprocaine: a comparison with small-dose bupivacaine in volunteers. 10.1213/01.Ane.0000143356.17013.A1
D. J. Kopacz. (2005). Spinal 2-chloroprocaine: minimum effective dose. 10.1016/j.rapm.2004.09.008
A. Westgeest, M. Morales, C. Cabib and J. Valls-Sole. (2014). The effects of transcranial direct current stimulation on conscious perception of sensory inputs from hand palm and dorsum. 10.1111/ejn.12743
A. Tröster, R. Sittl, B. Singler, M. Schmelz, J. Schüttler and W. Koppert. (2006). Modulation of remifentanil-induced analgesia and postinfusion hyperalgesia by parecoxib in humans. 10.1097/00000542-200611000-00024
H.C.J.L. Buscher a, H. van Goor a, O.H.G. Wilder-Smith. (2007). Effect of thoracoscopic splanchnic denervation on pain processing in chronic pancreatitis patients. 10.1016/j.ejpain.2006.06.001
R. Romberg, E. Olofsen, E. Sarton, J. den Hartigh, P. E. Taschner and A.Dahan. (2004). Pharmacokinetic-pharmacodynamic modeling of morphine-6-glucuronide-induced analgesia in healthy volunteers: absence of sex differences. 10.1097/00000542-200401000-00021
Mouraux, A., Bloms-Funke, P., Boesl, I., Caspani, O., Chapman, S. C., Di Stefano, G., Finnerup, N. B., Garcia-Larrea, L., Goetz, M., Kostenko, A., Pelz, B., Pogatzki-Zahn, E., Schubart, K., Stouffs, A., Truini, A., Tracey, I., Troconiz, I. F., Van Niel, J., Vela, J. M., Vincent, K., ... Treede, R. D. (2021). IMI2-PainCare-BioPain-RCT3: a randomized, double-blind, placebo-controlled, crossover, multi-center trial in healthy subjects to investigate the effects of lacosamide, pregabalin, and tapentadol on biomarkers of pain processing observed by electroencephalography (EEG). 10.1186/s13063-021-05272-y
E. L. Terry. (2016). Does experimental reduction of pain catastrophizing modulate spinal nociception?. https://www.proquest.com/docview/1729448931
J. S. Walker and J. J. Carmody. (1998). Experimental pain in healthy human subjects: gender differences in nociception and in response to ibuprofen. 10.1097/00000539-199806000-00023
Andersson, S. A.; Ericson, T.; Holmgren, E.; Lindqvist, G. (1979). Analgesic effects of peripheral conditioning stimulation: I General pain threshold effects on human teeth and a correlation to psychological factors. 10.3727/036012977817553358
C. A. Manzler, M. Radoman, K. J. Khorrami and S. M. Gorka. (2022). Association between startle reactivity to uncertain threats and structural brain volume. 10.1111/psyp.14074
Miltner, Wolfgang; Larbig, Wolfgang; Braun, Christoph. (1988). Attention and event-related potentials elicited by intracutaneous electrical stimulation of the skin. https://psycnet.apa.org/record/1989-28616-001
J. Opavský, M. Dostálek and R. Marácek. (1991). Autonomic and verbal responses to the experimental nociceptive stimulation. https://pubmed.ncbi.nlm.nih.gov/1840337/
B. Bromm, I. Rundshagen and E. Scharein. (1991). Central analgesic effects of acetylsalicylic acid in healthy men. https://pubmed.ncbi.nlm.nih.gov/1810257/
T. Weggen, H. J. Schindler, B. Kordass and A. Hugger. (2013). Clinical and electromyographic follow-up of myofascial pain patients treated with two types of oral splint: a randomized controlled pilot study. https://pubmed.ncbi.nlm.nih.gov/24364193/
M. F. Levin and C. W. Y. Hui-Chan. (1993). Conventional and acupuncture-like transcutaneous electrical nerve stimulation excite similar afferent fibers. https://www.scopus.com/inwardrecord.uri?eid=2-s2.0-0027510915&partnerID=40&md5=666d57623498dddfb1e319a96acb915a
E. J. Rogers and B. Vilkin. (1978). Diurnal variation in sensory and pain thresholds correlated with mood states. https://pubmed.ncbi.nlm.nih.gov/565353/
E. Søfteland, G. Dimcevski, C. Graversen, B. G. Nedreb, A. M. Drewes and J. B. Frkjr. (2011). Effects of isolated hyperinsulinaemia on sensory function in healthy adults. 10.1055/s-0031-1286316
G. Stacher, P. Bauer, I. Ehn and E. Schreiber. (1979). Effects of tolmetin, paracetamol, and of two combinations of tolmetin and paracetamol as compared to placebo on experimentally induced pain. A double blind study. https://pubmed.ncbi.nlm.nih.gov/381221/
M. Doğuluer, H. N. Gurses, R. Demir and S. Ozyilmaz. (2003). Electrical pain threshold in patients with symptomatic and asymptomatic ischemic heart disease and healthy subjects. 10.1163/156856903770196737
J. Chu and I. Schwartz. (2008). eToims twitch relief method in Chronic Refractory Myofascial Pain (CRMP). https:www.scopus.com/inwardrecord.uri?eid=2-s2.0-53249098926&partnerID=40&md5=fba2a61966d5c84c6edb759f750ea8b2
A. López-Fiesco, M. Luján, J. M. Alvarez, G. Zamora Lopez and E. L. Martínez. (1992). Evaluation of the analgesic efficacy of a combination of naproxen and paracetamol versus dipyrone on the tourniquet test in healthy volunteers. https://pubmed.ncbi.nlm.nih.gov/1502225/
F. Papi, S. Ghione, C. Rosa, C. Del Seppia and P. Luschi. (1995). Exposure to oscillating magnetic fields influences sensitivity to electrical stimuli. II. Experiments on humans. 10.1002/bem.2250160505
R. M. Cohen, W. E. Semple, M. Gross, H. H. Holcomb, M. S. Dowling and T. E. Nordahl. (1988). Functional localization of sustained attention: Comparison to sensory stimulation in the absence of instruction. https:www.scopus.com/inwardrecord.uri?eid=2-s2.0-0024150407&partnerID=40&md5=4d9501bf4456347fc514d16cff84c8e0
H. Dou, Y. Lei, Y. Pan, H. Li and P. Astikainen. (2023). Impact of observational and direct learning on fear conditioning generalization in humans. 10.1016/j.pnpbp.2022.110650
Roytman, T. M., Kitagawa, K. M., & Yamamoto, L. G. . (2006). Minimum perceived VAS differences at an interval of 7 days. . 10.1163/156856906776760399
G. Umutlu, N. Demirci, A. T. Erdoğan, N. E. Acar and Ş. B. Fidanci. (2020). Neuromuscular, hormonal and cardiovascular adaptations to eight-week HIIT and continuous aerobic training combined with neuromuscular electrical stimulation. 10.23736/S0022-4707.19.10277-0
G. D'Aleo, E. Sessa, P. D'Aleo, C. Rifici, P. Di Bella, M. Petix and P. Bramanti. (1999). Nociceptive R3 reflex in relapsing-remitting multiple sclerosis patients. https://pubmed.ncbi.nlm.nih.gov/10321329/
P. S. Micalos, J. Harris, E. J. Drinkwater, J. Cannon and F. E. Marino. (2015). Perceptual and cerebro-spinal responses to graded innocuous and noxious stimuli following aerobic exercise. https://www.scopus.com/inwardrecord.uri?eid=2-s2.0-84962599244&partnerID=40&md5=196909f3c5e67a4f9b831dbad56067b1
P. L. Duhamel. (2003). Physiological assessment of delayed onset muscle soreness. https://www.proquest.com/openview/b12bf1a9332e932ce1c39601f0931e0b/1?pq-origsite=gscholar&cbl=18750&diss=y
M. Kinnunen and E. Alasaarela. (2004). Registering the response of tissues exposed to an interferential electric current stimulation. 10.3727/036012904815901407
P. Beltrá, I. Ruiz-del-Portal, F. J. Ortega, R. Valdesuso, M. Delicado-Miralles and E. Velasco. (2022). Sensorimotor effects of plasticity-inducing percutaneous peripheral nerve stimulation protocols: a blinded, randomized clinical trial. 10.1002/ejp.1928
K. Taniguchi, S. Yoshitake, H. Iwasaka, N. Honda and T. Oyama. (1995). The effects of imipramine, amitriptyline and clonidine administered by iontophoresis on the pain threshold. https://pubmed.ncbi.nlm.nih.gov/8669217/
Valentina Jelinčić 1, Diana M Torta 2, Ilse Van Diest 3, Andreas von Leupoldt 4. (2022). The effects of unpredictability and negative affect on perception and neural gating in different interoceptive modalities. 10.1016/j.biopsycho.2022.108267
E. J. Bartley. (2013). The relationship between sex hormones and nociceptive processing in premenstrual dysphoric disorder. https://www.proquest.com/openview/6cde843fea014621d711c9d23d6ff9b8/1?pq-origsite=gscholar&cbl=18750
J. E. Davidson, A. Verderber, V. Lake, M. Weinger, B. Partridge, B. Munden and J. Taylor. (1994). Ulnar peripheral nerve stimulation by train-of-four technique in adult nonmedicated patients. https://pubmed.ncbi.nlm.nih.gov/7920955/
B. Bromm and R. D. Treede. (1980). Withdrawal reflex, skin resistance reaction and pain ratings due to electrical stimuli in man. 10.1016/0304-3959(80)90048-2
Biya Tang, Evan Livesey, Ben Colagiuri. (2024). Choice Enhances Placebo Hypoalgesia More in Weaker Placebo Contexts: A Partial Reinforcement Study. 10.1016/j.JPAIN.2023.08.003
Fabrizio Benedetti, Claudia Arduino, Aziz Shaibani, Wilma Thoen. (2023). Creating Placebo Nonresponders in the Lab,. 10.1016/j.JPAIN.2023.10.017
Yun Zhuang, Ke Zhao, Xiaolan Fu,. (2024). The temporal effect of uncertain context on the perceptual processing of PAINful and non-PAINful STIMULation,. 10.1016/j.biopsycho.2023.108729

Supplementary Table 3 A detailed description of the studies included in the review

Articles	Type of study	Sample size	Methods used for calibration	What instructions were given to the participants	Stimuli details (e.g., single stimulus, series of stimuli)	Length of the stimuli	Length of the inter-stimulus interval	Total number of stimuli per calibration	Repetition of the calibration procedure	Length of the intervals between successive parts of the calibration	Mean pain ratings / target during calibration	Mean pain ratings during pretest/baseline	Type of pain assessment scale (e.g., VAS, NRS, VRS, VNS)	Calibration stimuli vs experiment/manipulation stimuli?	Additional commentary
[32]	single-arm	26 (11 males)	increasing calibration	**no data**	single pulse	2 ms	**no data**	different for each participant	no	n/a	target was 4/10 “just beginning to feel pain”	**no pretest/baseline**	VRS ranging from 0 (“no feeling”) to 10 (“the most intense pain imaginable”)	**no data**	The calibration was increased by 0.5 mA.
[41]	single-arm	21 (11 males)	the method of limits (increasing and decreasing)	participants were asked to press a button when they became aware of the presence or absence of the stimulus	single pulse	2 ms	**no data**	differently for each participant	3 times	**no data**	target: 10 times the perception threshold or a minimum of 1.5 mA	**no pretest/baseline**	no scale	**no**	The average of three upper (amplitude increased 5% from the sensed stimulus) and three lower (amplitude decreased 5% from the sensed stimulus) values was calculated as the perception threshold.
[73]	randomized	39 (22 males)	increasing calibration	**no data**	**no data**	**no data**	**no data**	differently for each participant	no	n/a	target was 5/10	**no data**	VRS ranging from 0, indicating “no pain”, to 10, indicating “extreme pain”	**no data**	The electrical stimulus intensity started at 1 mA and was incrementally increased by 0.1 mA until moderate pain was reported (pain level of 5 on a 0-10 scale).
[15]	randomized	60 (28 males)	increasing calibration	participants were asked to inform the experimenter as soon as they started to feel any sensation	**no data**	200 μs	5 s	differently for each participant (mean number = 14)	2 times	**no data**	tactile sensation (t)	**no data**	NRS ranging from 0 = “no pain” to 10 = “maximum imaginable pain”	**no data**	Calibration with increasing intensity increased by 1 ma, starting at 0.
											the pain threshold (T)				The stimuli of increasing intensity were applied until the participant signaled that he or she had started to feel pain (T)
											2T				The mean intensity of the pain threshold was doubled (2T) and used in the testing phase.
[14]	randomized	26 (10 males)	an adaptive staircase approach (increasing calibration)	participants were informed that the electrical stimulus would “feel like a very tiny pinprick”. participants were asked to say “yes” if they felt it, even slightly	single stimuli	2 ms	**no data**	differently for each participant	**no data**	**no data**	target: 10 times the electrical detection threshold	**no data**	no scale	**no**	The stimulus was calibrated to their individual electrical detection threshold on both arms.
															The intensity started at 0 and slowly increased in 0.1 mA increments until the participants reported that they could feel the electrical stimulus.

[58]	single-arm	67 (22 males)	the method of limits (increasin g and decreasing)	**no data**	single pulses	* 2 ms	* 9s	differently for each participant	no	n/a	target: 10 times this detection threshold	**no pretest/baseline**	NRS extending from 0—not felt at all—to 100—maximal pain—, 50 being defined as the transition from a non-painful stimulation to a painful one	**no**	Starting from 0.4 mA, the intensity of the electrical stimulus was gradually decreased or increased by steps of 0.01 mA, depending on whether the stimulus was perceived or not, up to several reversals around a stable value that was considered as the absolute detection threshold.<br>Stimuli were delivered at an intensity corresponding to 10 times this detection threshold.
[22]	randomized	88 (44 males)	increasing calibration	**no data**	**no data**	**no data**	5 s	differently for each participant	2 times	**no data**	target: detection thresholds (dt) and the value between the detection (dt) and pain thresholds (pt): dt + 0.75(pt–dt and dt+0.5(pt-dt)	0.1 (0.00–0.33); 0.03 (0.00–0.27); 0 (0.00–0.20); 0.13 (0.00–0.20)	**no data**	**no**	A series of electrical pulses of increasing intensity was provided (starting from 0 mA, increasing by 1 mA) until dt and pt were reached, which stopped the stimuli application.<br>Calibration procedure was carried out twice and the obtained results were averaged.<br>The intensity of the innocuous stimuli was calculated using the following formulas: I. dt + 0.75(pt–dt), II. dt+0.5(pt-dt) and III. dt (formulas no. I and no. II were 75% and 50% of the distance between the dt and pt, respectively; formula no. III was equal to dt).<br>The most sensitive participants (those in whom the calibration was not successful) were excluded from the study.
[44]	single-arm	16 (7 males)	**no data**	**no data**	**no data**	10 ms	**no data**	**no data**	**no data**	**no data**	target: 7/10 (smax) to define 4 stimulation intensities by varying the pulse amplitude to 25% (“level 1”), 50% (“level 2”), 75% (“level 3”) and 100% (“level 4”) of smax	**no data**	VAS from 0 to 10	**no data**	Lots of missing data, not sure how the pain was calibrated on 7/10
													not clear what kind of scale		

[76]	single-arm	35 (13 males)	increasing calibration	**no data**	**no data**	4-10 ms (differently for each participant)	**no data**	differently for each participant	**no data**	**no data**	target: 25 and 75 on 100	**no pretest/baseline**	from 0 (not painful at all) to 100 (worst pain imaginable), with a rating of 50 representing the painfulness threshold	**no data**	Calibration started with 0.2 mA and increased in steps of 0.2 mA.
[23]	randomized	157 (73 males)	**no data**	**no data**	**no data**	500 ms	**no data**	differently for each participant	3 times	**no data**	target: pain threshold (1 on NRS)	nh 2.59 (0.27); ne 3.01 (0.25); p5 2.43 (0.22); p15 2.52 (0.21); p30 2.59 (0.24); n5 2.46 (0.22); n15 2.78 (0.23); n30 3.23 (0.27);	NRS (0 representing no pain; 1, the beginning of a painful sensation; 5, moderate; and 10, unbearable pain)	**no data**	Pain threshold was determined as the average of 3 measurements.
[20]	single-arm	24 (10 males)	a staircase procedure (ascending and descending)	**no data**	single stimuli	2 ms	**no data**	differently for each participant	6 times (three ascending and three descending)	**no data**	target: 20 x detection threshold	**no pretest/baseline**	**no data**	yes	A staircase procedure with three ascending and descending staircases of single stimuli.<br>The final electrical detection threshold was the geometric mean of the three series.<br>The order with which the electrical detection thresholds were determined for each electrode was counterbalanced across participants.<br>The single electrical stimuli were delivered at an intensity of 10 times the electrical detection threshold.
[72]	randomized	60 (30 males)	the method of limits (increasing and decreasing)	**no data**	single stimuli	2 ms	**no data**	differently for each participant	4 times (two ascending and two descending)	**no data**	target: 10 x detection threshold	**no pretest/baseline**	no scale	**no**	The stimulus intensity, starting at 0.1 mA, increased in steps of 0.1 mA until the participant perceived the stimulus, and then decreased in steps of 0.05 mA until the stimulus was no longer perceived. This procedure was then repeated.<br>Stimulus intensity was defined as the geometric mean of the four measurements and then determined hfs as its 10 times.
						80 ms					3/9				Calibrations were manipulated via

[47]	randomized	247 (? males)	ascending calibration (1 ascending current voltage, 2 ascending time)	**no data**	**no data**	80 ms to 800 ms (increasing in sequence at multiples of 80 ms)	**no data**	differently for each participant	no	n/a	3/9, 5/9, 7/9	**no data**	NRS, 1 a little pain, 5 moderate pain, and 9 unbearable pain	**no**	ascending voltage of the electric currents with a fixed delivering duration of 80 ms. Once the low, moderate and high pain levels for each participant were determined, the participants were tested for rating response consistency. A random sequence of three low- and three high-intensity pain stimuli was administered.
[1]	single-arm	12 (7 males)	increasing calibration	**no data**	series of stimuli (4)	2 s	8 s	differently for each participant	**no data**	**no data**	1 mA less than stimulus inducing pain of 10/10 or 3 mA greater than the intensity reaching the angle of maximum voluntary movement of the wrist	**no pretest/baseline**	11-point ranging from 0 (no pain) to 10 (unbearable pain).	yes	From 1 mA to endurable value.
[59]	single-arm	16 (? males)	**no data**	**no data**	**no data**	10 ms	**no data**	**no data**	**no data**	**no data**	target: 7/10 (and determining 25%, 50%, 75% and 100% based on it)	**no pretest/baseline**	11-point VAS (0-no pain – 10 – worst pain imaginable) with scale numbers, colours and icons of face expressions	**no data**	Lots of missing data, not sure how the pain was calibrated on 7/10.
[46]	randomized	29 (? males)	increasing calibration<br>increasing calibration by random increments	**none** (subjects were informed that they would receive electrical stimuli but were not informed about the electrical stimulation protocol)	**no data**	**no data**	random interstimulus interval of 10 to 10 seconds	during each study condition, a total of 45 stimuli were given	no	n/a	pain thresholds<br>150% pain thresholds	**no pretest/baseline**	**no data**<br>in both measurements, each participant indicated when the stimulus was unpleasant, slightly painful (similar to a pinprick)	**no data**	Electrical stimulation was initiated at 1 mA and was increased by 1 mA for each consecutive stimulus.<br>During a second measure, the stimulation was increased by random increments for each consecutive stimulus.<br>In both measurements, each participant indicated when the stimulus was similar to a pinprick (unpleasant, slightly painful).<br>The stimulus intensity was 150% of the average of both pain thresholds
															Calibration using steps of approximately 0.01 mA.

[29]	single-arm	25 (9 males)	the method of limits	**no data**	series of stimuli (5)	1 s	10 s	differently for each participant	if the sensations/intensities could not be matched the entire procedure was restarted.	n/a	target: 10 times the detection threshold	**no pretest/baseline**	**no data**	yes	After having determined detection thresholds, participants were asked to report whether the sensation and intensity of a single pulse were perceived as similar for both forearms. If the percept differed between the two forearms, the intensity of the stimulation was adjusted by slightly increasing or decreasing the intensity of the electrical pulses until the perceived sensation/intensity was matched between both forearms.
[7]	randomized	31 (18 males)	increasing calibration	**no data**	series of stimuli (3)	**no data**	15 s	differently for each participant	no	n/a	target: ≥5/10	4.19	VNS, from 0 - no pain to 10 - the worst pain imaginable	**no data**	The applied current was started at 0 mAmps and progressively increased.<br>At 10-mAmp intervals until the pain was reported as 5 or higher.
[9]	randomized	87 (0 males)	ascending calibration	**no data**	series of stimuli	* 200 µs	**no data**	differently for each participant	2 times	**no data**	targets: tactile threshold (t), threshold (T), 2.2 T and 1.5 T	**no pretest/baseline**	**no data**	**no data**	Calibration was increased by 0.5 mA.<br>The average T value was used to calculate the intensity of the pain stimulus that was to be paired with the nocebo stimulus (2.2 T mA), as well as the pain stimulus that was to be paired with the control stimulus (1.5 T mA).
[27]	single-arm	50 (41 males)	increasing calibration	**no data**	**no data**	**no data**	**no data**	differently for each participant	no	n/a	pain threshold<br>pain tolerance	no data<br>5.3 (2.6)	VAS (1-10)	**no data**	Calibration was increased until the participant verbally tells the moment when the pain first felt. Subsequently, the level
[52]	single-arm	40 (20 males)	increasing calibration	subjects were asked to verbally rate each electric stimulus	series of stimuli (50)	* 1 ms	* 4 ms	differently for each participant	2 times	**no data**	target: intensity corresponding to a rating of 4 on a 5-point scale	**no pretest/baseline**	5- point numerical rating scale (“1 - barely noticeable”, “2 - clearly noticeable but not unpleasant”, “3 - barely unpleasant”, “4 - quite unpleasant” to “5 - very unpleasant”	**no data**	The first electric stimulus was delivered with an intensity of 0.5 mA. Intensity was increased in steps of 0.5 mA until subjects rated the electric stimulus with “4 - quite unpleasant” or until the maximum of 5 mA was reached. This procedure was repeated one more time, resulting in two runs. Later applied an intensity corresponding to a rating of "4 – “quite unpleasant" from the second measurement.
[69]	single-arm	18 (7 males)	**no data**	**no data**	* five trains	* 1 s	* 10 s	**no data**	**no data**	**no data**	target: 20 times the absolute detection threshold	**no pretest/baseline**	**no data**	**no data**	Lots of missing data.
[63]	non-randomized	49 (9 males)	increasing calibration	participants were instructed to pay close attention to the pain stimulus when judging its intensity.	**no data**	**no data**	**no data**	**no data**	**no data**	**no data**	target: moderately intense pain and ‘low intense pain’ stimulus (second stimulus was derived from the moderately intense pain stimulus using the	**no pretest/baseline**	a scale ranging from “no pain”, "little pain", "moderate pain", "intense pain", "enormous pain" and "unbearable pain"	**no data**	Calibration started at 0.5 mA and increased in steps of 0.5 mA. The intensity of the electrocutaneous stimulus increased until participants reported that the pain stimulus they received was of moderate pain on a scale.

											formula)				Lots of missing data.
[48]	single-arm	19 (8 males)	increasing calibration	**no data**	**no data**	1 ms	10 s	differently for each participant	**no data**	**no data**	target: 25% and 75% of unbearable intensity	**no pretest/baseline**	**no data**	**no**	Participants received increasing electrical shock starting from 1 mA with increments of 1 mA until participants felt unbearable.
[78]	single-arm	18 (7 males)	ascending method of limits	**no data**	single stimuli and series of stimuli	* 0.5 ms	**no data**	differently for each participant	no	n/a	target: 2 stimulus magnitudes (1 painful, 1 non-painful) for stim1 and 8 stimulus magnitudes (4 painful, 4 non-painful)	**no pretest/baseline**	VAS (0-100) (anchored at left with “no pain” and at right with “unbearable pain”)	**no data**	The ascending method of limits approach was employed to define the detection threshold and pain threshold. the average of the detection threshold and pain threshold was defined as stim1 for non-pain trials.
			stimulus with a series of stimuli (ascending or descending)					same for each participant							Each trial comprised the stim1 followed by a second non-painful stimulus, which consisted of an ascending or descending series of stimuli (starting from stim; step: 0.3 ma). Two stimulus magnitudes corresponding to the 10th and 90th percentile between stim1 and lownon-pain, and another two stimulus magnitudes between stim1 and highnon-pain, were estimated by linear interpolation.
															The third and fourth steps followed the first and second steps except that stim1 = 50-75 on 100 was measured and defined as the stimulus intensity of stim1 for pain trials, which was followed by two series of pain delayed discrimination trials.
[57]	single-arm	24 (12 males)	increasing calibration	**no data**	**no data**	* 1 ms	5 s	differently for each participant	3 times	differently for each participant	target: pain perception (pp), 150% pp and 180% pp	**no pretest/baseline**	no scale	**no**	The stimulus value was estimated as the average of three trials. The current was increased from a baseline of 0.5 mA in steps of 0.1 mA until the participants reported the stimulation to be painful.
[45]	single-arm	18 (5 males)	**no data**	**no data**	* five trains	* 1 s	* time interval between each train was 10 s	**no data**	**no data**	**no data**	target: 20 times the detection threshold to a single pulse	**no pretest/baseline**	**no data**	*	No detailed description of the calibration procedure allowing to replicate it.
[12]	randomized	419 (187 males)	the method of limits (ascending series)	**no data**	**no data**	**no data**	5 s	differently for each participant	2 times	**no data**	target: tactile and pain thresholds	**no data**	no scale	**no data**	To become accustomed to electrocutaneous stimulation, each participant received the same set of 10 electrical stimuli, ranging from 5 mA to 50 ma, delivered every 5 seconds.
															Two ascending series of electrocutaneous stimuli in increments of 1 mA, starting from 0 mA.

															The obtained values were then averaged separately for tactile (t) and pain thresholds (T) and were used to calculate stimuli at 3 levels of intensity: moderate, low, and high.
[21]	single-arm	20 (10 males)	a staircase procedure (ascending and descending staircases)	**no data**	single stimulus	2 ms	**no data**	differently for each participant	3 times (ascending and descending)	**no data**	target: 10 times the electrical detection threshold	**no pretest/baseline**	no scale	**no**	Each participant was first familiarized with the experimental procedures by receiving a description of the general set-up and the stimuli that they would receive.<br>The final electrical detection threshold was the geometric mean of the three series.
[16]	randomized	60 (24 males)	ascending calibration	**no data**	series of stimuli	**no data**	5 s	differently for each participant	2 times	differently for each participant	target: tactile sensation threshold and pain threshold	**no pretest/baseline**	no scale	**no data**	The intensity of the stimuli was increased by 1 mA.<br>The stimulus intensity for the rest of the experiment was calculated as a doubled mean of the pain threshold.
[70]	single-arm	60 (21 males)	a staircase procedure (increasing and decreasing)	**no data**	**no data**	* 0.5 ms	**no data**	differently for each participant	4 times	**no data**	target: 2 x detection threshold	**no pretest/baseline**	no scale	**no data**	Calibration started from 0.1 mA and increased by 0.1 mA until the first stimulus was detected. Then, the intensity was lowered until no longer perceived, and then increased again. the threshold was established after 3 reversals.<br>The intensity used during the experiments was twice the detection threshold.
[19]	single-arm	14 (9 males)	the method of limits	**no data**	single stimulus	* 1 s	* 10 s	**no data**	**no data**	**no data**	target: 20 times the detection threshold of a single pulse	**no data**	**no data**	**no**	The intensity of stimulation was individually adjusted to 20x the absolute detection threshold to a single pulse.
[11]	randomized	96 (36 males)	ascending calibration	**no data**	**no data**	**no data**	5 s	differently for each participant	2 times	**no data**	target: sensation threshold and pain thresholds	**no pretest/baseline**	no scale	**no data**	The calibration procedure started at 0 mA and increased in 0.5 mA steps.<br>The mean of the two measurements of pain thresholds was calculated and the result subsequently doubled to establish the stimulus intensity that was used throughout the experiment.
[10]	randomized	99 (0 males)	the method of limits (ascending)	**no data**	**no data**	**no data**	5 s	differently for each participant	2 times	**no data**	target: sensation threshold and pain thresholds	**no pretest/baseline**	no scale	**no data**	The calibration procedure started at 0 mA and increased in 0.5 mA steps.
[33]	randomized	70 (24 males)	the staircase procedure	**no data**	single stimulus	2 ms	**no data**	**no data**	**no data**	**no data**	target: 20 times the detection threshold to a single pulse	**no pretest/baseline**	**no data**	**no data**	No detailed description of the calibration procedure allowing to replicate it.

[18]	single-arm	14 (7 males)	the staircase procedure	**no data**	single stimulus	* 1 s	* 10 s	differently for each participant	**no data**	**no data**	target: 20 times the detection threshold to a single pulse	**no pretest/baseline**	no scale	**no data**	The intensity of stimulation was individually adjusted to 20 times the absolute detection threshold to a single pulse.
[75]	single-arm	28 (14 males)	ascending calibration	**no data**	series of stimuli	**no data**	5 s	differently for each participant	3 times	**no data**	target: tactile sensation and the pain threshold	**no pretest/baseline**	no scale	**no data**	The intensity of the pain stimulus was fixed for the whole experiment and was set to 2 T mA (T stands for pain threshold) for each participant.<br>The calibration procedure started at 0 mA and increased in 0.5 mA steps.<br>The averaged value for pain threshold was calculated to determine stimulus intensity for the testing phase of the experiment.
[17]	single-arm	75 (38 males)	the staircase procedure	**no data**	**no data**	2 ms	**no data**	differently for each participant	**no data**	**no data**	target: 20 times the detection threshold of a single pulse	**no pretest/baseline**	no scale	**no**	The intensity of stimulation was individually adjusted to 20 times the absolute detection threshold to a single pulse.
[66]	randomized	56 (0 males)	increasing and decreasing calibration	participants were informed that the experiment was targeting a score of 7 ('moderate pain') on the NRS and that 10 on the scale indicated the intensity that they did not want to receive anymore	**no data**	**no data**	10 s	differently for each participant	no	n/a	target: 7/10 on NRS	**no pretest/baseline**	NRS ranging from 0 –"no pain", to 10 –"the strongest pain ever"	**no**	The calibration procedure started at 0 mA and increased in 1 mA steps to the value at which an NRS score of 7 was obtained. Next, the intensity of electrical stimuli was decreased (0.5 per step) to an NRS score of 6 and next increased until again a value of 7 on the NRS was obtained.<br>The average of the three values of the individually scored stimulus intensity that was rated as a 7 on the NRS was used as painful stimulus during the experiment.
[3]	randomized	75 (0 males)	increasing calibration	**no data**	**no data**	**no data**	5 s	differently for each participant	2 times	**no data**	target: tactile sensation and the pain threshold -> 2T	For placebo condition: G1: 4.17, G2: 4.32, G3: 3.97. For non-placebo condition: G1: 4.27, G2: 4.44, G3: 3.69	no scale	**no data**	The intensity of the pain stimulus was fixed for the whole experiment and was set to 2 T mA (T stands for pain threshold) for each participant.<br>The calibration procedure started at 0 mA and increased in 0.5 mA steps.
[49]	single-arm	12 (7 males)	method of limits (ascending and descending series)	**no data**	single stimulus	**no data**	**no data**	differently for each participant	5 times	**no data**	target: 10 x detection thresholds	**no data**	no scale	**no data**	Individual detection thresholds were determined by the geometric mean of five ascending and five descending series of single pulses.

[62]	randomized	121 (49 males)	ascending	**no data**	* series of five pulses	* 0.2 s	0.1 s (3.5 s between series)	differently for each participant	no	n/a	2, 3, 4, 5, 6, 7, and 8 on NRS	4.43 (1.25)	NRS, 0 = “no pain” to 10 = “the most intense pain that is tolerable”	*	The calibration was divided into two parts. First, an ascending series of stimuli in steps of 1 mA starting at 0 mA were delivered to the participant. The stimulation increased until the stimulus reached 99 mA or induced an intensity of pain that was rated by the participant as 9 on the NRS. Second, the resulting function was used to determine stimuli whose intensities correspond to 2, 3, 4, 5, 6, 7, and 8 points on the NRS. Two identical sequences of pseudorandom stimuli were applied: 5–8–5–2–7–4–6–5–4–4–3–5–6–3–6–7
			pseudorandom					16	2 times	**no data**	5 and 6 on the NRS				The polynomial function was used: STIM(N) = aN2 +bN+c, where N is NRS rating, and STIM(N) is the intensity of the stimuli for the NRS rating N.
[53]	randomized	81 (41 males)	staircase procedure (increasing and decreasing)	**no data**	single stimulus	* 2 ms	**no data**	different for each participant	4 times	**no data**	target: 15 times the detection threshold to a single pulse	**no pretest/baseline**	no scale	**no**	Single electro-cutaneous stimuli were presented one by one (starting at 0.1 ma) in an ascending manner (by 0.1 mA) until a stimulus was detected. Then, the stimuli were presented in a descending manner (by 0.5 mA) until a stimulus was no longer perceived, after which the intensity increased again (by 0.25 mA). The threshold was established after three reversals.
[68]	randomized	138 (69 males)	familiarization phase	volunteers were instructed beforehand, in advance to start the electrical current by pressing the button and to let go of the button as soon as the	**no data**	**no data**	**no data**	different for each participant	no	n/a	target: tolerance threshold	**no pretest/baselin**	* NRS from 0 (no pain) to 10 (worst imaginable pain)	**no data**	The electrical current with a ramping rate of 1 mA per second. The current started at 0 mA and the maximum electrical current was limited to 50 mA.
			increasing calibration								Group 1: NRS 6.5 (2.2)				
											Group 2: NRS 7.1 (1.6)				
[71]	randomized	44 (0 males)	increasing and decreasing calibration	**no data**	single stimulus	2 ms	**no data**	different for each participant	4 times	**no data**	20 x the individual detection threshold	**no pretest/baseline**	* VAS from 0 (“not unpleasant at all”) to 100 (“as unpleasantness as possible”)	**no**	Single electrocutaneous stimuli were administered one by one, starting at 0.1 mA with increasing steps of 0.1 mA. Once the stimulus was detected, stimuli were presented in decreasing steps of 0.05 mA until the stimulus was no longer perceived, after which the intensity increased again in steps of 0.025 mA. After three reversals, the detection threshold was established.

[35]	single-arm	20 (11 males)	**no data**	**no data**	**no data**	* 0.5 ms	* 5 ms	**no data**	**no data**	**no data**	threshold<br>about double the threshold but not perceived as uncomfortable or painful<br>mild, moderate, or marked discomfort (NRS 1, 2, and 3)<br>clear pain (NRS 4–10)	**no data**	* NRS	**no data**	Lots of missing data.
[54]	single-arm	30 (2 males)	familiarization phase (ascending series)<br>ascending series<br>random series	**no data**	**no data**	* 1000 ms	**no data**	different for each participant	no	n/a	no pain, moderate pain, and very high pain	**no data**	NRS (0 - no pain at all, 2 - low pain, 4 - moderate pain, 6 - high pain, 8 - very high pain, and 10 - the most intense pain imaginable)	**no data**	The electrical pain calibration procedure consisted of three steps. Step 1 was the familiarization phase. Participants received an ascending series of electrical stimuli, starting at 0.5 mA and increasing with steps of 0.5 mA. Participants indicated verbally the first time they felt the stimulation (perception threshold), when the stimulation first became painful (pain
[28]	single-arm	12 (6 males)	increasing calibration	**no data**	single stimulus	* 0.2 ms	**no data**	different for each participant	**no data**	**no data**	detection threshold, pain threshold, and pain 4/10 (10 x detection threshold)	**no pretest/baseline**	NRS; 0: no pain, 10: maximum pain	**no**	Single electrical pulses steps of 0.1 mA were applied until the subjects reported electrical sensation, pain, and a pain rating of 4/10, respectively. additionally, each subject was familiarized with the electrical pulses prior to assessment of electrical pain threshold.<br>Mean stimulus intensity during calibration 1.2±0.8 mA.<br>The electrical Test Stimuli was presented as 3 pulses (2ms pulse width) applied at 0.2Hz for repeated at 5min intervals during 15min prior to HFS .
[43]	single-arm	33 (0 males)	staircase procedurę (increasing and decreasing)	**no data**	**no data**	**no data**	**no data**	**no data**	4 times	**no data**	10 x detection threshold	**no pretest/baseline**	**no data**	**no data**	A staircase procedure - a low intensity was presented at first and then was gradually increased (1 mv step size) until the participant was able to detect the stimulus. The intensity was then lowered again until the participant was unable to detect the stimulus. The threshold was established after 3 such reversals.
[34]	single-arm	20 (4 males)	method of limits	**no data**	single stimulus	2 ms	**no data**	different for each participant	**no data**	**no data**	20 x the individual detection threshold	**no pretest/baseline**	**no data**	**no**	Mean stimulus intensity during calibration (SD) 0.32 (0.09).<br>Each electrical pulse lasted one second and was delivered in a 10-second interval - in main procedure.
[illegible]	single-arm	[illegible]	[illegible]		[illegible]			different for [illegible]			detection threshold				[illegible]

[64]	single-arm	23 (11 males)	increasing calibration	**no data**	single stimulus	1 ms	**no data**	each participant	2 times	**no data**	pain threshold<br>3 on NRS	**no pretest/baseline**	NRS	**no**	Rectangular pulses with increasing intensities (0.2 mA/sec) were applied.

* It is unclear whether the data relates only to the main or the whole procedure (including calibration)
